# The most bothersome symptoms in neuromuscular diseases: the ERN EURO NMD Survey

**DOI:** 10.1186/s13023-025-03742-z

**Published:** 2025-05-08

**Authors:** Michelangelo Mancuso, Alessandro Colitta, Manuela Lavorato, Peter Van den Bergh, Janbernd Kirschner, Cornelia Kornblum, Lorenzo Maggi, Francois Lamy, Hanns Lochmüller, Marianne Nordstrøm, Edoardo Malfatti, Alessandra Ferlini, Davide Pareyson, Vincenzo Silani, Kleopas A Kleopa, Marianne de Visser, Antonio Atalaia, Teresinha Evangelista

**Affiliations:** 1 Department of Clinical and Experimental Medicine, Neurological Institute, University of Pisa, Pisa, Italy; 2 Neurological Institute, Azienda Ospedaliero Universitaria Pisana, Pisa, Italy; 3 Neuromuscular Reference Centre, Department of Neurology, University Hospital Saint- Luc, Brussels, Belgium; 4 Department of Neuropediatrics and Muscle Disorders, Medical Center, Faculty of Medicine, University of Freiburg, Freiburg, Germany; 5 Department of Neuromuscular Disorders, Center for Neurology, University Hospital, Bonn, Germany; 6 Neuroimmunology and Neuromuscular Diseases Unit, Fondazione IRCCS Istituto Neurologico Carlo Besta, Milan, Italy; 7 Association Française Contre Les Myopathies, AFM-Téléthon, Evry, France; 8 Centro Nacional de Análisis Genómico (CNAG), Barcelona, Catalonia Spain; 9 Division of Neurology, Department of Medicine, The Ottawa Hospital; and Brain and Mind Research Institute, Children’s Hospital of Eastern Ontario Research Institute, University of Ottawa, Ottawa, Canada; 10 Unit for Inborn and Hereditary Neuromuscular disorders, Department of Neurology, Oslo University Hospital, Oslo, Norway; 11 Frambu Resource Centre for Rare Disorders, Siggerud, Norway; 12 Reference Center for Neuromuscular Disorders, APHP Henri Mondor Hospital, University Paris Est, Inserm, U955, IMRB, Créteil, F-94010 France; 13 Unit of Medical Genetics, Department of Medical Science, University of Ferrara, Ferrara, Italy; 14 Rare Neurological Diseases Unit, Department of Clinical Neurosciences, Fondazione IRCCS Istituto Neurologico Carlo Besta, Milan, Italy; 15 Department of Neuroscience and Laboratory of Neuroscience, IRCCS Istituto Auxologico Italiano, Milan, Italy; 16 “Dino Ferrari” Center, Department of Pathophysiology and Transplantation, Università degli Studi di Milano, Milan, Italy; 17 Department of Neuroscience and Center for Neuromuscular Disorders, The Cyprus Institute of Neurology and Genetics, Nicosia, Cyprus; 18 Department of Neurology, location AMC, Amsterdam University Medical Center, Neuroscience Institute, Amsterdam, The Netherlands; 19 Center of Research in Myology Inserm UMRS UMRS 974, APHP G.H. Pitie-Salpetriere, Sorbonne Université, Paris, France; 20 Muscle Pathology Unit, Institute of Myologie and Neuropathology department Pitié- Salpêtrière Hospital, APHP-Sorbonne Université, Paris, France

**Keywords:** Neuromuscular diseases, Symptoms, PROMs

## Abstract

**Background:**

Neuromuscular diseases (NMDs) comprise a range of genetic and acquired rare disorders that affect motor neurons, peripheral nerves, neuromuscular junctions and skeletal muscles, leading to significant impairments such as muscle weakness and fatigue resulting in functional limitations. This study aims to investigate the prevalence and severity of disease-related symptoms in adult patients with NMDs registered in the European Reference Network (ERN) EURO-NMD. A cross-sectional electronic survey was conducted with 1,253 participants who reported the severity of 28 symptoms, which were scored using multi-criteria decision analysis (MCDA).

**Results:**

The results identified muscle fatigue, weakness and impaired physical function/activity as the most severe and prevalent symptoms in all NMD groups, followed by coordination and/or balance problems, muscle stiffness, mental fatigue, and pain. Notably, the analysis highlighted differences in symptom severity between disease subtypes and underlined the need for standardised patient-reported outcome measures (PROMs) to address the broad heterogeneity of NMDs.

**Conclusions:**

The findings stress the critical importance of capturing patient perspectives to guide clinical care, research priorities and therapeutic development. This work argues for the development of uniform PROMs to better assess disease impact, natural history and treatment efficacy, contributing to improved patient-centred care across diverse NMD populations.

**Supplementary Information:**

The online version contains supplementary material available at 10.1186/s13023-025-03742-z.

## Introduction

Neuromuscular disorders (NMDs) are caused by acquired or genetic rare defects of motor neurons, peripheral nerves, neuromuscular junction or skeletal muscle, and are often associated with muscle weakness and wasting, impaired muscle endurance, involuntary muscle activity (stiffness, cramps, and fasciculations), impaired control of voluntary movements, sensory disturbances, autonomic dysfunction, dysphagia, cognitive, respiratory and cardiac failure. Individual NMDs are rare, but as a group they are not. The overall annual incidence rate of neuromuscular disorders is reported to be 122 per 100,000 population based on health insurance billing codes within administrative health databases in Ontario, Canada [1]. NMDs collectively affect an estimated 500,000 EU citizens and result in significant costs for families and the healthcare system. The European Reference Network (ERN) EURO-NMD unites 82 of Europe’s leading NMD clinical and research centres in 25 Member States and includes highly active patient organizations (https://ern-euro-nmd.eu/). More than 100,000 NMD patients are seen annually by the ERN [2].

An important goal for a clinician treating people with NMD is to reduce the burden of the disease by treating the troublesome symptoms that patients often experience, such as weakness, pain, fatigue, and others. In fact, several symptoms affect NMD patients’ daily functioning, and, in turn, their quality of life (QoL)^2^. To measure the impact of such symptoms on patients’ perceived health, patient-reported outcome measures (PROMs) are routinary adopted in both clinical practice and research. Additionally, PROMs can trace disease progression from the patient perspective, which may differ from that of the clinicians [3]. In the field of PROMs, disease-specific questionnaires are designed to target unique clinical features of single NMDs [3–7]. However, their use is restricted to the specific NMD they were designed for, limiting their broader applicability [8]. In contrast, generic PROMs have been developed, although they show lower sensitivity to changes in patients’ conditions [2, 8–10]. Within this context, identifying the most bothersome symptoms across different NMD populations may foster the creation of a unique PROM for NMDs, investigating both common and disease-specific bothersome symptoms. The aim of this study was to examine the prevalence and severity of a large number of symptoms and disease-related disabilities in patients diagnosed with all NMDs followed at the ERN EURO-NMD.

## Methods

Several online meetings were organized between the Patient Advisory Board (PAB, https://ern-euro-nmd.eu/group/patient-advisory-board/) and the Executive Committee (https://ern-euro-nmd.eu/group/executive-committee/) of the ERN EURO-NMD to develop a comprehensive list of symptoms that could potentially affect NMD patients (bothersome symptoms). Following multiple rounds of emails, the final list of symptoms, each with a detailed explanation, has been approved in both Italian and English language (Supplementary File 1). In its final version, the ERN EURO NMD Survey investigates 28 symptoms, along with information about gender (Male, Female, Prefer not to say), age range (18–25, 26–35, 36–45, 46–55, 56–69, 70 and older), country of residency and name of the disease (self-reported; Supplementary File 1).

After the survey approval, a cross-sectional study was conducted. The electronic survey was administered to adult patients diagnosed with a NMD using the SurveyMonkey platform from January 20th to April 30th, 2024, according to a single-stage sampling scheme. Multiple responses by a single participant were not allowed on the SurveyMonkey platform [11]. The survey circulated through the ERN EURO NMD channels, including PAB channels. Data was anonymized, including the participant’s IP address. No genetic or clinical data were obtained or recorded in any form. Uncomplete responses to the survey were excluded from data analysis.

For each symptom, the patient was supposed to grade the severity on a scale ranging from 0 (no bother at all), 1 (mild), 2 (moderate) to 3 (severe). Symptoms rated as moderate [2] or severe [3] were classified as bothersome, and the percentage of patients reporting these symptoms as bothersome was calculated for the entire cohort.

We conducted a multi-criteria decision analysis (MCDA) to rank patients’ symptoms based on their reported severity, accounting for both the possible variation of symptom severity across different NMDs and the possible uneven distribution of patients across different NMDs. MCDA is a quantitative method used to rank a set of alternatives based on multiple decision criteria which may have different weights in the decision process [12, 13]. In this study, the alternatives were represented by the symptoms investigated by the questionnaire. We identified the reported severity of each symptom as the decision criteria to rank the symptoms. In parallel, the number of participants in each NMD group was used to equally weight each NMD group in the ranking process. The MCDA was operationally carried out as follows [12, 13]:

1. By filling the EURO-NMD questionnaire, participants scored the perceived severity of the investigated symptoms based on the following 4-levels ordered scale: 0 (“none”), 1 (“mild”), 2 (“moderate”), 3 (“severe”).
2. Participants were categorized by their NMD, and severity scores for each symptom were aggregated within each group. Weighted severity scores within each group were subsequently obtained by dividing the aggregated severity scores of each symptom by the number of participants in the considered NMD group.
3. The weighted severity scores of each symptom were summed across all NMDs to obtain the MCDA Severity Score of each symptom. Finally, the symptoms were ordered based on their MCDA severity score.

To exclude that underrepresented NMDs (*N* < 30), the “I do not know my disease” group, and the “Other NMDs” group were biasing our results, a sensitivity analysis was performed by repeating the MCDA on a reduced sample excluding the aforementioned group [14]. Differences in reported symptom severity between age groups and sexes were tested through post-hoc chi-squared tests in the entire cohort and within each NMD group, excluding underrepresented groups and the “Other NMDs” group. The results of this study were reported in accordance with the consensus-based Checklist for Reporting of Survey Studies (CROSS) [15].

## Results

**Fig. 1:**
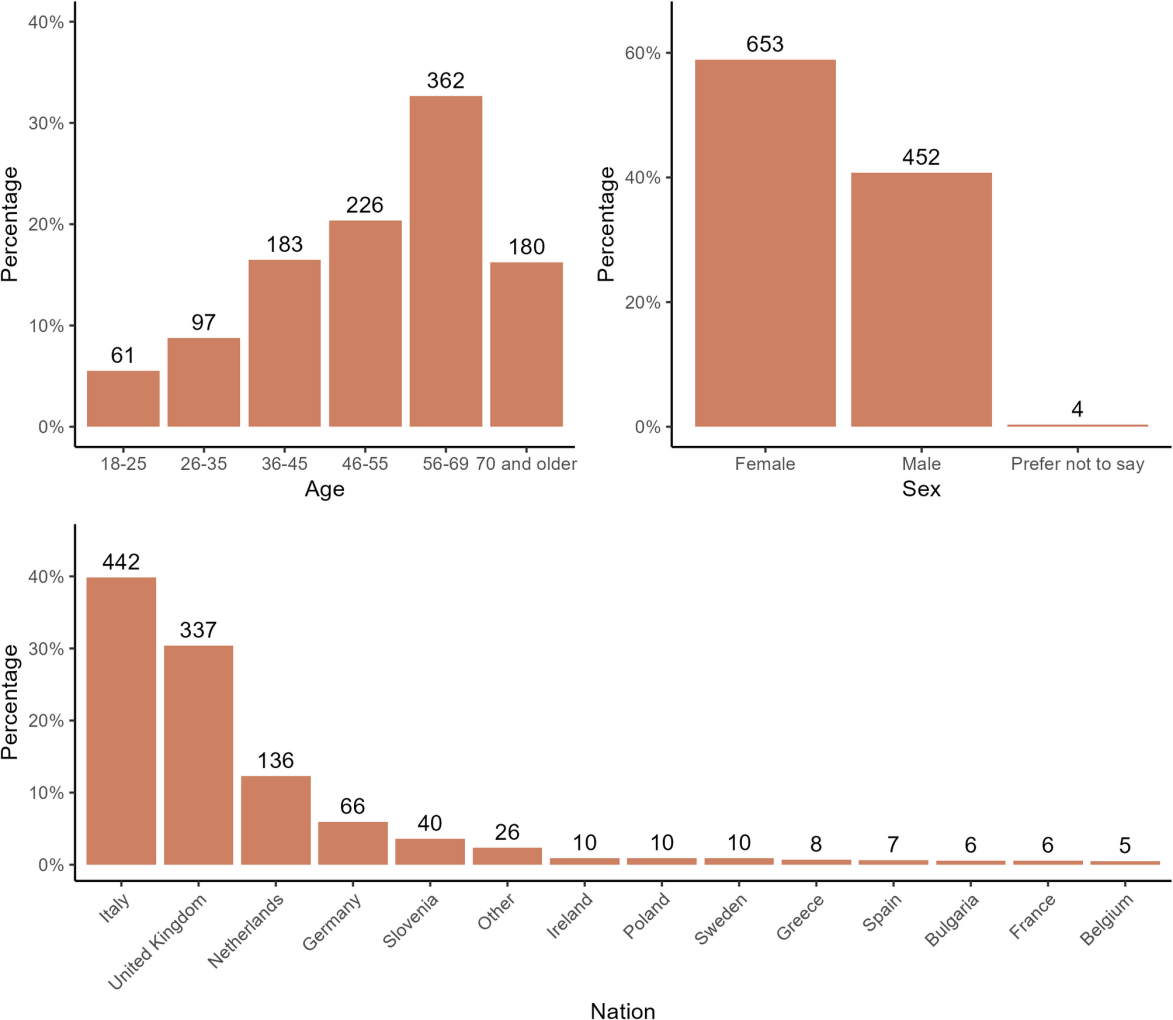
Distribution of age, sex, and nationality among recruited neuromuscular disease patients. On the y-axis: percentage of patients; above the bars: number of patients. Nations included in the ‘Other’ group: Finland, Austria, Croatia, Denmark, Romania, Czech Republic, Lituania

The survey was filled in by 1,253 patients, with 1,109 complete responses being gathered. Demographics of participants are reported in Fig. 1. In detail, the 59–69 years old age group was the most represented, with 60% of the participants being females. The most represented countries of residency were Italy (*N* = 442, 40%), United Kingdom (*N* = 337, 30%), and Netherlands (*N* = 136, 12%).

The participants represented all NMDs covered by the ERN EURO-NMD network. Two disease groups, primary mitochondrial diseases -PMDs- and Charcot-Marie-Tooth neuropathies -CMT- were the most represented (19.5 and 25.6% of total responders respectively), while neuropathies associated with haematological disease and monoclonal gammopathy and skeletal muscle channelopathies were the least, as shown in Table 1.

**Table 1:** Distribution of participants across different neuromuscular diseases

Neuromuscular Disease	*N* = 1109^*1*^
Charcot-Marie-Tooth disease and related neuropathies (HNNP, HSAN, dHMN)	295 (26.6%)
Mitochondrial Diseases	216 (19.5%)
Other	111 (10%)
Spinal Muscular Atrophy (SMA)	70 (6.3%)
Other Muscular Dystrophies (excluding Duchenne, Becker, FSHD, myotonic dystrophies)	65 (5.9%)
Facioscapulohumeral Muscular Dystrophy (FSHD)	62 (5.6%)
Duchenne or Becker Muscular Dystrophy	48 (4.3%)
Myotonic Dystrophies	46 (4.1%)
Myasthenia gravis	41 (3.7%)
Idiopathic Inflammatory Myopathies	31 (2.8%)
Congenital Myopathies and Congenital muscular dystrophies	22 (2%)
Small Fiber Neuropathies	18 (1.6%)
Metabolic Myopathies	17 (1.5%)
Congenital Myasthenic Syndromes	14 (1.3%)
Inflammatory and Dysimmune Neuropathies	14 (1.3%)
Amyotrophic Lateral Sclerosis and other motor neuron diseases (excluding SMA)	10 (0.9%)
Idiopathic Neuropathies	10 (0.9%)
‘I do not know the name of my disease’	8 (0.7%)
Myofibrillar Myopathies	6 (0.5%)
Neuropathies associated with hematological disease and monoclonal gammopathy (MGUS, POEMS, etc.)	4 (0.4%)
Skeletal Muscle Channelopathies	1 (0.1%)

^*1*^n (%)

**Fig. 2:**
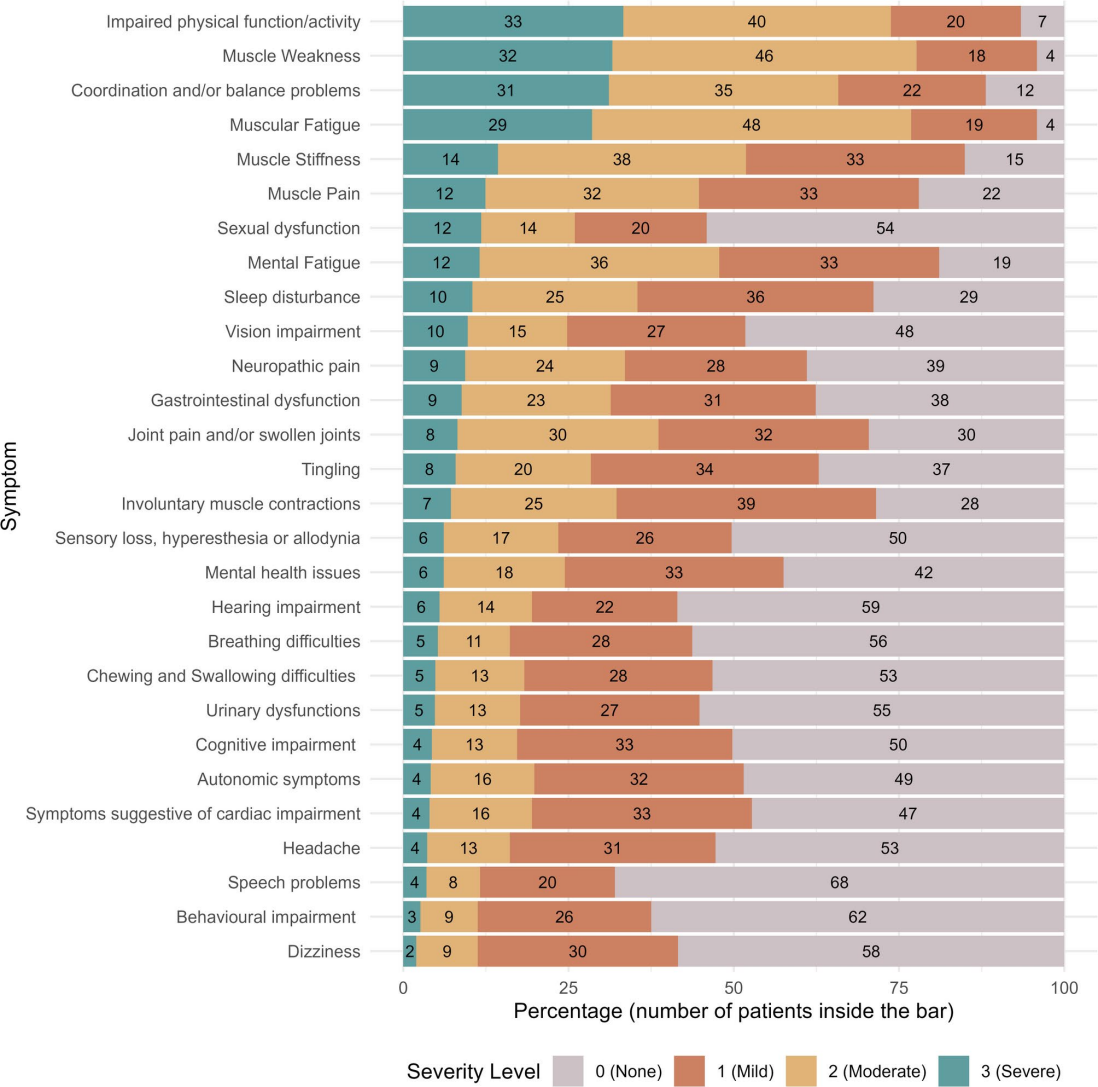
Patients’ severity scores of each symptom investigated by the EURO-NMD PRO Survey

Overall, the most bothersome symptoms reported by the majority of the participants were muscle weakness (78% of responders), muscle fatigue (77%), impaired physical function/activity (74%), coordination and/or balance problems (66%), muscle stiffness (52%), muscle pain (44%) and mental fatigue (48%) as shown in Fig. 2. The supplementary figures S1 show the patients’ severity score of each symptom within each NMD group and differences in severity scores between age groups and sexes in the entire cohort and within each NMD group, excluding under-represented NMD groups and the “Other NMDs” group. The group “Prefer not to say” was excluded from post-hoc analysis regarding sex due to its low representation (*N* = 4). When considering the entire cohort, older age groups were associated with increased severity of 9 out of the 28 investigated symptoms. Conversely, severity of headache, behavioural dysfunction, and mental health issues was negatively associated with age (*p* < 0.001, *p* = 0.005, and *p* = 0.003, respectively). Furthermore, significant differences in symptoms severity were found between sexes, with men reporting higher severity of 4 out of 28 investigated symptoms (coordination and/or balance problems, impaired physical function, sexual dysfunction, hearing impairment), while females showed higher severity of muscular fatigue, mental fatigue, dizziness, cognitive impairment, muscle pain, neuropathic pain, headache, and gastrointestinal dysfunction.

Weighted severity scores for each symptom within each NMD group are shown in Supplementary Table S1. Based on weighted symptom severity scores, the most severe symptom within each NMD group is reported in Table 2. Muscle Fatigue was most frequently reported as the most severe symptom across different NMDs (number of NMD groups = 8, 33%), followed by impaired Physical Function/Activity (number of NMD groups = 6, 25%) and muscle weakness (number of NMD groups = 4, 16.7%).

**Table 2:** Most severe symptoms reported within each neuromuscular disease group

Symptom	Diseases (weighted severity score of the symptom)	Number of diseases (Percentage)
Muscular Fatigue	Idiopathic Inflammatory Myopathies (2), Inflammatory and Dysimmune Neuropathies (2.4), Metabolic Myopathies (2.1), Mitochondrial Diseases (2.2), Myasthenia gravis (2), Other (2), ‘I do not know the name of my disease’ (2), Skeletal Muscle Channelopathies (3)	8 (33.3%)
Impaired physical function/activity	Amyotrophic Lateral Sclerosis and other motor neuron diseases (excluding SMA) (2.3), Congenital Myasthenic Syndromes (1.9), Congenital Myopathies and Congenital muscular dystrophies (1.8), Duchenne or Becker Muscular Dystrophy (2.3), Other Muscular Dystrophies (excluding Duchenne, Becker, FSHD, myotonic dystrophies) (2.4), Spinal Muscular Atrophy (SMA) (2.5)	6 (25%)
Muscle Weakness	Amyotrophic Lateral Sclerosis and other motor neuron diseases (excluding SMA) (2.3), Facioscapulohumeral Muscular Dystrophy (FSHD) (2.2), Myofibrillar Myopathies (2.5), Myotonic Dystrophies (2.2)	4 (16.7%)
Autonomic symptoms	Neuropathies associated with haematological disease and monoclonal gammopathy (MGUS, POEMS, ETC) (2.2)	1 (4.2%)
Coordination and/or balance problems	Charcot-Marie-Tooth disease and related neuropathies (HNNP, HSAN, dHMN) (2.3)	1 (4.2%)
Joint pain and/or swollen joints	Neuropathies associated with haematological disease and monoclonal gammopathy (MGUS, POEMS, ETC) (2.2)	1 (4.2%)
Mental Fatigue	Skeletal Muscle Channelopathies (3)	1 (4.2%)
Neuropathic Pain	Small Fibre Neuropathies (2.6)	1 (4.2%)
Tingling	Idiopathic Neuropathies (2.1)	1 (4.2%)

**Fig. 3:**
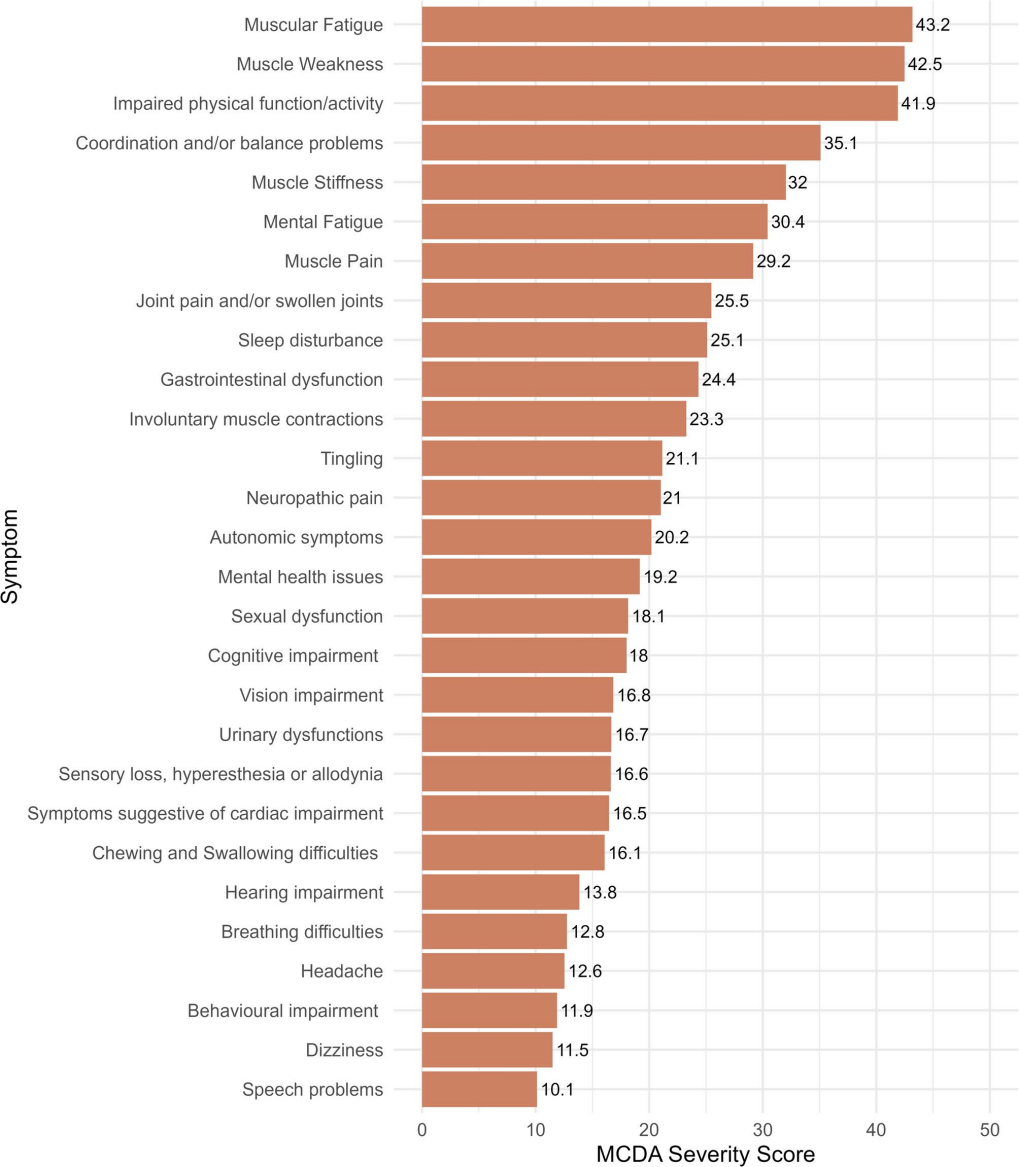
Multi-Criteria Decision Analysis (MCDA) Severity Scores of symptoms

When MCDA Severity Scores were calculated, muscular fatigue emerged as the most severe symptom across all NMD groups (MCDA Severity Score = 43.2), followed by muscular weakness (MCDA Severity Score = 42.5) and impaired physical function/activity (MCDA Severity Score = 41.9), as shown in Fig. 3.

**Fig. 4:**
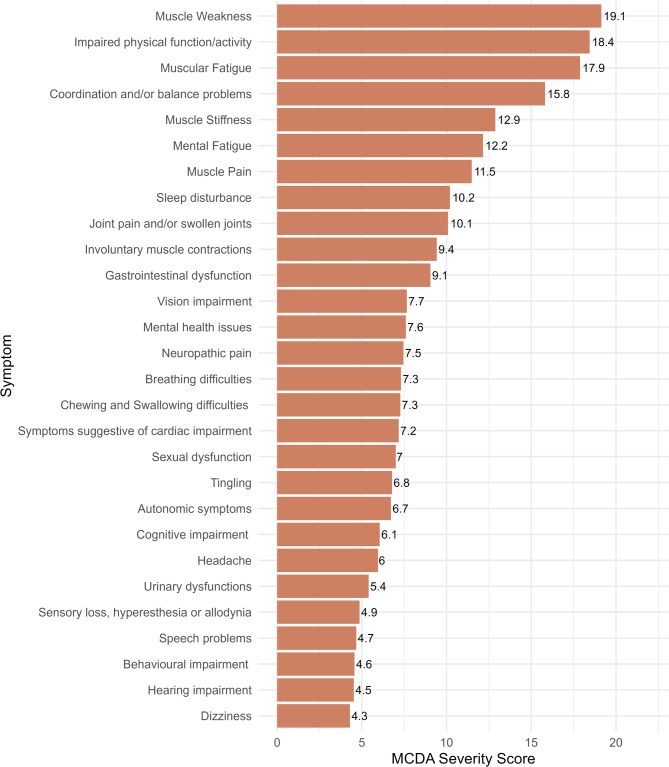
Multi-Criteria Decision Analysis (MCDA) Severity Scores of symptoms calculated excluding underrepresented neuromuscular diseases, the Other’ group, and the “I do not know my disease” group

When excluding underrepresented NMD groups, i.e., the “I do not know my disease” group, and the “Other” group (number of excluded patients = 340, number of included patients = 769, Fig. 4), the 3 most severe symptoms remained Muscular Weakness (MCDA Severity Score = 19.1), Impaired Physical Function/Activity (MCDA Severity Score = 18.4) and Muscular Fatigue (MCDA Severity Score = 17.9), although their ranking differed from the ranking obtained performing the MCDA on the entire sample. From the 4th to the 7th symptoms’ ranking was unchanged: Coordination and/or Balance Problems (MCDA Severity Score = 15.8), Muscle Stiffness (MCDA Severity Score = 12.9), Mental Fatigue (MCDA Severity Score = 12.2), Muscle Pain (MCDA Severity Score = 11.5).

## Discussion

The goal of this study was to determine the prevalence, severity, and impact of a broad range of disease-related symptoms in a large cohort of adult patients with NMDs. We believe this is essential to inform clinicians, researchers, advocacy and regulatory partners, and pharmaceutical companies about the needs and expectations of NMD research subjects for epidemiologic and natural history studies and clinical trial participation. Particularly striking was the discovery that subjects from different NMD cohorts reported experiencing multiple disabling major clinical symptoms. This finding underscores the profound burden of NMD on patients, families, caregivers, and the healthcare system.

The present study has several distinctive strengths. Primarily, it examines the impact of a comprehensive range of disease-related symptoms in all diseases covered by the ERN EURO-NMD, taking into account also patients’ representatives’ feedback. This approach differs from that of most previous studies [4–7, 16], which have focused on a limited number of disabling symptoms within a specific category of NMDs. Consequently, this study provides a unique and valuable insight into the consequences of NMDs as a group of diseases. Furthermore, our analytical method allowed us to rank investigated symptoms based on their patient-reported severity, irrespective of both the possible variation of symptom severity across different NMDs and the possible uneven distribution of patients across different NMDs. In our cohort of adult NMD patients, the 3 most severe were muscle weakness, impaired physical function/activity and muscle fatigue, followed by coordination and/or balance problems, muscle stiffness, mental fatigue and muscle pain. Furthermore, our study provides further insights into the most bothersome symptoms within each NMD group (Supplementary Table S1), which are coherent with previous literature [4–7], e.g., impaired balance and coordination, muscle weakness and fatigue in CMT1A [5]; impaired physical function/activity, muscle weakness and fatigue in Amyotrophic Lateral Sclerosis, facioscapulohumeral muscular dystrophy and idiopathic inflammatory myopathies [4, 6, 7]; and vision impairment, muscle weakness and fatigue for mitochondrial diseases [17, 18]. Finally, our research shows higher severity of 9 out of 28 investigated symptoms in older age groups. Contrarily, severity of headache, behavioural dysfunction, and mental health issues decreased with age. While the decrease in headache severity is a known phenomenon [19], the observed decrease in behavioural dysfunction and mental health issues may depend on a lower insight on these conditions shown by older patients [20]. As a whole, these data may be helpful in identifying reliable clinical outcomes and reliable measures to assess disease progression and response to (any) treatment which are the most fundamental steps in natural history studies and trial design. In addition, obtaining the patient’s perspective on their most prevalent and disabling symptom(s) that they themselves prioritize for treatment is essential in NMDs that have such high phenotypic heterogeneity. Combined with our finding that disease-related disabilities rather than the specific medical diagnosis determine the magnitude of disease burden [4–7, 16], our results are relevant to a broader population and could have important implications for the treatment of patients with chronic diseases such as NMD.

Another important message from this survey is that we could envision the development of a unique, cross-cutting PROM for all NMDs, covering both common bothersome symptoms across different NMDs and single bothersome symptoms which characterize single NMDs. In the field of NMDs, several generic and disease-specific PROMs are routinary adopted [3]. A minority of these have been developed to be horizontally used across different NMDs [3, 8–10]. However, NMD-specific PROMs mainly investigate muscle weakness, pain, dysphagia, and fatigue [3, 8–10], which represent only part of the most bothersome NMD symptoms found in the present study. Our findings indicate that a PROM for NMDs may be a viable approach if developed including both common bothersome symptoms across different NMDs and single bothersome symptoms which characterize single NMDs.

This study is limited by the lack of verification of a patient-reported diagnosis. Moreover, robust conclusions cannot be achieved in secondary analyses involving the relatively high number of underrepresented NMD groups. Furthermore, in these NMD groups, the observed demographics may not accurately reflect the demographics of the reference population. Limitations of this study also include the lack of a thorough definition of either symptom severity levels or the symptom “impaired physical function/activity” in the survey text. Finally, the proportion of participants included in each NMDs may not mirror the prevalence of such NMDs in the general population.

## Electronic supplementary material

Below is the link to the electronic supplementary material.

Supplementary Material 1

Supplementary Material 2

Supplementary Material 3

## Data Availability

The anonymized dataset used and/or analyzed during the current study is available from the corresponding author on reasonable request.
