# Supplementary material for: The most bothersome symptoms in neuromuscular diseases: the ERN EURO NMD Survey
Source: Orphanet J Rare Dis. 2025 May 8;20:221. doi: 10.1186/s13023-025-03742-z (PMC12063438; doi:10.1186/s13023-025-03742-z)
Supplement: Supplementary file 1 — Supplementary Material 1 [file 13023_2025_3742_MOESM1_ESM.pdf]

SUPPLEMENTARY TABLE 1: Weighted severity scores of symptoms investigated by the EURO-PRO Questionnaire within each neuromuscular disease group

Disease	Symptom	Weighted symptoms severity scores
Amyotrophic Lateral Sclerosis and other motor neuron diseases (excluding SMA)	Muscle Weakness	2.30
	Impaired physical function/activity	2.30
	Coordination and/or balance problems	2.10
	Muscular Fatigue	1.70
	Involuntary muscle contractions	1.70
	Muscle Stiffness	1.50
	Speech problems	1.30
	Muscle Pain	1.20
	Mental Fatigue	1.10
	Sexual dysfunction	1.10
	Joint pain and/or swollen joints	0.80
	Neuropathic pain	0.80
	Chewing and Swallowing difficulties	0.70
	Sleep disturbance	0.70
	Gastrointestinal dysfunction	0.70
	Urinary dysfunctions	0.70
	Breathing difficulties	0.60
	Mental health issues	0.60
	Behavioural impairment	0.60
	Symptoms suggestive of cardiac impairment	0.50
	Tingling	0.50
	Headache	0.40
	Cognitive impairment	0.40
	Autonomic symptoms	0.40
	Sensory loss, hyperesthesia or allodynia	0.30
	Dizziness	0.20
	Vision impairment	0.00
	Hearing impairment	0.00
Charcot-Marie-Tooth disease and related neuropathies (HNNP, HSAN, dHMN)	Coordination and/or balance problems	2.29
	Muscle Weakness	2.11
	Impaired physical function/activity	2.01
	Muscular Fatigue	1.95
	Muscle Stiffness	1.58
	Tingling	1.44
	Muscle Pain	1.38
	Neuropathic pain	1.38
	Mental Fatigue	1.37
	Joint pain and/or swollen joints	1.32
	Sensory loss, hyperesthesia or allodynia	1.29
	Involuntary muscle contractions	1.18
	Sleep disturbance	1.14

SUPPLEMENTARY TABLE 1: Weighted severity scores of symptoms investigated by the EURO-PRO Questionnaire within each neuromuscular disease group

Disease	Symptom	Weighted symptoms severity scores
	Mental health issues	0.86
	Sexual dysfunction	0.80
	Hearing impairment	0.79
	Gastrointestinal dysfunction	0.74
	Symptoms suggestive of cardiac impairment	0.62
	Urinary dysfunctions	0.61
	Autonomic symptoms	0.59
	Cognitive impairment	0.56
	Vision impairment	0.52
	Dizziness	0.46
	Headache	0.44
	Breathing difficulties	0.38
	Chewing and Swallowing difficulties	0.33
	Behavioural impairment	0.33
	Speech problems	0.19
Congenital Myasthenic Syndromes	Impaired physical function/activity	1.86
	Muscle Weakness	1.71
	Muscular Fatigue	1.64
	Vision impairment	1.50
	Muscle Stiffness	0.86
	Joint pain and/or swollen joints	0.86
	Chewing and Swallowing difficulties	0.86
	Mental Fatigue	0.64
	Muscle Pain	0.64
	Symptoms suggestive of cardiac impairment	0.64
	Involuntary muscle contractions	0.64
	Sleep disturbance	0.64
	Gastrointestinal dysfunction	0.64
	Mental health issues	0.57
	Tingling	0.57
	Breathing difficulties	0.50
	Headache	0.50
	Hearing impairment	0.36
	Speech problems	0.36
	Autonomic symptoms	0.36
	Coordination and/or balance problems	0.29
	Urinary dysfunctions	0.21
	Neuropathic pain	0.14
	Cognitive impairment	0.14
	Behavioural impairment	0.14
	Sexual dysfunction	0.14

SUPPLEMENTARY TABLE 1: Weighted severity scores of symptoms investigated by the EURO-PRO Questionnaire within each neuromuscular disease group

Disease	Symptom	Weighted symptoms severity scores
	Dizziness	0.07
	Sensory loss, hyperesthesia or allodynia	0.07
Congenital Myopathies and Congenital muscular dystrophies	Impaired physical function/activity	1.82
	Muscular Fatigue	1.73
	Muscle Weakness	1.59
	Muscle Stiffness	1.27
	Coordination and/or balance problems	1.27
	Sleep disturbance	1.09
	Mental Fatigue	0.95
	Gastrointestinal dysfunction	0.95
	Muscle Pain	0.86
	Joint pain and/or swollen joints	0.86
	Involuntary muscle contractions	0.77
	Symptoms suggestive of cardiac impairment	0.73
	Autonomic symptoms	0.73
	Vision impairment	0.64
	Chewing and Swallowing difficulties	0.59
	Breathing difficulties	0.59
	Cognitive impairment	0.59
	Tingling	0.59
	Headache	0.55
	Mental health issues	0.55
	Neuropathic pain	0.50
	Behavioural impairment	0.45
	Hearing impairment	0.41
	Urinary dysfunctions	0.41
	Sexual dysfunction	0.36
	Sensory loss, hyperesthesia or allodynia	0.32
	Speech problems	0.18
	Dizziness	0.18
Duchenne or Becker Muscular Dystrophy	Impaired physical function/activity	2.29
	Muscle Weakness	2.27
	Muscular Fatigue	1.75
	Coordination and/or balance problems	1.62
	Muscle Stiffness	1.33
	Muscle Pain	1.19
	Mental Fatigue	1.12
	Symptoms suggestive of cardiac impairment	1.02
	Breathing difficulties	1.02
	Joint pain and/or swollen joints	0.96
	Chewing and Swallowing difficulties	0.83

SUPPLEMENTARY TABLE 1: Weighted severity scores of symptoms investigated by the EURO-PRO Questionnaire within each neuromuscular disease group

Disease	Symptom	Weighted symptoms severity scores
	Sleep disturbance	0.83
	Involuntary muscle contractions	0.79
	Gastrointestinal dysfunction	0.77
	Neuropathic pain	0.56
	Autonomic symptoms	0.56
	Vision impairment	0.54
	Mental health issues	0.54
	Tingling	0.54
	Speech problems	0.52
	Headache	0.48
	Behavioural impairment	0.46
	Cognitive impairment	0.42
	Sexual dysfunction	0.42
	Dizziness	0.27
	Sensory loss, hyperesthesia or allodynia	0.25
	Urinary dysfunctions	0.23
	Hearing impairment	0.10
Facioscapulohumeral Muscular Dystrophy (FSHD)	Muscle Weakness	2.21
	Impaired physical function/activity	2.13
	Muscular Fatigue	1.97
	Coordination and/or balance problems	1.82
	Muscle Stiffness	1.63
	Muscle Pain	1.55
	Mental Fatigue	1.34
	Joint pain and/or swollen joints	1.34
	Involuntary muscle contractions	1.02
	Sleep disturbance	0.90
	Neuropathic pain	0.89
	Mental health issues	0.87
	Gastrointestinal dysfunction	0.79
	Headache	0.71
	Sexual dysfunction	0.71
	Tingling	0.69
	Symptoms suggestive of cardiac impairment	0.68
	Cognitive impairment	0.63
	Autonomic symptoms	0.61
	Vision impairment	0.60
	Breathing difficulties	0.58
	Hearing impairment	0.53
	Behavioural impairment	0.47
	Chewing and Swallowing difficulties	0.44

SUPPLEMENTARY TABLE 1: Weighted severity scores of symptoms investigated by the EURO-PRO Questionnaire within each neuromuscular disease group		
**Disease**	**Symptom**	**Weighted symptoms severity scores**
	Urinary dysfunctions	0.44
	Dizziness	0.42
	Sensory loss, hyperesthesia or allodynia	0.23
	Speech problems	0.21
I do not know the name of my disease	Muscular Fatigue	2.00
	Muscle Weakness	1.88
	Impaired physical function/activity	1.88
	Coordination and/or balance problems	1.75
	Muscle Stiffness	1.62
	Involuntary muscle contractions	1.62
	Muscle Pain	1.50
	Vision impairment	1.50
	Gastrointestinal dysfunction	1.50
	Mental health issues	1.25
	Behavioural impairment	1.25
	Tingling	1.25
	Urinary dysfunctions	1.25
	Mental Fatigue	1.12
	Neuropathic pain	1.12
	Headache	1.12
	Cognitive impairment	1.12
	Sleep disturbance	1.12
	Hearing impairment	1.00
	Dizziness	1.00
	Autonomic symptoms	1.00
	Joint pain and/or swollen joints	0.88
	Symptoms suggestive of cardiac impairment	0.88
	Chewing and Swallowing difficulties	0.88
	Sexual dysfunction	0.88
	Sensory loss, hyperesthesia or allodynia	0.88
	Breathing difficulties	0.75
	Speech problems	0.62
Idiopathic Inflammatory Myopathies	Muscular Fatigue	2.00
	Muscle Weakness	1.97
	Impaired physical function/activity	1.90
	Coordination and/or balance problems	1.48
	Muscle Stiffness	1.39
	Mental Fatigue	1.35
	Sleep disturbance	1.32
	Muscle Pain	1.29
	Neuropathic pain	1.23

SUPPLEMENTARY TABLE 1: Weighted severity scores of symptoms investigated by the EURO-PRO Questionnaire within each neuromuscular disease group

Disease	Symptom	Weighted symptoms severity scores
	Sexual dysfunction	1.16
	Joint pain and/or swollen joints	1.00
	Chewing and Swallowing difficulties	1.00
	Involuntary muscle contractions	0.97
	Gastrointestinal dysfunction	0.94
	Autonomic symptoms	0.94
	Mental health issues	0.87
	Cognitive impairment	0.81
	Sensory loss, hyperesthesia or allodynia	0.81
	Symptoms suggestive of cardiac impairment	0.74
	Tingling	0.71
	Urinary dysfunctions	0.65
	Behavioural impairment	0.55
	Headache	0.52
	Breathing difficulties	0.48
	Hearing impairment	0.45
	Vision impairment	0.39
	Dizziness	0.35
	Speech problems	0.32
Idiopathic Neuropathies	Tingling	2.10
	Muscular Fatigue	2.00
	Muscle Weakness	2.00
	Muscle Stiffness	1.80
	Neuropathic pain	1.80
	Muscle Pain	1.70
	Mental Fatigue	1.60
	Coordination and/or balance problems	1.60
	Sleep disturbance	1.60
	Cognitive impairment	1.50
	Sensory loss, hyperesthesia or allodynia	1.20
	Impaired physical function/activity	1.10
	Joint pain and/or swollen joints	1.10
	Sexual dysfunction	1.00
	Gastrointestinal dysfunction	1.00
	Mental health issues	0.80
	Behavioural impairment	0.80
	Urinary dysfunctions	0.80
	Autonomic symptoms	0.80
	Involuntary muscle contractions	0.70
	Symptoms suggestive of cardiac impairment	0.50
	Hearing impairment	0.50

SUPPLEMENTARY TABLE 1: Weighted severity scores of symptoms investigated by the EURO-PRO Questionnaire within each neuromuscular disease group

Disease	Symptom	Weighted symptoms severity scores
	Dizziness	0.50
	Vision impairment	0.40
	Chewing and Swallowing difficulties	0.20
	Speech problems	0.20
	Breathing difficulties	0.10
	Headache	0.10
Inflammatory and Dysimmune Neuropathies	Muscular Fatigue	2.43
	Impaired physical function/activity	2.14
	Muscle Weakness	2.00
	Muscle Stiffness	1.79
	Tingling	1.79
	Coordination and/or balance problems	1.71
	Mental Fatigue	1.64
	Muscle Pain	1.64
	Involuntary muscle contractions	1.50
	Neuropathic pain	1.50
	Gastrointestinal dysfunction	1.43
	Sensory loss, hyperesthesia or allodynia	1.43
	Sleep disturbance	1.36
	Joint pain and/or swollen joints	1.00
	Headache	1.00
	Sexual dysfunction	1.00
	Urinary dysfunctions	0.86
	Vision impairment	0.79
	Mental health issues	0.79
	Autonomic symptoms	0.79
	Hearing impairment	0.71
	Cognitive impairment	0.71
	Dizziness	0.71
	Chewing and Swallowing difficulties	0.64
	Symptoms suggestive of cardiac impairment	0.57
	Behavioural impairment	0.50
	Breathing difficulties	0.21
	Speech problems	0.14
Metabolic Myopathies	Muscular Fatigue	2.12
	Muscle Weakness	2.06
	Impaired physical function/activity	1.88
	Muscle Pain	1.65
	Muscle Stiffness	1.41
	Mental Fatigue	1.35
	Joint pain and/or swollen joints	1.35

SUPPLEMENTARY TABLE 1: Weighted severity scores of symptoms investigated by the EURO-PRO Questionnaire within each neuromuscular disease group

Disease	Symptom	Weighted symptoms severity scores
	Coordination and/or balance problems	1.29
	Gastrointestinal dysfunction	1.12
	Involuntary muscle contractions	0.88
	Sexual dysfunction	0.88
	Breathing difficulties	0.82
	Sleep disturbance	0.76
	Symptoms suggestive of cardiac impairment	0.71
	Urinary dysfunctions	0.65
	Autonomic symptoms	0.65
	Neuropathic pain	0.59
	Hearing impairment	0.59
	Chewing and Swallowing difficulties	0.53
	Tingling	0.53
	Headache	0.47
	Mental health issues	0.47
	Dizziness	0.47
	Speech problems	0.35
	Cognitive impairment	0.35
	Behavioural impairment	0.35
	Vision impairment	0.29
	Sensory loss, hyperesthesia or allodynia	0.24
Mitochondrial Diseases	Muscular Fatigue	2.18
	Muscle Weakness	1.96
	Impaired physical function/activity	1.94
	Coordination and/or balance problems	1.73
	Mental Fatigue	1.67
	Vision impairment	1.62
	Muscle Stiffness	1.60
	Muscle Pain	1.50
	Sleep disturbance	1.43
	Gastrointestinal dysfunction	1.40
	Involuntary muscle contractions	1.22
	Mental health issues	1.09
	Joint pain and/or swollen joints	1.06
	Hearing impairment	1.06
	Headache	1.06
	Neuropathic pain	1.05
	Sexual dysfunction	1.03
	Cognitive impairment	1.01
	Symptoms suggestive of cardiac impairment	0.95
	Tingling	0.94

SUPPLEMENTARY TABLE 1: Weighted severity scores of symptoms investigated by the EURO-PRO Questionnaire within each neuromuscular disease group

Disease	Symptom	Weighted symptoms severity scores
	Autonomic symptoms	0.94
	Chewing and Swallowing difficulties	0.92
	Dizziness	0.83
	Speech problems	0.81
	Urinary dysfunctions	0.79
	Sensory loss, hyperesthesia or allodynia	0.78
	Behavioural impairment	0.71
	Breathing difficulties	0.67
Myasthenia gravis	Muscular Fatigue	2.00
	Muscle Weakness	1.90
	Vision impairment	1.63
	Mental Fatigue	1.61
	Impaired physical function/activity	1.56
	Muscle Pain	1.32
	Muscle Stiffness	1.29
	Involuntary muscle contractions	1.27
	Gastrointestinal dysfunction	1.24
	Mental health issues	1.20
	Sleep disturbance	1.17
	Cognitive impairment	1.10
	Coordination and/or balance problems	1.02
	Autonomic symptoms	1.02
	Headache	1.00
	Chewing and Swallowing difficulties	0.95
	Sexual dysfunction	0.90
	Symptoms suggestive of cardiac impairment	0.88
	Joint pain and/or swollen joints	0.85
	Urinary dysfunctions	0.85
	Breathing difficulties	0.76
	Speech problems	0.76
	Behavioural impairment	0.76
	Dizziness	0.73
	Tingling	0.68
	Neuropathic pain	0.61
	Hearing impairment	0.61
	Sensory loss, hyperesthesia or allodynia	0.44
Myofibrillar Myopathies	Muscle Weakness	2.50
	Muscular Fatigue	2.33
	Impaired physical function/activity	2.33
	Coordination and/or balance problems	2.00
	Muscle Stiffness	1.83

SUPPLEMENTARY TABLE 1: Weighted severity scores of symptoms investigated by the EURO-PRO Questionnaire within each neuromuscular disease group

Disease	Symptom	Weighted symptoms severity scores
	Mental Fatigue	1.50
	Muscle Pain	1.50
	Joint pain and/or swollen joints	1.50
	Symptoms suggestive of cardiac impairment	1.33
	Neuropathic pain	1.33
	Chewing and Swallowing difficulties	1.33
	Involuntary muscle contractions	1.00
	Sexual dysfunction	1.00
	Gastrointestinal dysfunction	1.00
	Speech problems	0.83
	Sleep disturbance	0.83
	Sensory loss, hyperesthesia or allodynia	0.83
	Breathing difficulties	0.67
	Mental health issues	0.67
	Behavioural impairment	0.67
	Dizziness	0.67
	Urinary dysfunctions	0.67
	Autonomic symptoms	0.67
	Vision impairment	0.50
	Cognitive impairment	0.50
	Tingling	0.50
	Hearing impairment	0.33
	Headache	0.17
Myotonic Dystrophies	Muscle Weakness	2.22
	Muscular Fatigue	2.15
	Coordination and/or balance problems	1.96
	Impaired physical function/activity	1.78
	Muscle Stiffness	1.65
	Gastrointestinal dysfunction	1.59
	Mental Fatigue	1.52
	Sleep disturbance	1.52
	Vision impairment	1.41
	Involuntary muscle contractions	1.35
	Muscle Pain	1.24
	Joint pain and/or swollen joints	1.20
	Speech problems	1.15
	Breathing difficulties	1.11
	Chewing and Swallowing difficulties	1.09
	Urinary dysfunctions	1.02
	Sexual dysfunction	0.93
	Symptoms suggestive of cardiac impairment	0.91

Disease	Symptom	Weighted symptoms severity scores
	Cognitive impairment	0.87
	Mental health issues	0.83
	Autonomic symptoms	0.80
	Tingling	0.74
	Behavioural impairment	0.70
	Headache	0.67
	Dizziness	0.63
	Neuropathic pain	0.59
	Hearing impairment	0.46
	Sensory loss, hyperesthesia or allodynia	0.39
Neuropathies associated with haematological disease and monoclonal gammopathy (MGUS, POEMS, ETC)	Joint pain and/or swollen joints	2.25
	Autonomic symptoms	2.25
	Muscular Fatigue	2.00
	Mental Fatigue	2.00
	Muscle Weakness	2.00
	Muscle Pain	2.00
	Neuropathic pain	2.00
	Muscle Stiffness	1.75
	Coordination and/or balance problems	1.75
	Gastrointestinal dysfunction	1.75
	Impaired physical function/activity	1.50
	Symptoms suggestive of cardiac impairment	1.50
	Involuntary muscle contractions	1.50
	Tingling	1.50
	Sexual dysfunction	1.50
	Sensory loss, hyperesthesia or allodynia	1.50
	Vision impairment	1.25
	Urinary dysfunctions	1.25
	Hearing impairment	1.00
	Cognitive impairment	1.00
	Mental health issues	0.75
	Behavioural impairment	0.75
	Sleep disturbance	0.75
	Chewing and Swallowing difficulties	0.50
	Headache	0.50
	Dizziness	0.50
	Breathing difficulties	0.25
	Speech problems	0.25
Other	Muscular Fatigue	2.03
	Muscle Weakness	1.87
	Impaired physical function/activity	1.86

SUPPLEMENTARY TABLE 1: Weighted severity scores of symptoms investigated by the EURO-PRO Questionnaire within each neuromuscular disease group

Disease	Symptom	Weighted symptoms severity scores
	Coordination and/or balance problems	1.70
	Muscle Stiffness	1.61
	Mental Fatigue	1.48
	Muscle Pain	1.48
	Joint pain and/or swollen joints	1.18
	Neuropathic pain	1.17
	Sleep disturbance	1.17
	Involuntary muscle contractions	1.14
	Gastrointestinal dysfunction	1.10
	Sexual dysfunction	1.06
	Mental health issues	1.01
	Tingling	0.98
	Urinary dysfunctions	0.91
	Chewing and Swallowing difficulties	0.85
	Cognitive impairment	0.82
	Sensory loss, hyperesthesia or allodynia	0.82
	Autonomic symptoms	0.77
	Vision impairment	0.75
	Headache	0.72
	Dizziness	0.68
	Symptoms suggestive of cardiac impairment	0.65
	Hearing impairment	0.64
	Behavioural impairment	0.64
	Breathing difficulties	0.58
	Speech problems	0.51
Other Muscular Dystrophies (excluding Duchenne, Becker, FSHD, myotonic dystrophies)	Impaired physical function/activity	2.37
	Muscle Weakness	2.26
	Coordination and/or balance problems	2.12
	Muscular Fatigue	2.05
	Joint pain and/or swollen joints	1.35
	Muscle Stiffness	1.34
	Muscle Pain	1.29
	Mental Fatigue	1.17
	Breathing difficulties	1.00
	Sleep disturbance	0.95
	Gastrointestinal dysfunction	0.85
	Involuntary muscle contractions	0.82
	Sexual dysfunction	0.82
	Symptoms suggestive of cardiac impairment	0.75
	Mental health issues	0.75
	Chewing and Swallowing difficulties	0.71

SUPPLEMENTARY TABLE 1: Weighted severity scores of symptoms investigated by the EURO-PRO Questionnaire within each neuromuscular disease group		
**Disease**	**Symptom**	**Weighted symptoms severity scores**
	Neuropathic pain	0.69
	Vision impairment	0.65
	Autonomic symptoms	0.65
	Tingling	0.63
	Headache	0.58
	Urinary dysfunctions	0.58
	Hearing impairment	0.40
	Behavioural impairment	0.40
	Dizziness	0.37
	Cognitive impairment	0.35
	Sensory loss, hyperesthesia or allodynia	0.35
	Speech problems	0.32
Skeletal Muscle Channelopathies	Muscular Fatigue	3.00
	Mental Fatigue	3.00
	Impaired physical function/activity	3.00
	Hearing impairment	3.00
	Cognitive impairment	3.00
	Mental health issues	3.00
	Sleep disturbance	3.00
	Autonomic symptoms	3.00
	Muscle Weakness	2.00
	Muscle Stiffness	2.00
	Coordination and/or balance problems	2.00
	Muscle Pain	2.00
	Joint pain and/or swollen joints	2.00
	Tingling	2.00
	Gastrointestinal dysfunction	2.00
	Urinary dysfunctions	2.00
	Sensory loss, hyperesthesia or allodynia	2.00
	Involuntary muscle contractions	1.00
	Chewing and Swallowing difficulties	1.00
	Dizziness	1.00
	Sexual dysfunction	1.00
	Symptoms suggestive of cardiac impairment	0.00
	Neuropathic pain	0.00
	Vision impairment	0.00
	Breathing difficulties	0.00
	Speech problems	0.00
	Headache	0.00
	Behavioural impairment	0.00
Small Fibre Neuropathies	Neuropathic pain	2.61

SUPPLEMENTARY TABLE 1: Weighted severity scores of symptoms investigated by the EURO-PRO Questionnaire within each neuromuscular disease group

Disease	Symptom	Weighted symptoms severity scores
	Muscular Fatigue	2.33
	Sensory loss, hyperesthesia or allodynia	2.17
	Gastrointestinal dysfunction	2.11
	Tingling	2.06
	Autonomic symptoms	2.06
	Mental Fatigue	1.89
	Sleep disturbance	1.89
	Coordination and/or balance problems	1.83
	Cognitive impairment	1.83
	Impaired physical function/activity	1.78
	Muscle Stiffness	1.72
	Joint pain and/or swollen joints	1.61
	Vision impairment	1.56
	Urinary dysfunctions	1.56
	Muscle Pain	1.50
	Muscle Weakness	1.44
	Involuntary muscle contractions	1.39
	Symptoms suggestive of cardiac impairment	1.28
	Dizziness	1.22
	Sexual dysfunction	1.22
	Behavioural impairment	1.17
	Mental health issues	1.11
	Headache	1.06
	Hearing impairment	0.78
	Chewing and Swallowing difficulties	0.72
	Speech problems	0.72
	Breathing difficulties	0.39
Spinal Muscular Atrophy (SMA)	Impaired physical function/activity	2.46
	Muscle Weakness	2.23
	Muscular Fatigue	1.83
	Coordination and/or balance problems	1.77
	Breathing difficulties	1.31
	Muscle Stiffness	1.07
	Joint pain and/or swollen joints	1.01
	Mental Fatigue	1.00
	Chewing and Swallowing difficulties	1.00
	Sleep disturbance	0.94
	Involuntary muscle contractions	0.80
	Muscle Pain	0.73
	Gastrointestinal dysfunction	0.73
	Symptoms suggestive of cardiac impairment	0.63

SUPPLEMENTARY TABLE 1: Weighted severity scores of symptoms investigated by the EURO-PRO Questionnaire within each neuromuscular disease group		
**Disease**	**Symptom**	**Weighted symptoms severity scores**
	Autonomic symptoms	0.61
	Mental health issues	0.59
	Headache	0.50
	Neuropathic pain	0.46
	Tingling	0.41
	Speech problems	0.39
	Sensory loss, hyperesthesia or allodynia	0.33
	Cognitive impairment	0.31
	Vision impairment	0.30
	Dizziness	0.24
	Sexual dysfunction	0.24
	Urinary dysfunctions	0.23
	Behavioural impairment	0.19
	Hearing impairment	0.13
