# Supplementary material for: The most bothersome symptoms in neuromuscular diseases: the ERN EURO NMD Survey
Source: Orphanet J Rare Dis. 2025 May 8;20:221. doi: 10.1186/s13023-025-03742-z (PMC12063438; doi:10.1186/s13023-025-03742-z)
Supplement: Supplementary file 2 — Supplementary Material 2 [file 13023_2025_3742_MOESM2_ESM.pdf]

Supplementary Figures showing the patients' severity score of each symptom for each NMD

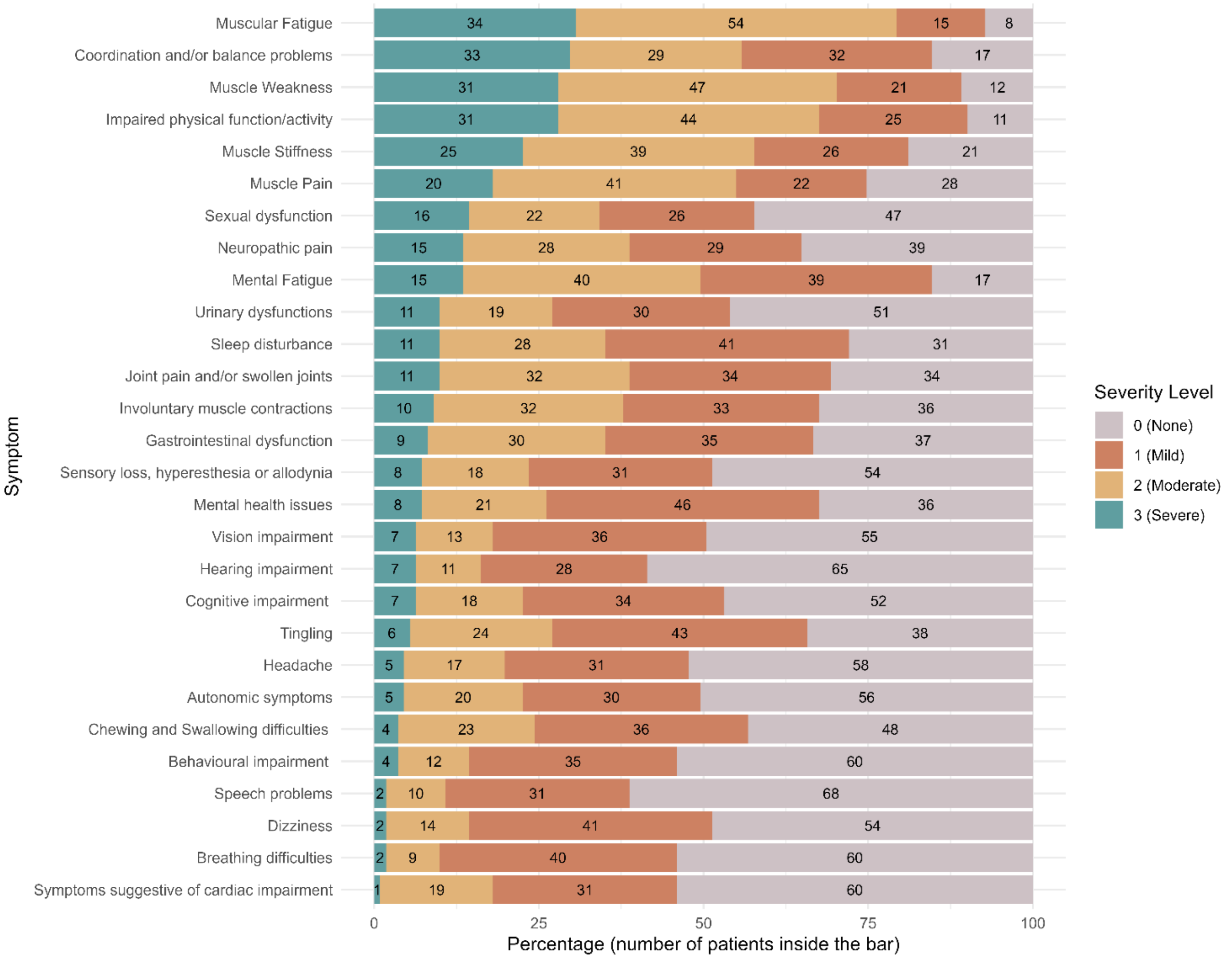

Other

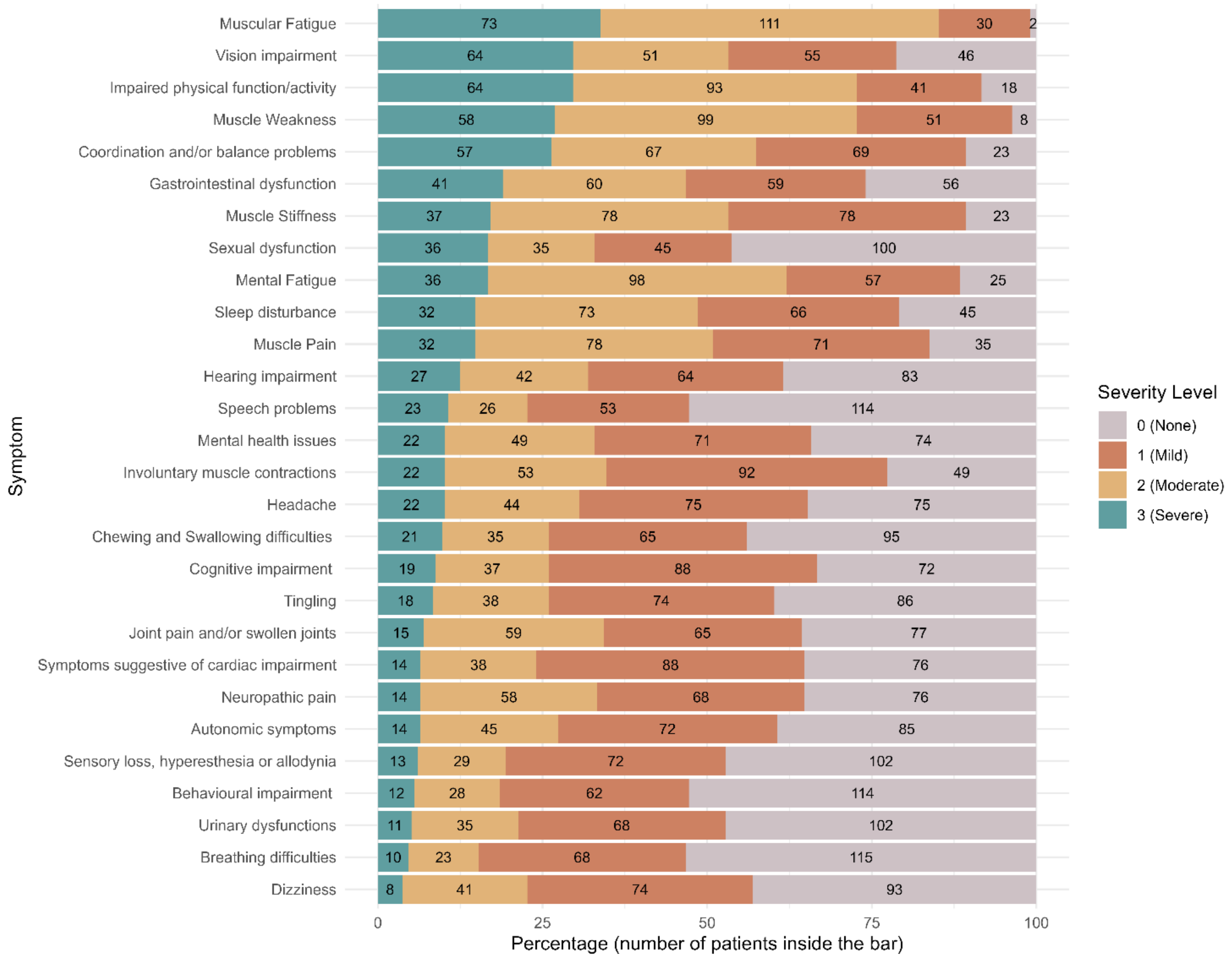

Mitochondrial Diseases

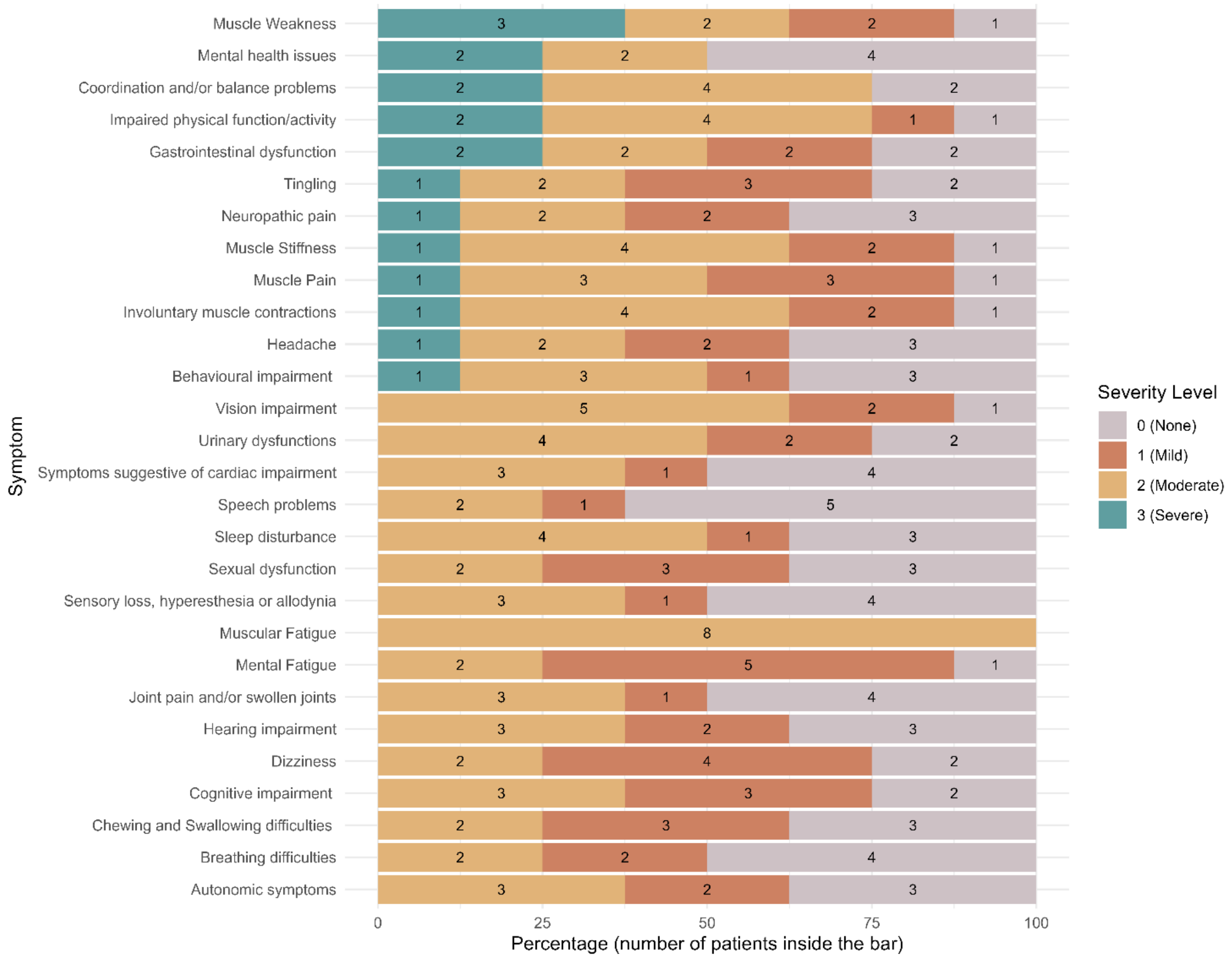

I do not know the name of my disease

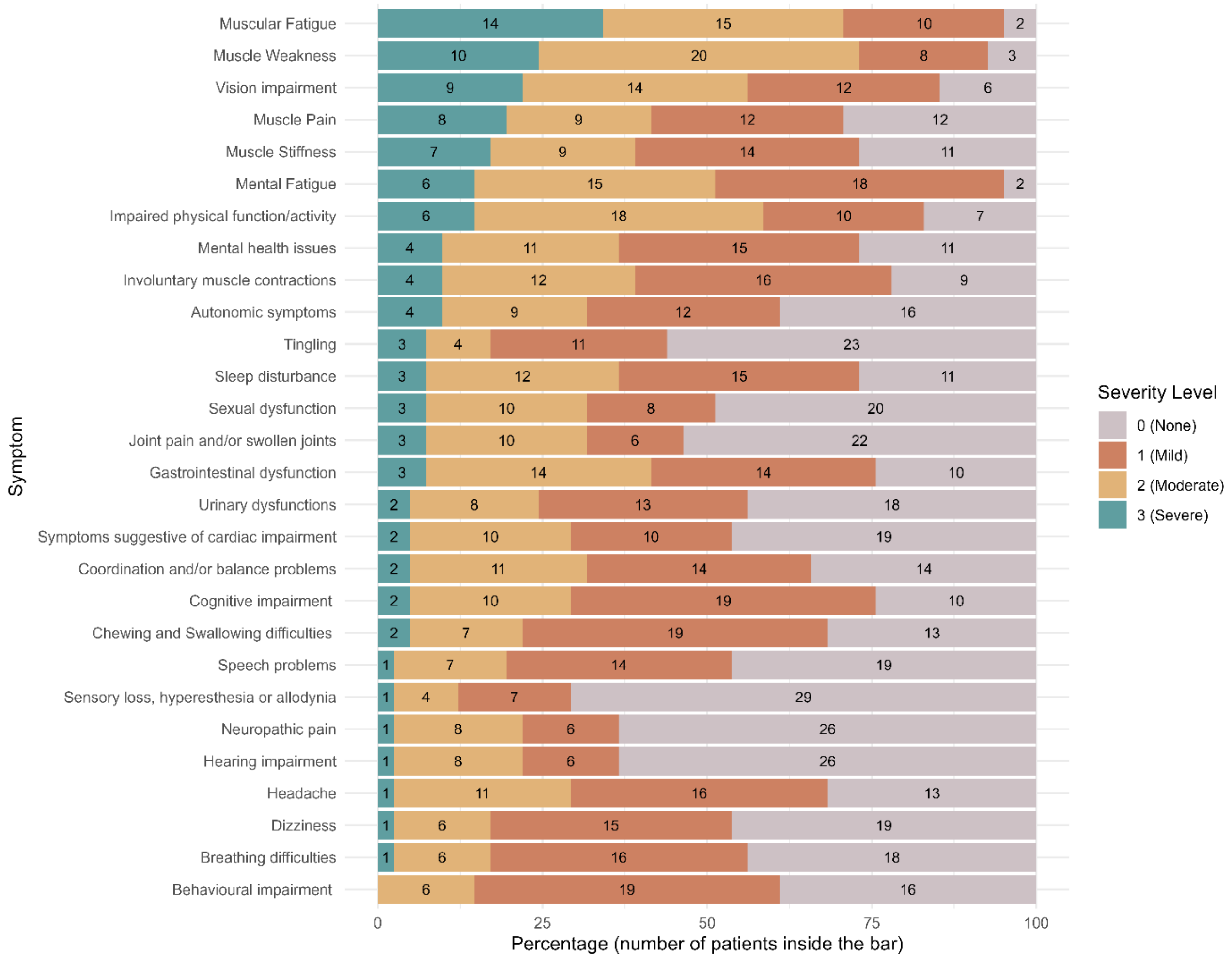

Myasthenia gravis

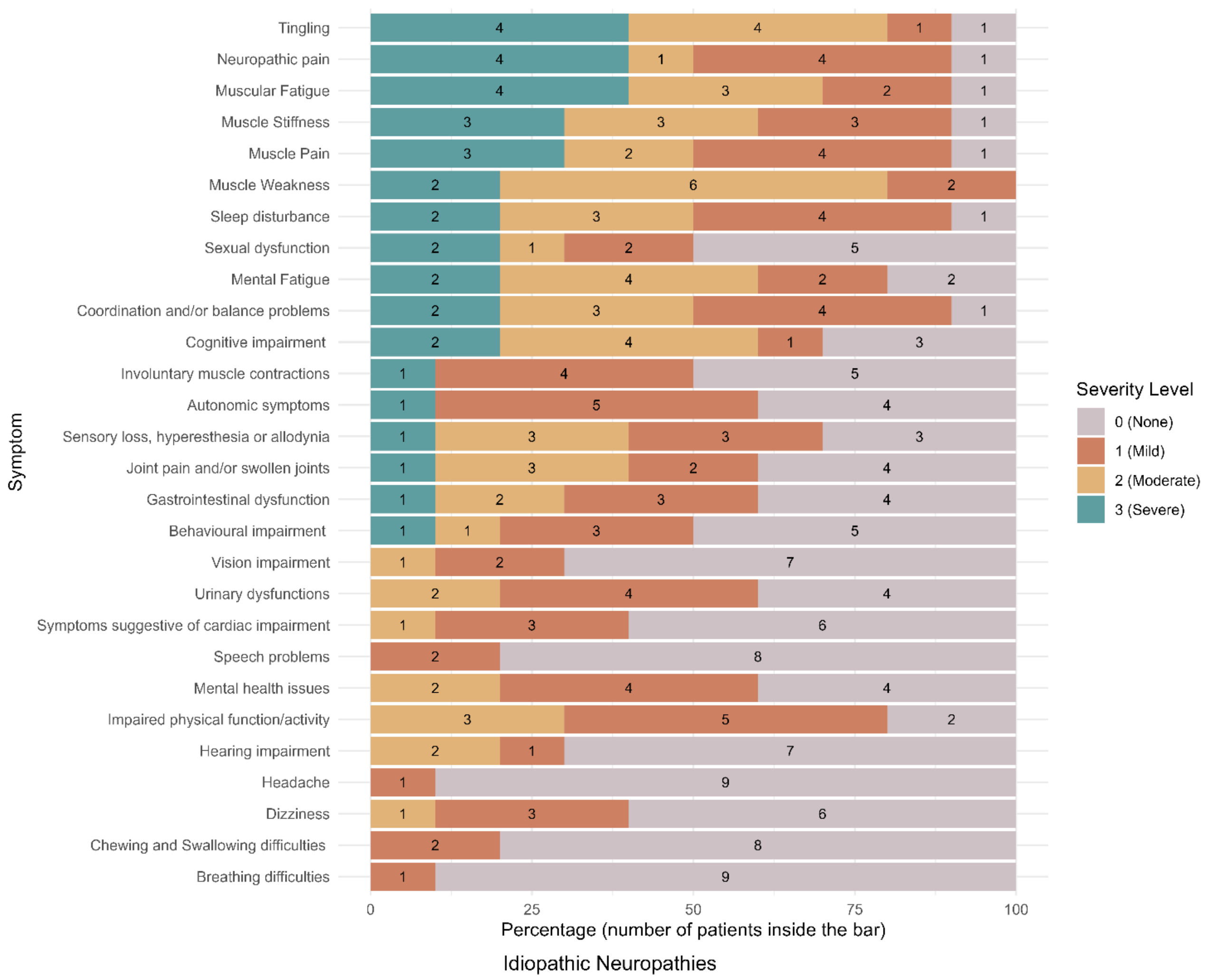

Tingling
Neuropathic pain
Muscular Fatigue
Muscle Stiffness
Muscle Pain
Muscle Weakness
Sleep disturbance
Sexual dysfunction
Mental Fatigue
Coordination and/or balance problems
Cognitive impairment
Involuntary muscle contractions
Autonomic symptoms
Sensory loss, hyperesthesia or allodynia
Joint pain and/or swollen joints
Gastrointestinal dysfunction
Behavioural impairment
Vision impairment
Urinary dysfunctions
Symptoms suggestive of cardiac impairment
Speech problems
Mental health issues
Impaired physical function/activity
Hearing impairment
Headache
Dizziness
Chewing and Swallowing difficulties
Breathing difficulties
Symptom
0
25
50
75
100
Percentage (number of patients inside the bar)
Idiopathic Neuropathies
Severity Level
0 (None)
1 (Mild)
2 (Moderate)
3 (Severe)

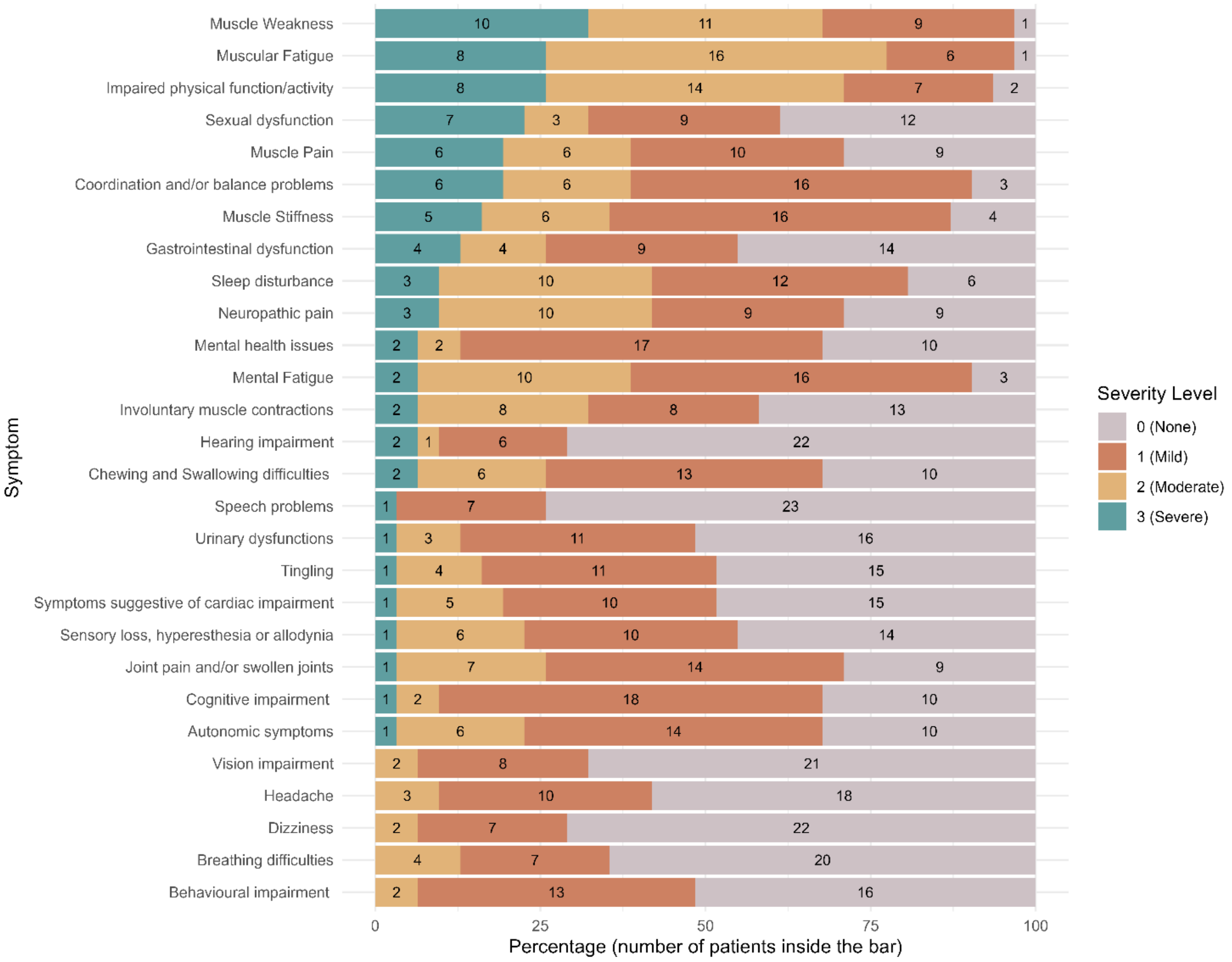

Idiopathic Inflammatory Myopathies

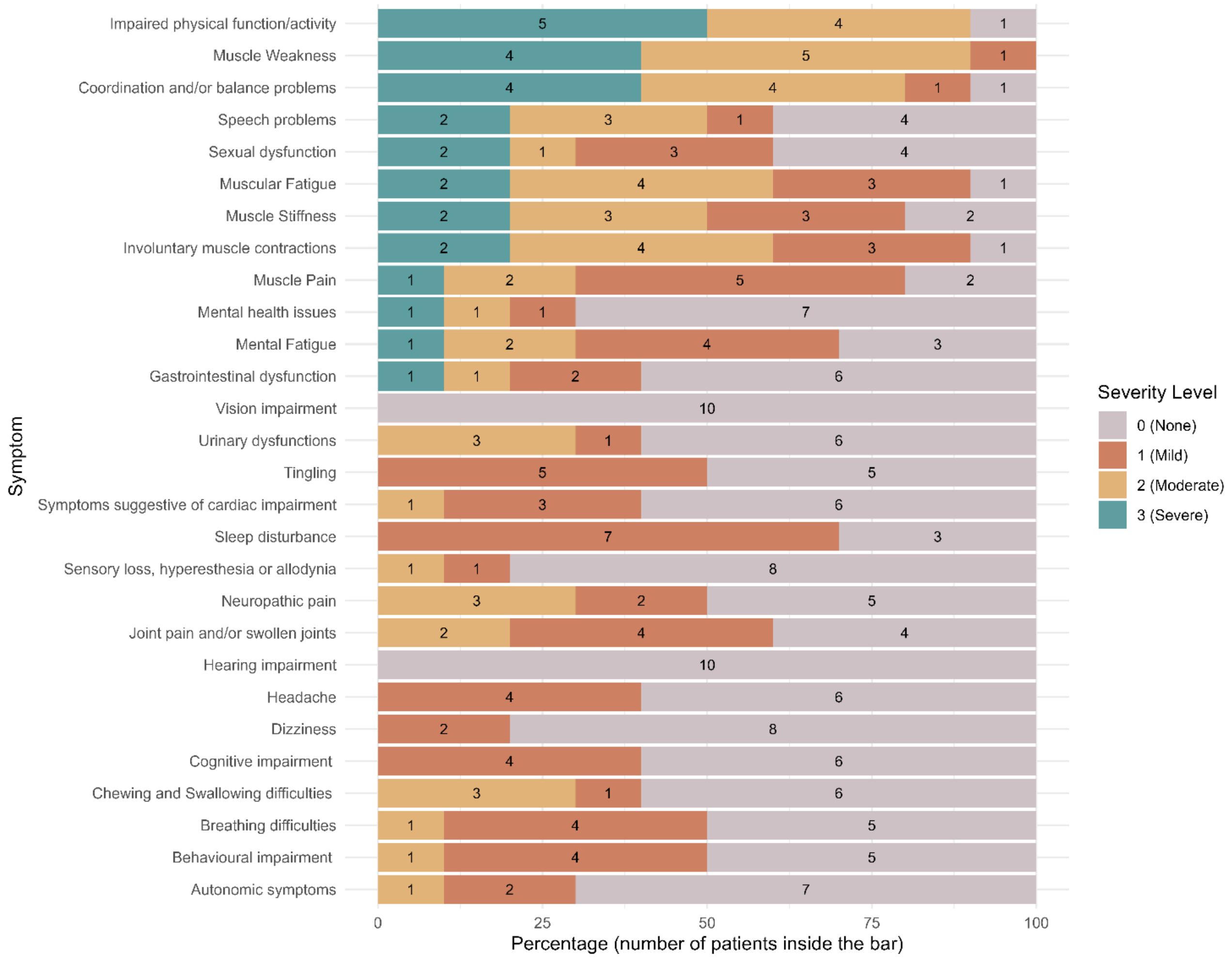

Amyotrophic Lateral Sclerosis and other motor neuron diseases (excluding SMA)

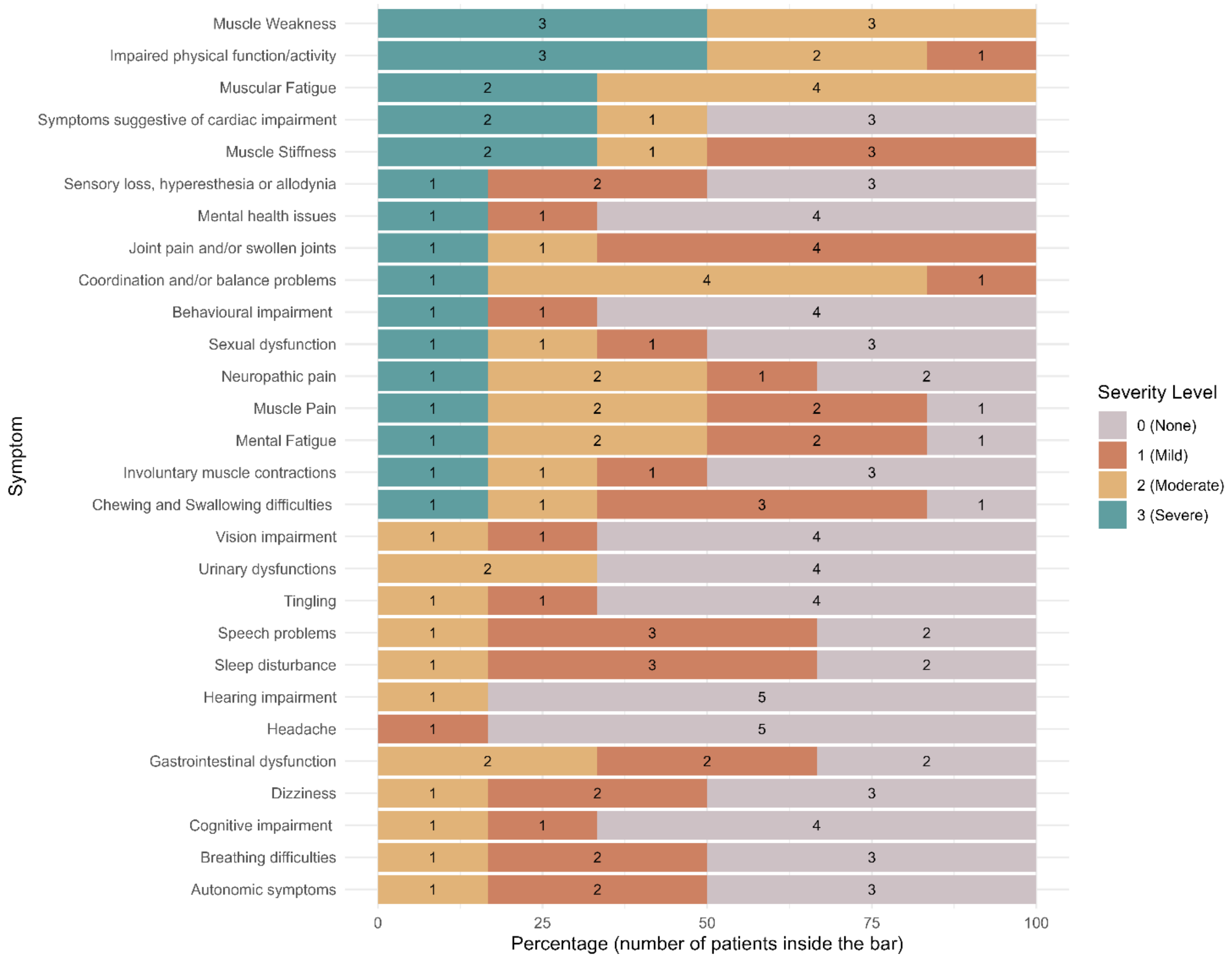

Myofibrillar Myopathies

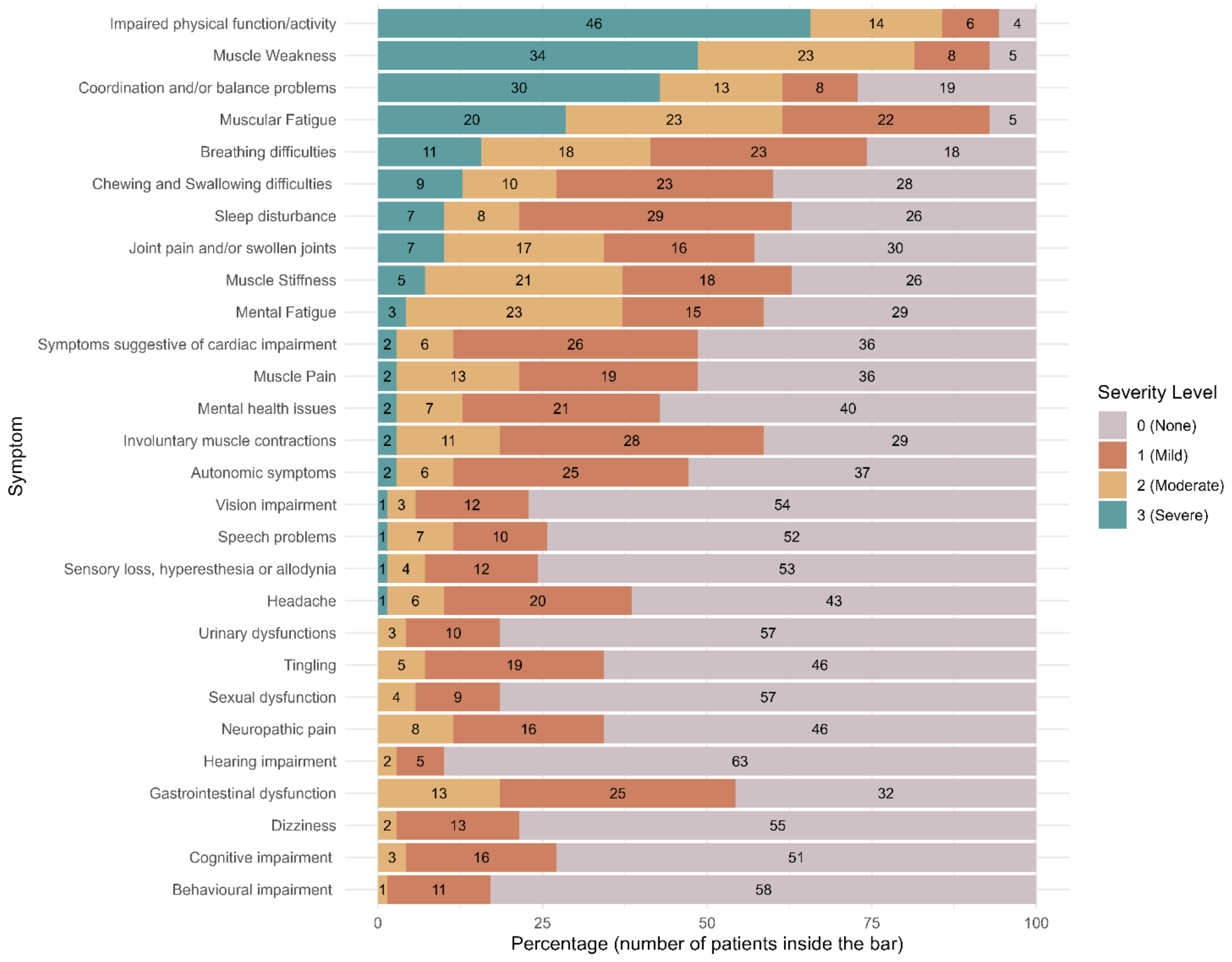

Spinal Muscular Atrophy (SMA)

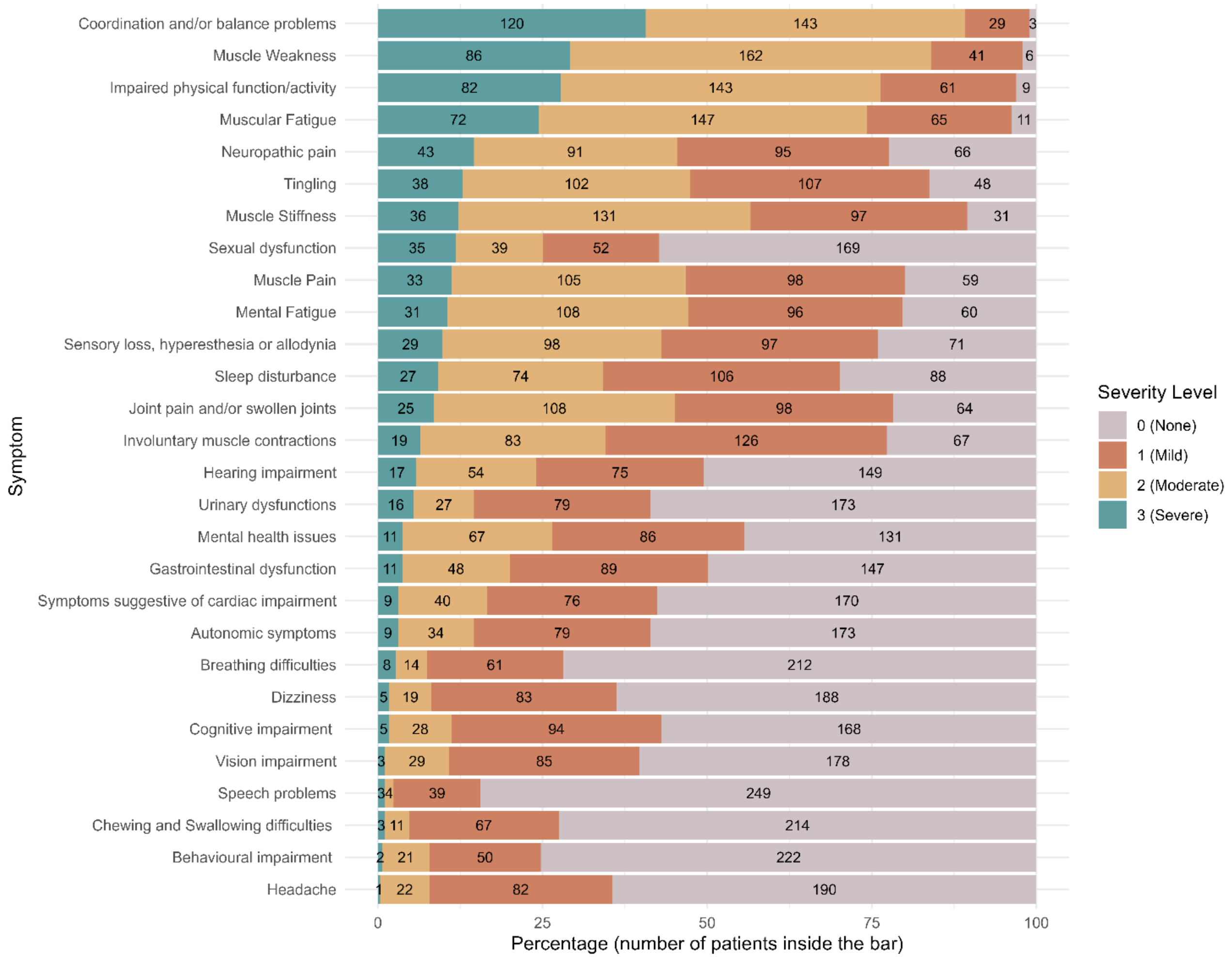

Charcot-Marie Tooth and related neuropathies (HNNP, HSAN, dHMN)

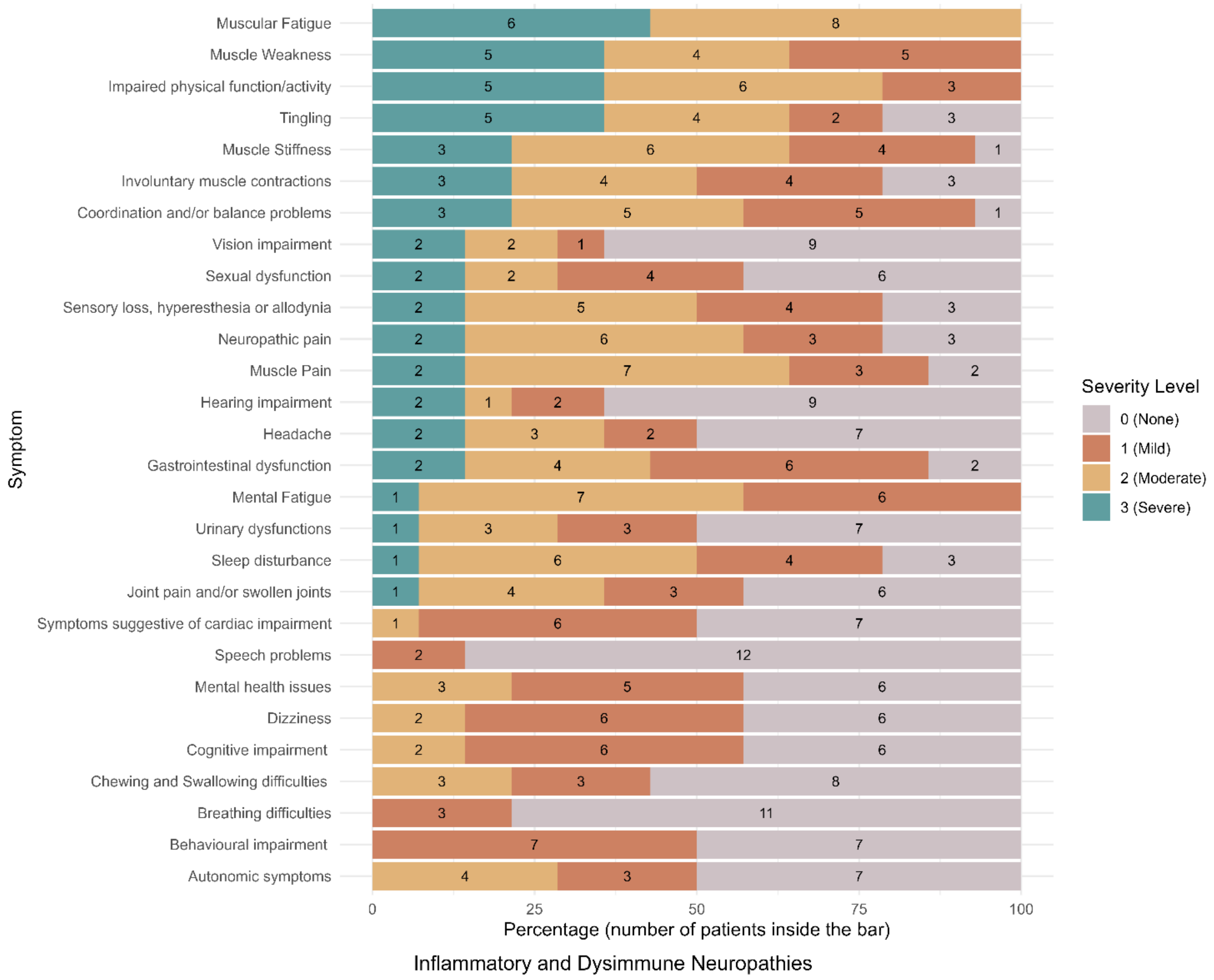
Symptom
Muscular Fatigue
Muscle Weakness
Impaired physical function/activity
Tingling
Muscle Stiffness
Involuntary muscle contractions
Coordination and/or balance problems
Vision impairment
Sexual dysfunction
Sensory loss, hyperesthesia or allodynia
Neuropathic pain
Muscle Pain
Hearing impairment
Headache
Gastrointestinal dysfunction
Mental Fatigue
Urinary dysfunctions
Sleep disturbance
Joint pain and/or swollen joints
Symptoms suggestive of cardiac impairment
Speech problems
Mental health issues
Dizziness
Cognitive impairment
Chewing and Swallowing difficulties
Breathing difficulties
Behavioural impairment
Autonomic symptoms
0
25
50
75
100
Percentage (number of patients inside the bar)
Severity Level
0 (None)
1 (Mild)
2 (Moderate)
3 (Severe)
Inflammatory and Dysimmune Neuropathies

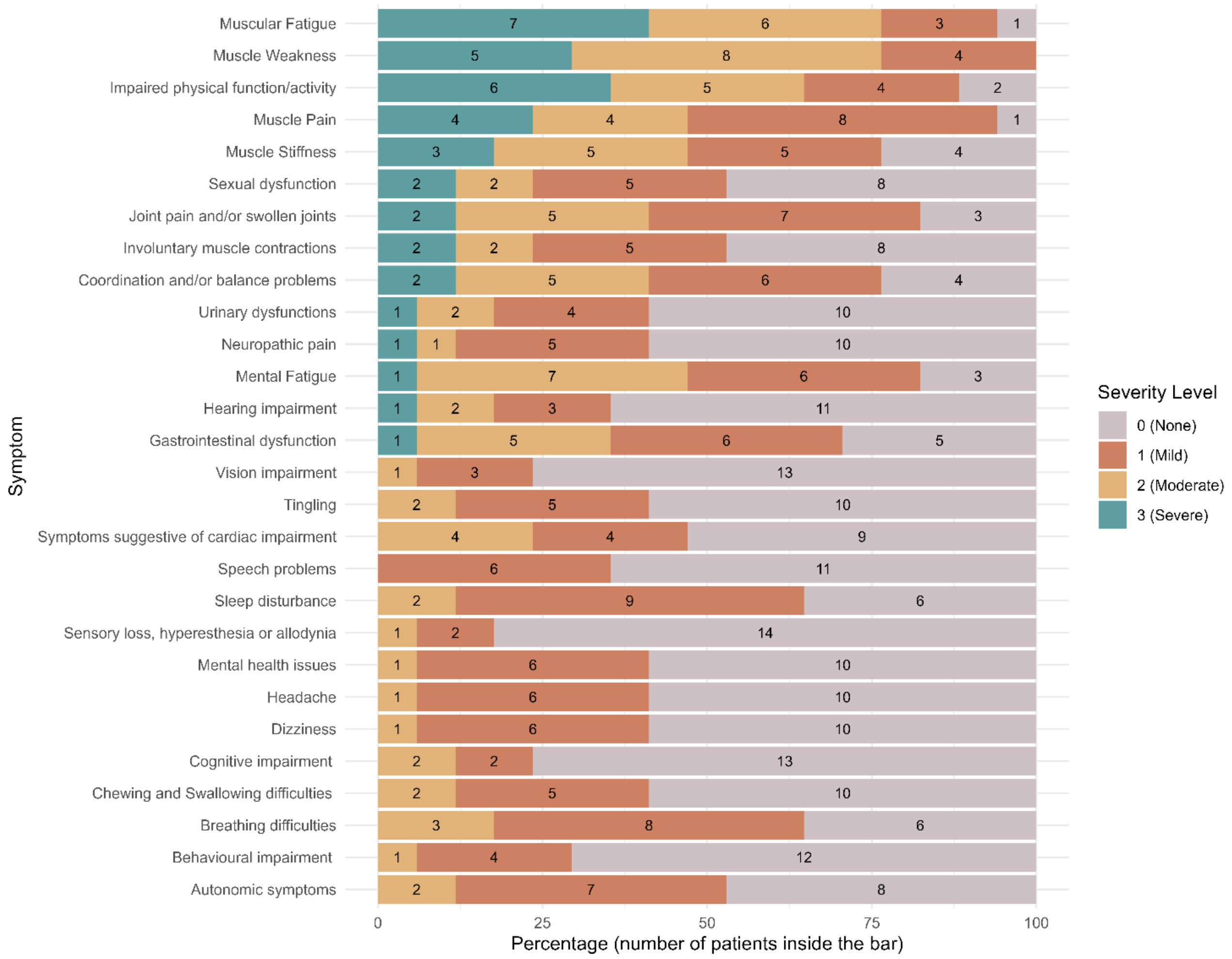

Metabolic Myopathies

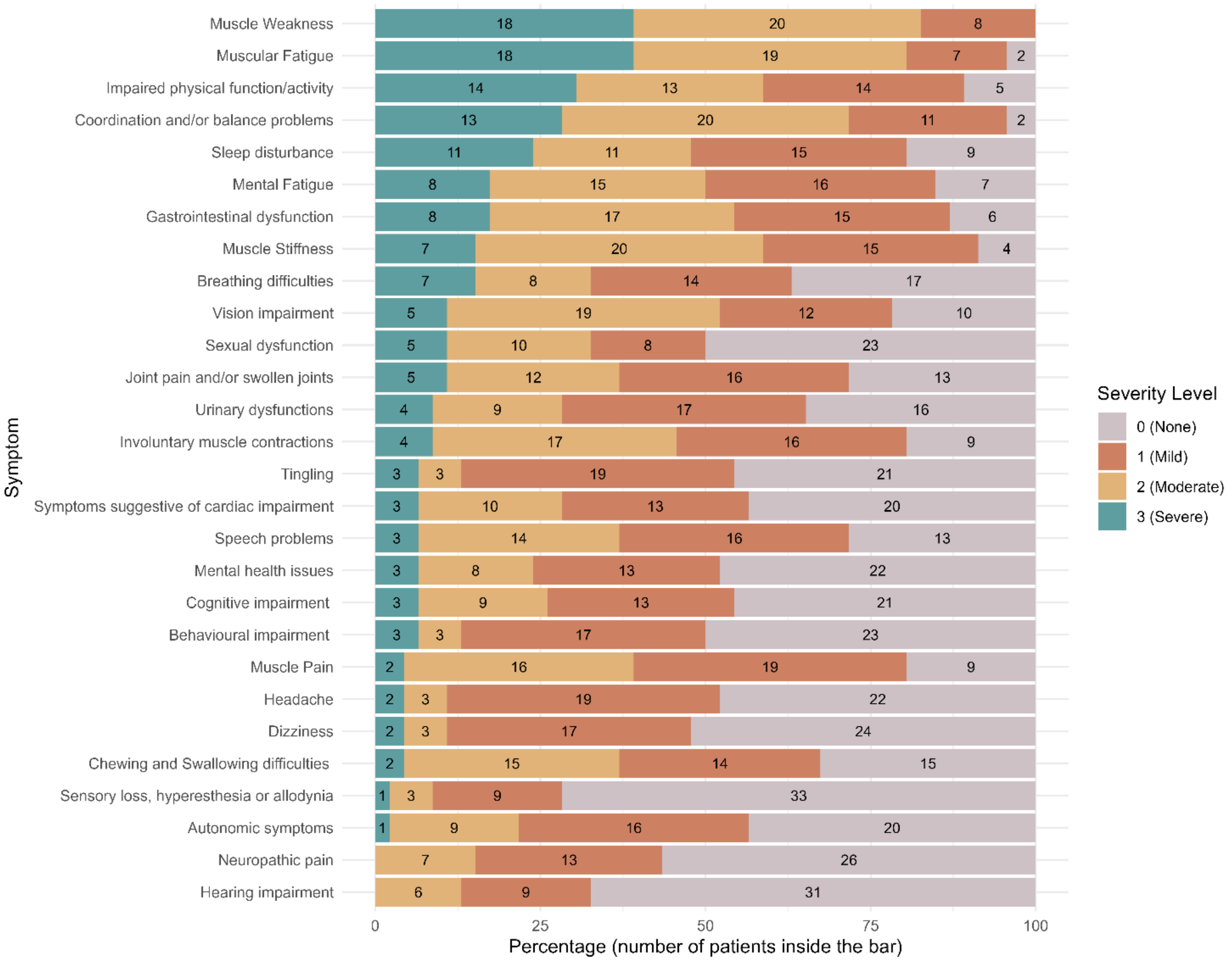

Myotonic Dystrophies

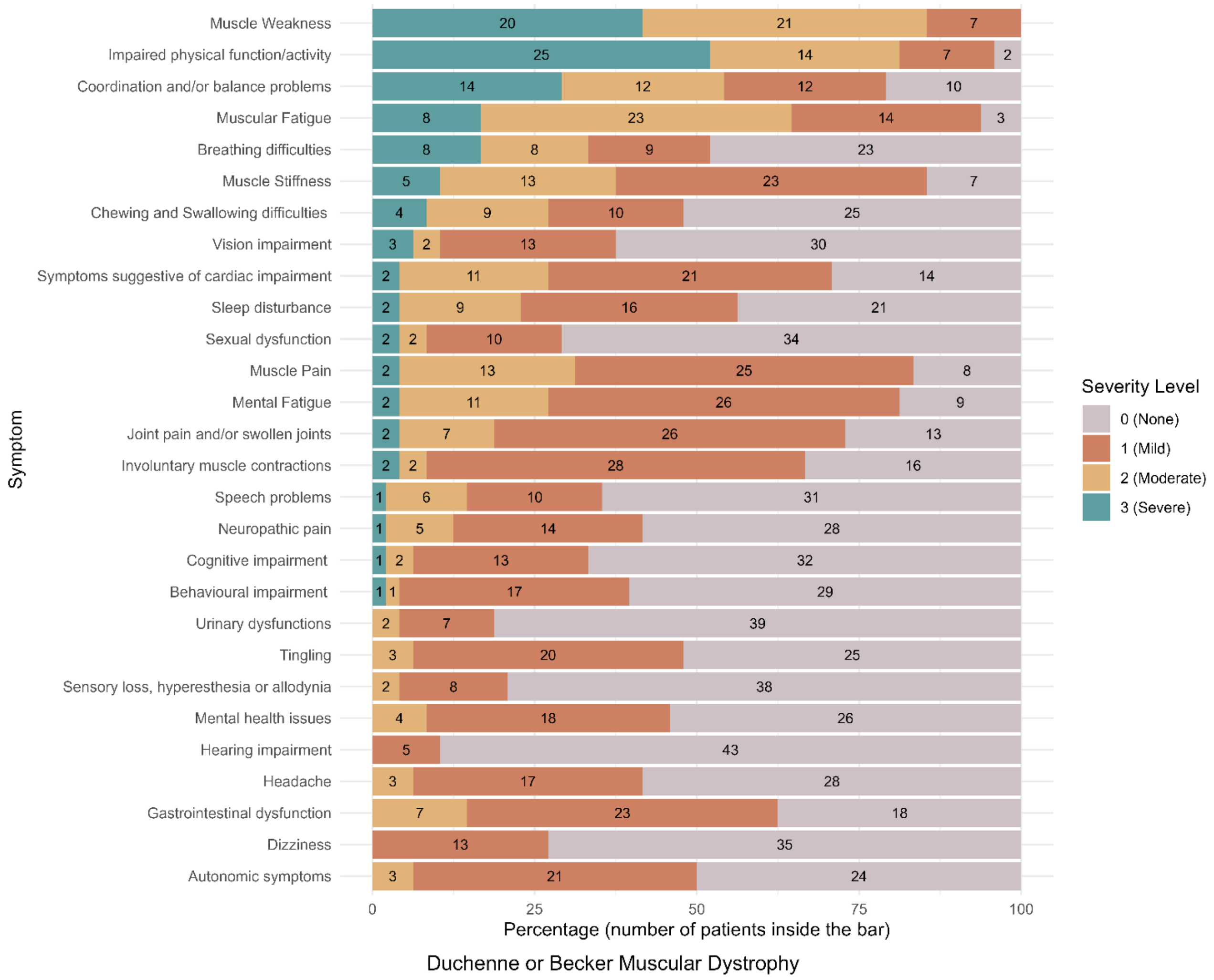
Muscle Weakness
Impaired physical function/activity
Coordination and/or balance problems
Muscular Fatigue
Breathing difficulties
Muscle Stiffness
Chewing and Swallowing difficulties
Vision impairment
Symptoms suggestive of cardiac impairment
Sleep disturbance
Sexual dysfunction
Muscle Pain
Mental Fatigue
Joint pain and/or swollen joints
Involuntary muscle contractions
Speech problems
Neuropathic pain
Cognitive impairment
Behavioural impairment
Urinary dysfunctions
Tingling
Sensory loss, hyperesthesia or allodynia
Mental health issues
Hearing impairment
Headache
Gastrointestinal dysfunction
Dizziness
Autonomic symptoms
Symptom
Percentage (number of patients inside the bar)
Severity Level
0 (None)
1 (Mild)
2 (Moderate)
3 (Severe)
Duchenne or Becker Muscular Dystrophy

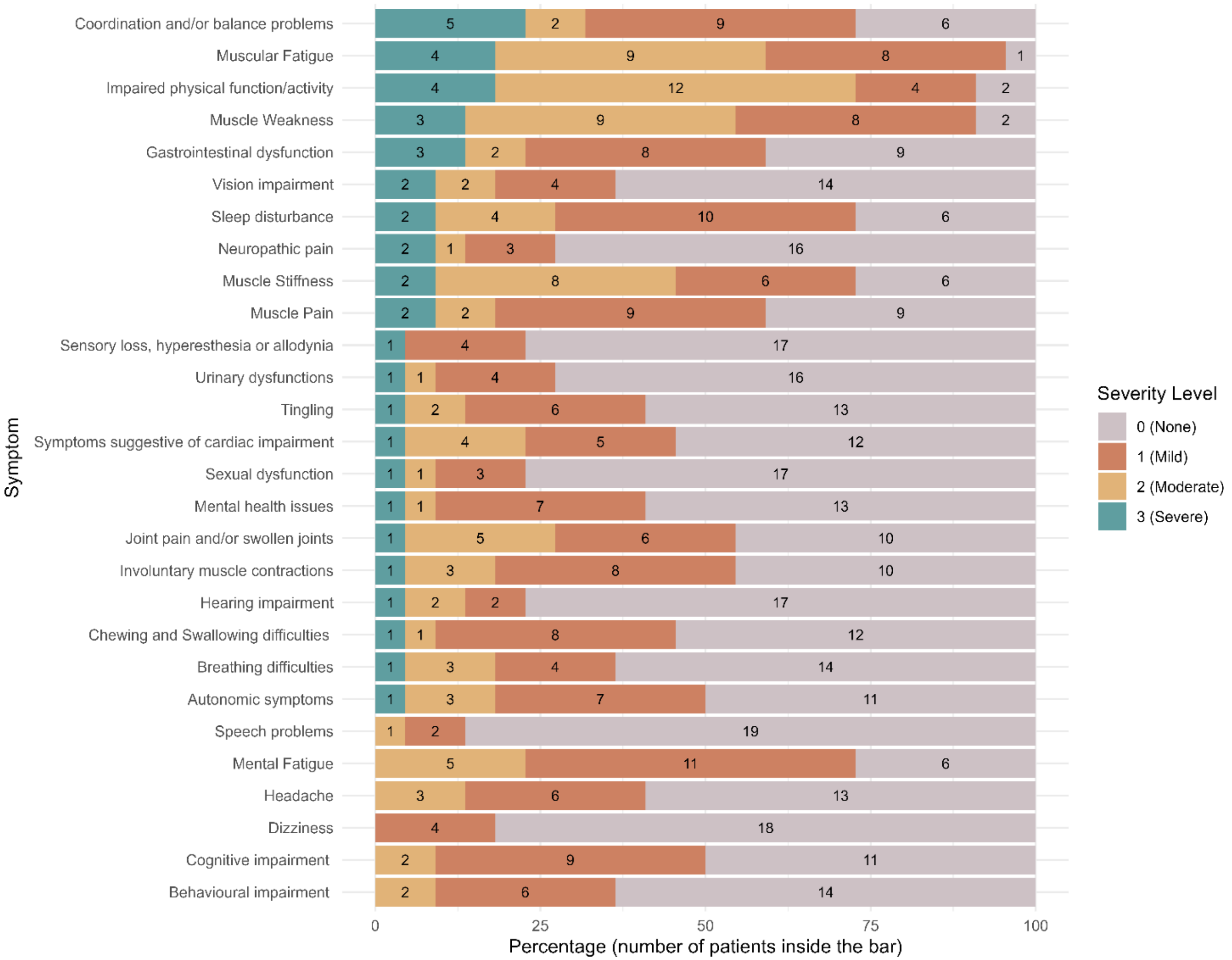

Congenital Myopathies and Congenital muscular dystrophies

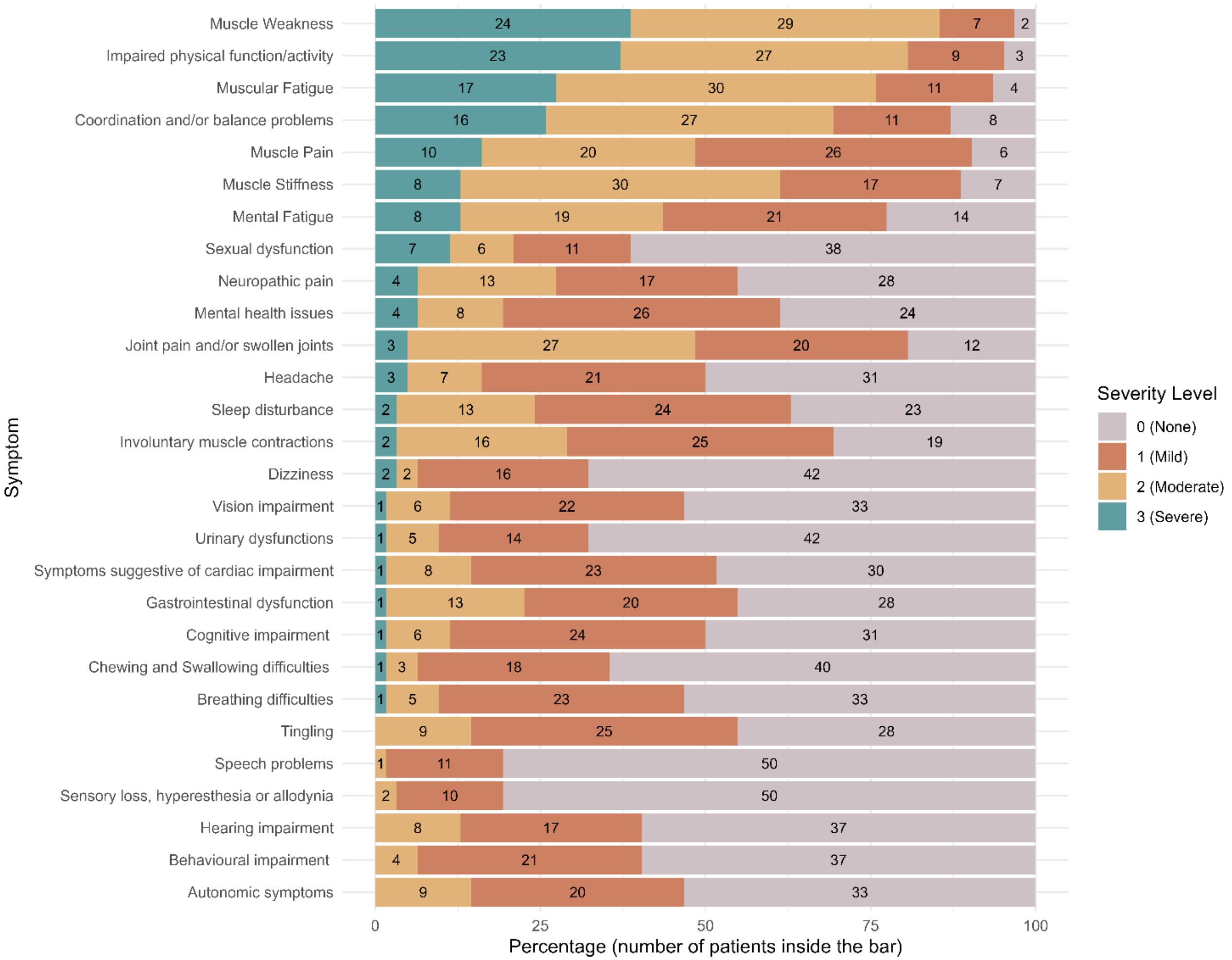

Facioscapulohumeral Muscular Dystrophy (FSHD)

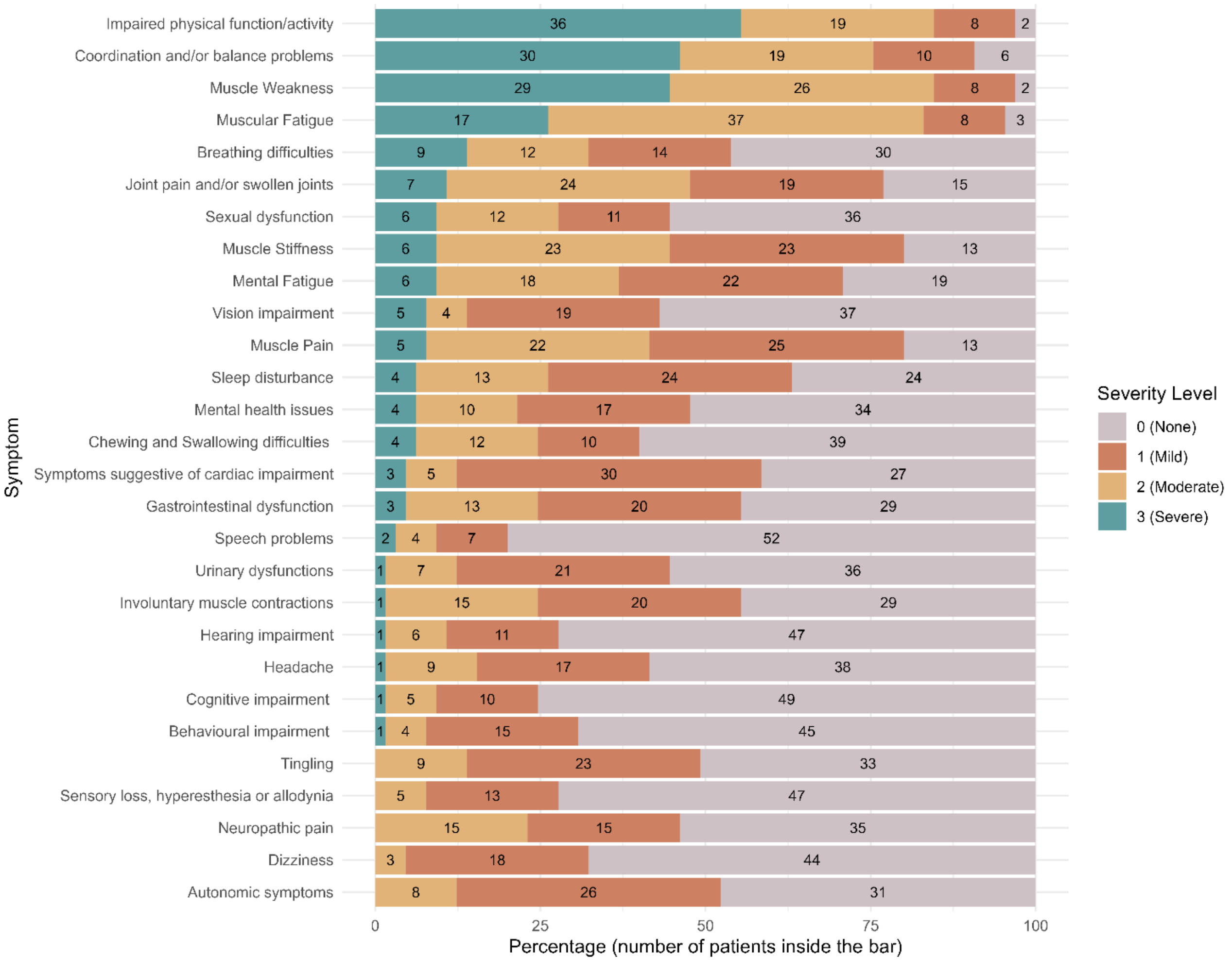

Other Muscular Dystrophies (excluding Duchenne, Becker, FSHD, myotonic dystrophies)

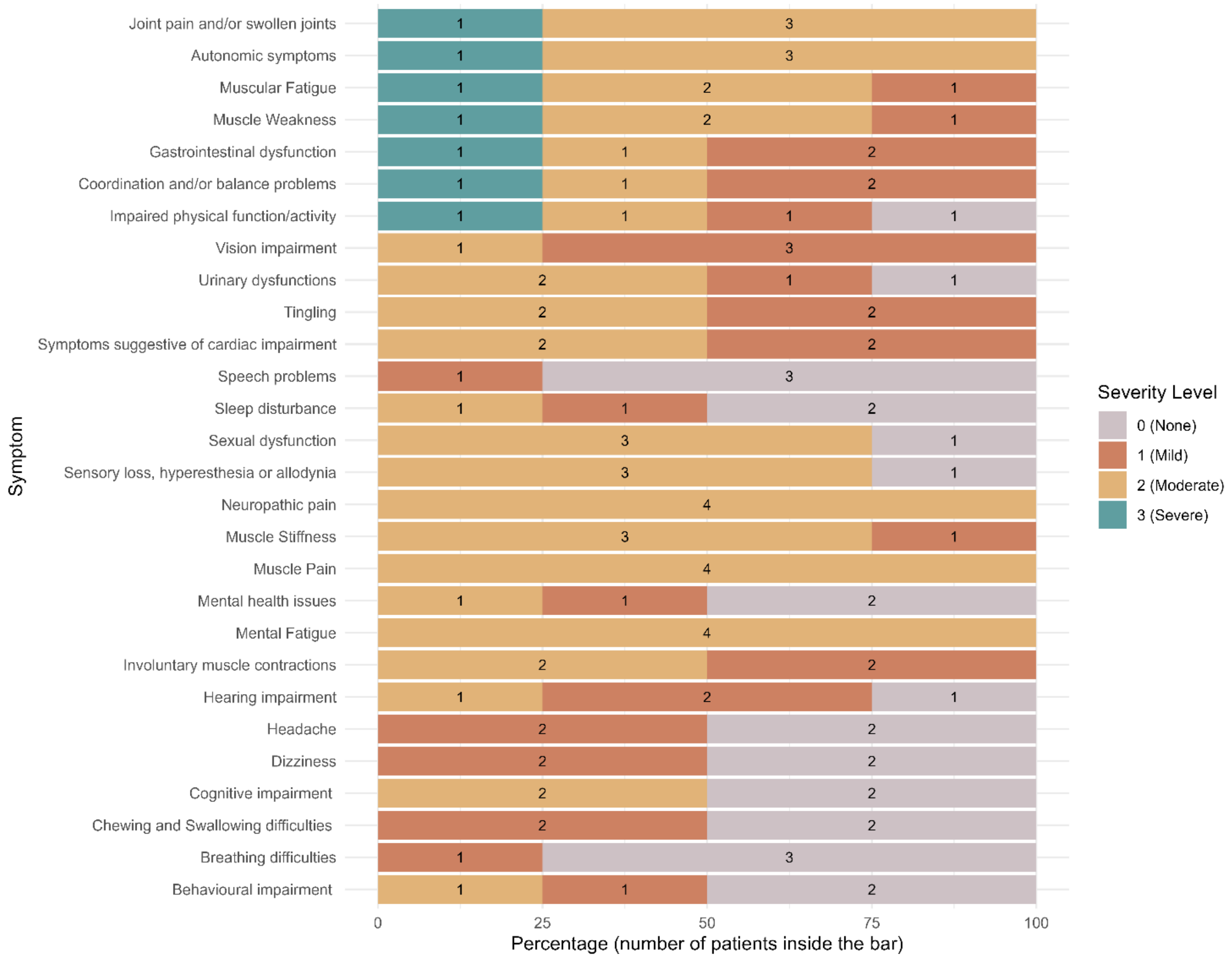

Neuropathies associated with haematological disease and monoclonal gammopathy (MGUS, POEMS, ETC)

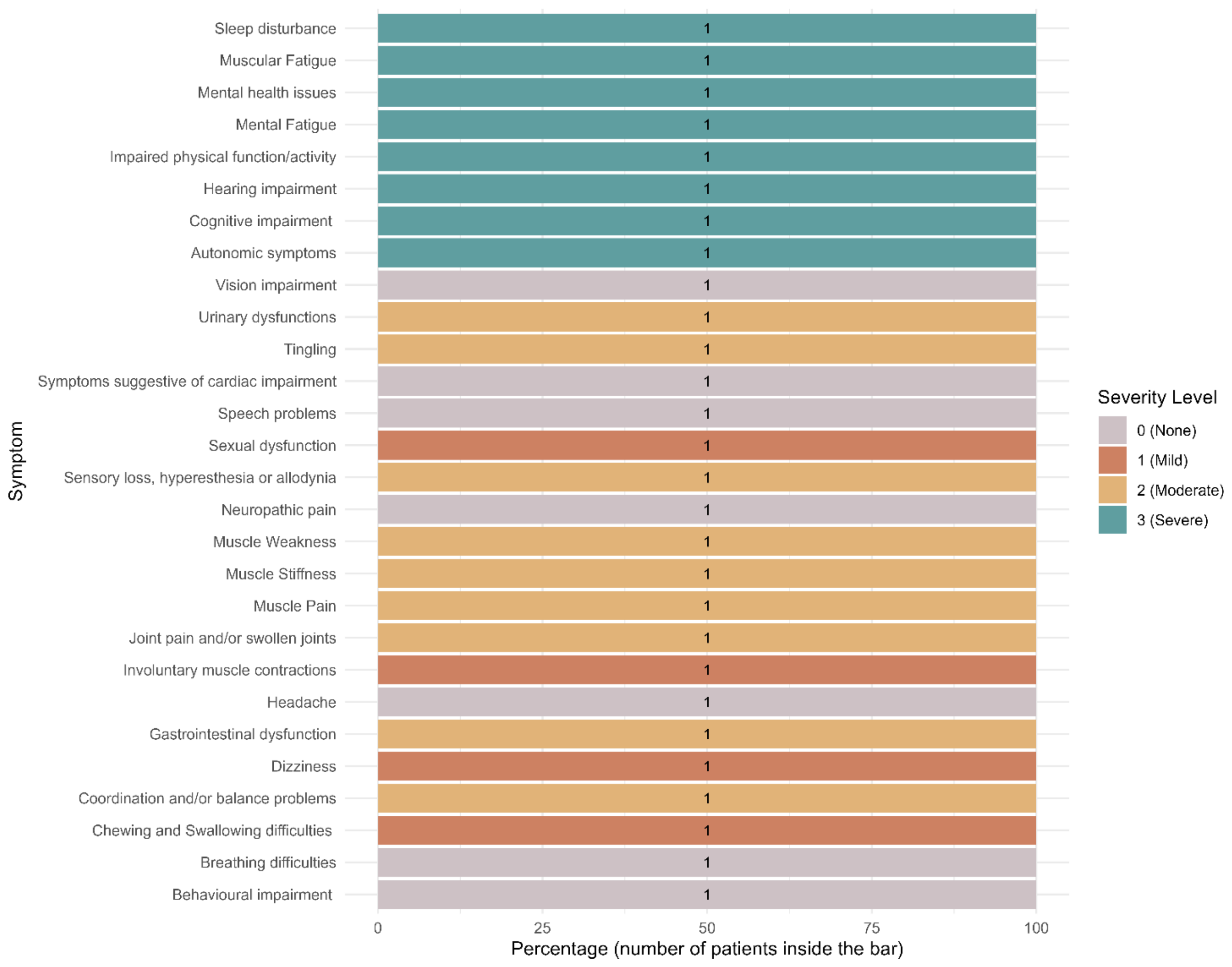

Skeletal Muscle Channelopathies

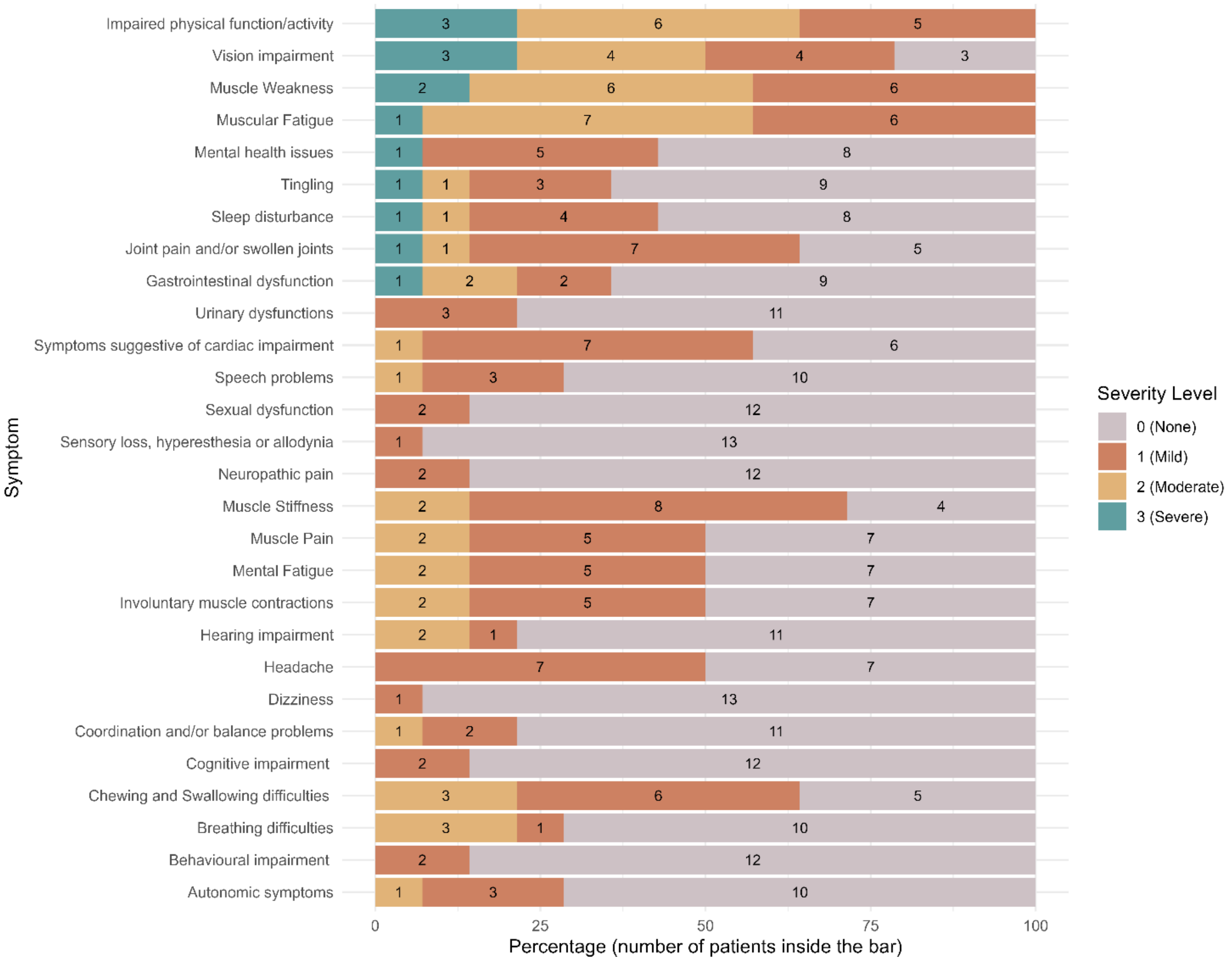

Congenital Myasthenic Syndromes

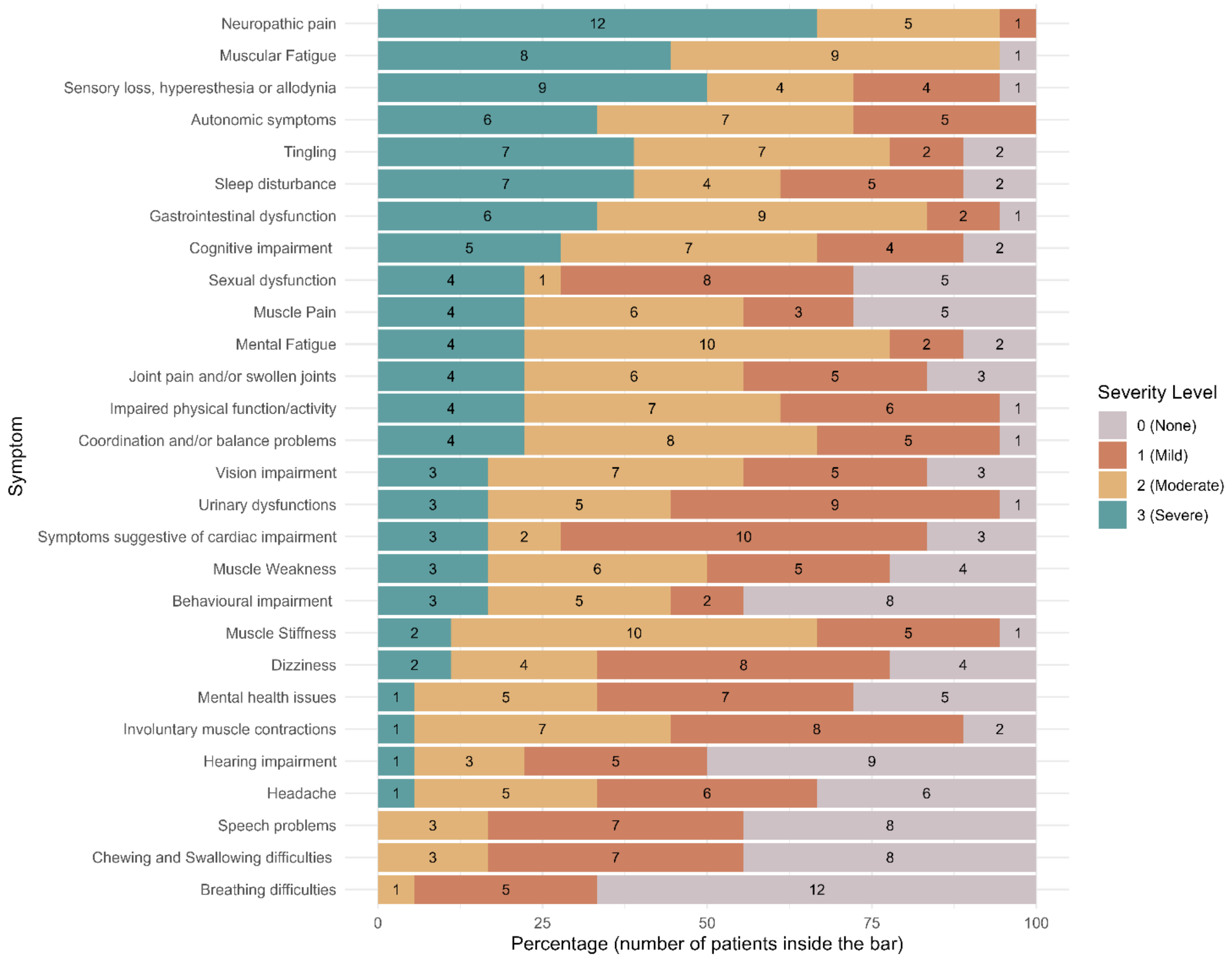

Small Fibre Neuropathies

Supplementary Figures showing differences in severity scores between age groups and sexes in the entire cohort and within each NMD group, excluding underrepresented NMD groups and the "Other NMDs" group

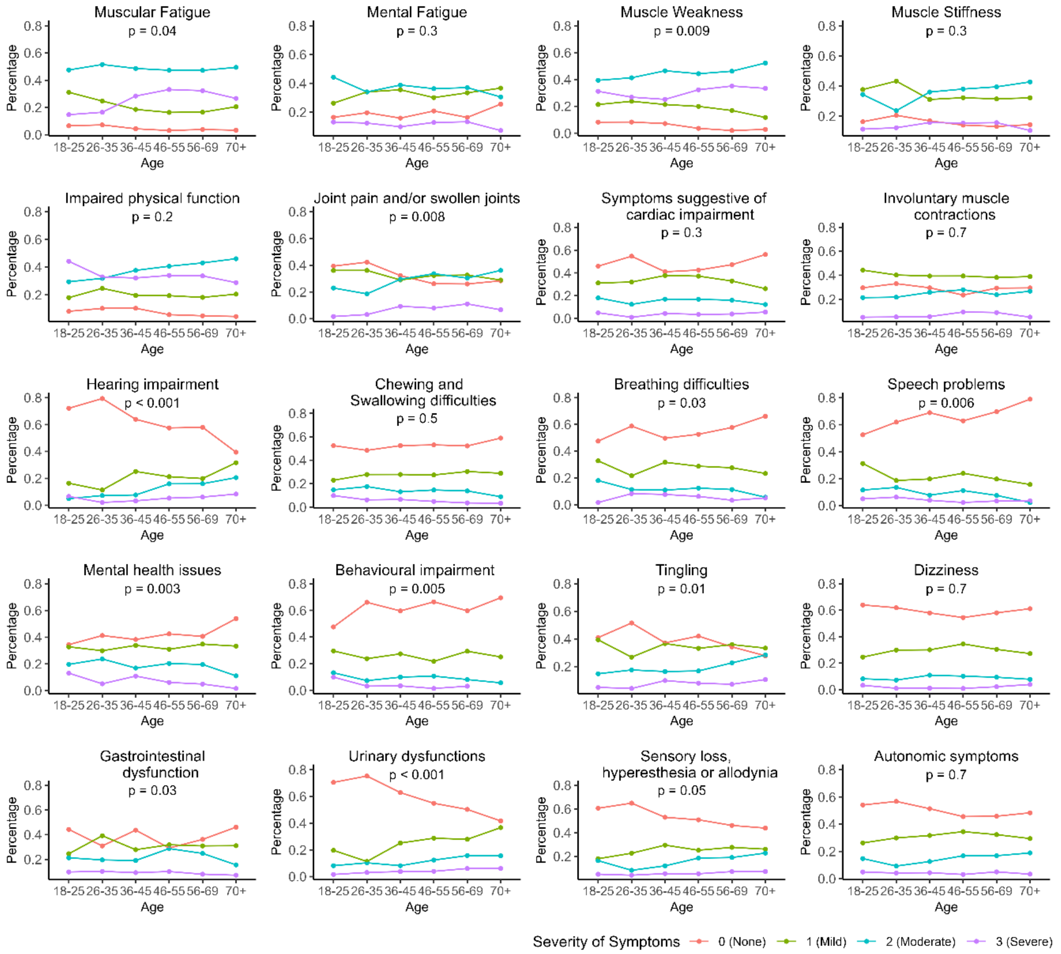

Entire cohort of NMD patients

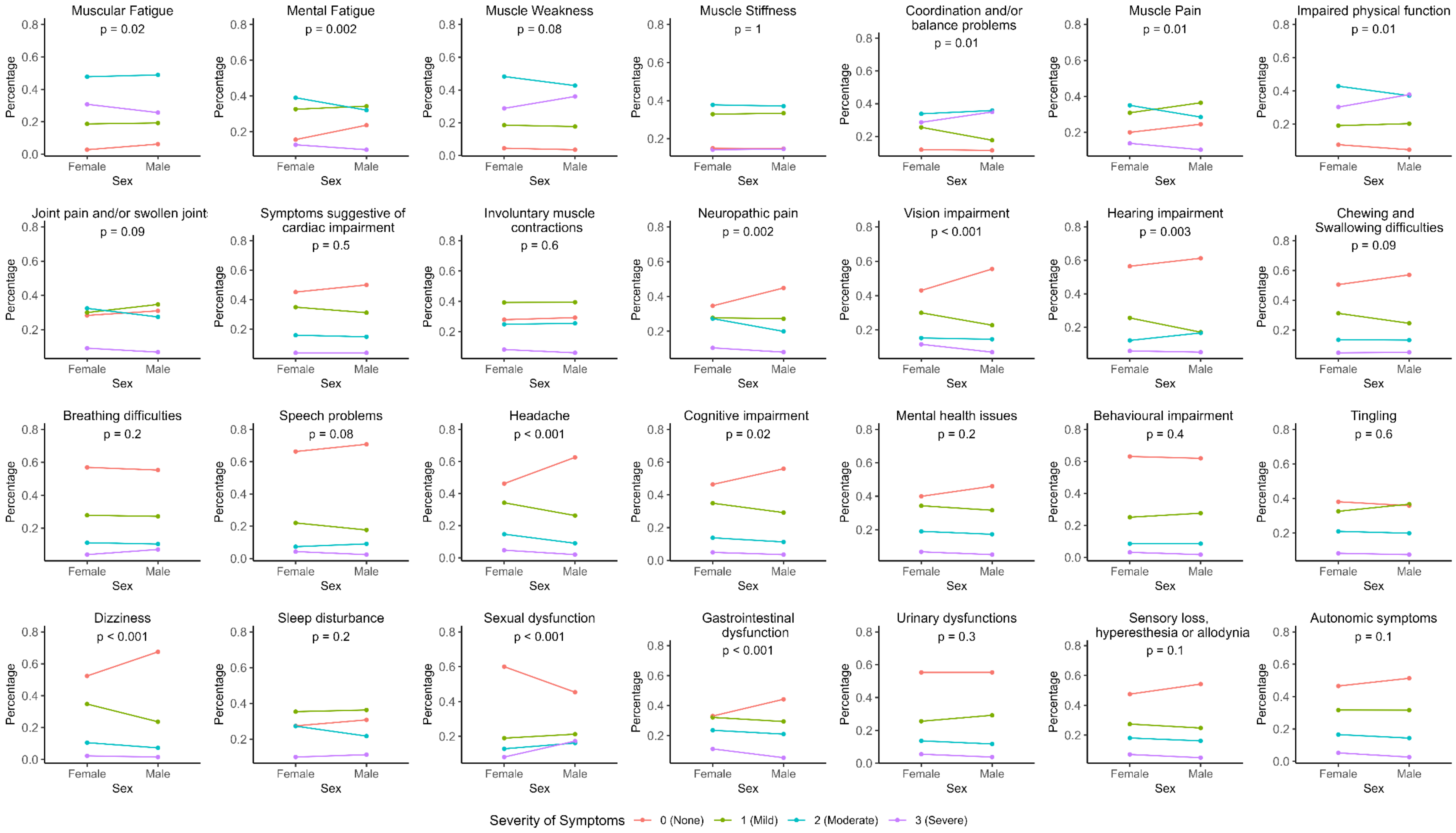

Entire cohort of NMD patients

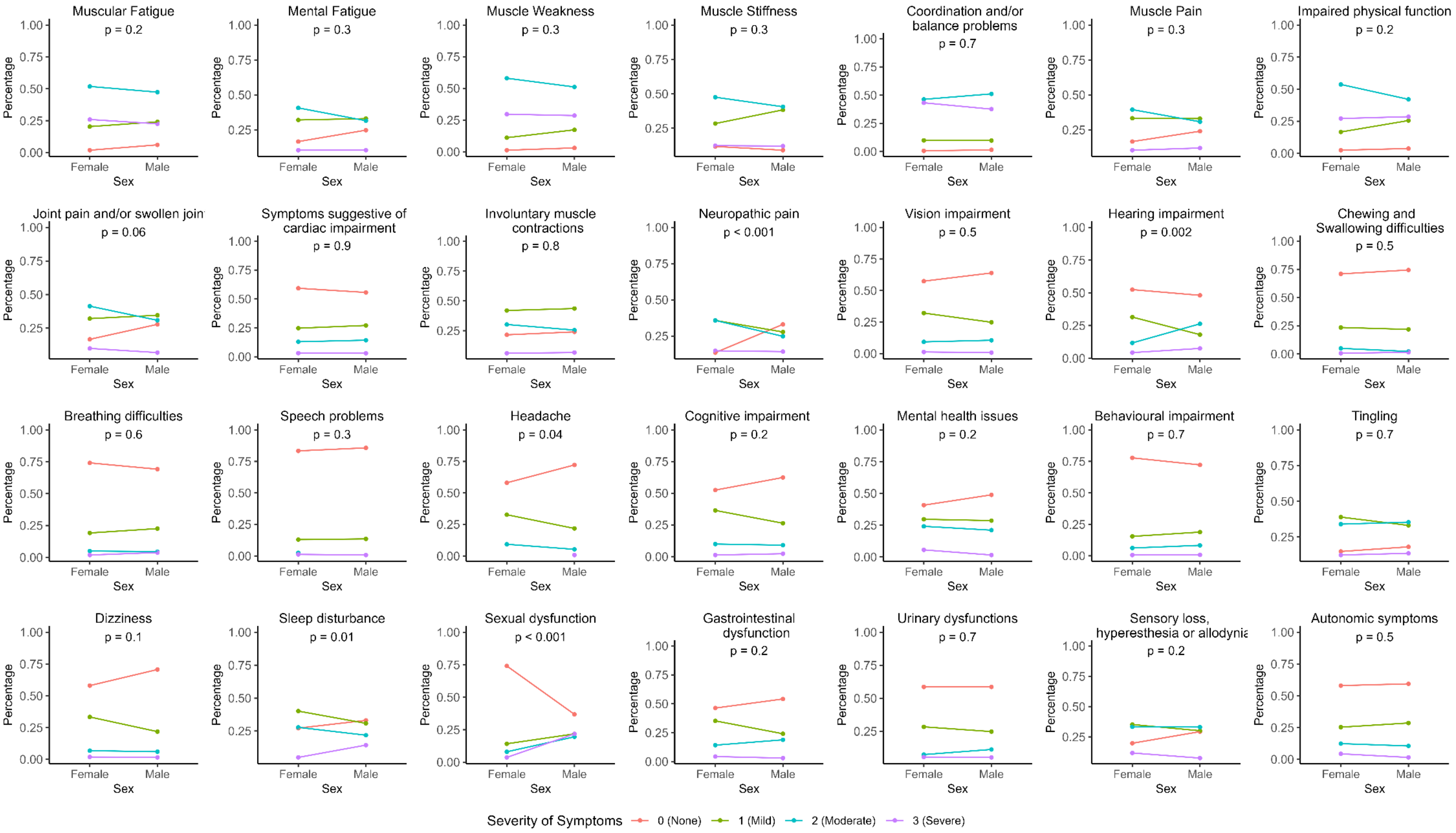

Charcot-Marie Tooth and related neuropathies HNNP HSAN dHMN

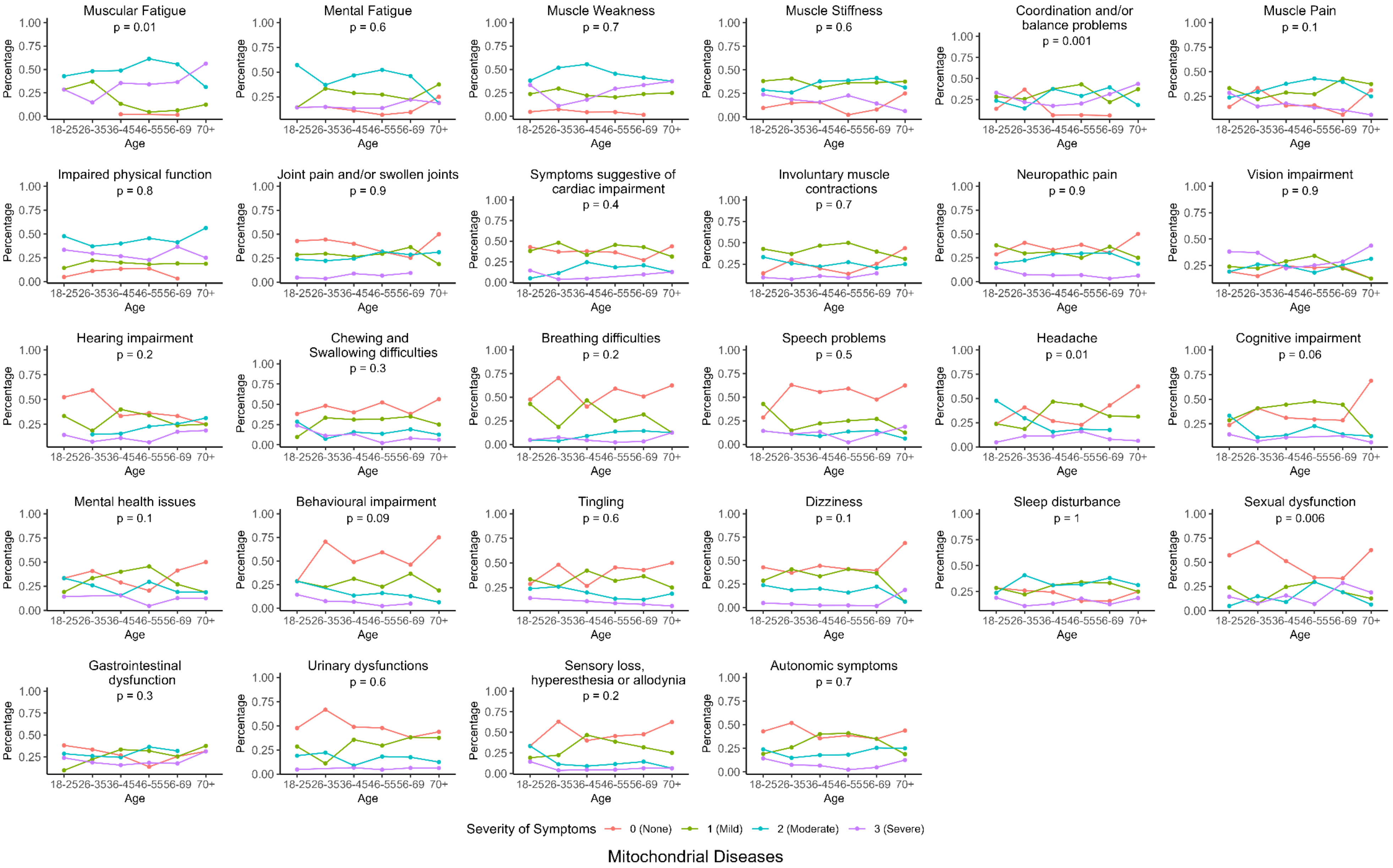
Muscular Fatigue
p = 0.01
Mental Fatigue
p = 0.6
Muscle Weakness
p = 0.7
Muscle Stiffness
p = 0.6
Coordination and/or balance problems
p = 0.001
Muscle Pain
p = 0.1
Impaired physical function
p = 0.8
Joint pain and/or swollen joints
p = 0.9
Symptoms suggestive of cardiac impairment
p = 0.4
Involuntary muscle contractions
p = 0.7
Neuropathic pain
p = 0.9
Vision impairment
p = 0.9
Hearing impairment
p = 0.2
Chewing and Swallowing difficulties
p = 0.3
Breathing difficulties
p = 0.2
Speech problems
p = 0.5
Headache
p = 0.01
Cognitive impairment
p = 0.06
Mental health issues
p = 0.1
Behavioural impairment
p = 0.09
Tingling
p = 0.6
Dizziness
p = 0.1
Sleep disturbance
p = 1
Sexual dysfunction
p = 0.006
Gastrointestinal dysfunction
p = 0.3
Urinary dysfunctions
p = 0.6
Sensory loss, hyperesthesia or allodynia
p = 0.2
Autonomic symptoms
p = 0.7
Percentage
0.00
0.25
0.50
0.75
1.00
Age
18-25
26-35
36-45
46-55
56-69
70+
Severity of Symptoms
0 (None)
1 (Mild)
2 (Moderate)
3 (Severe)
Mitochondrial Diseases

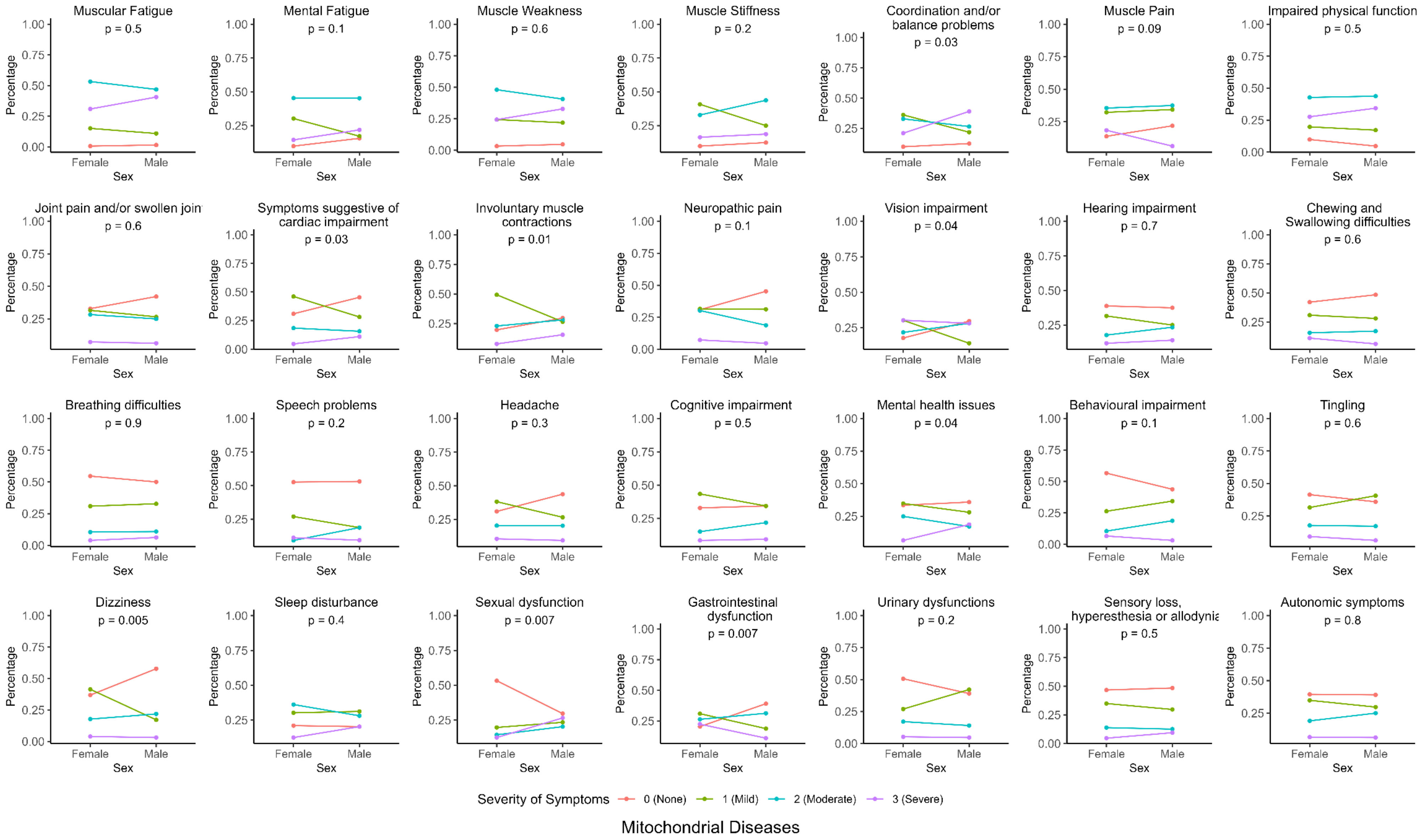
Muscular Fatigue
p = 0.5
Mental Fatigue
p = 0.1
Muscle Weakness
p = 0.6
Muscle Stiffness
p = 0.2
Coordination and/or balance problems
p = 0.03
Muscle Pain
p = 0.09
Impaired physical function
p = 0.5
Joint pain and/or swollen join
p = 0.6
Symptoms suggestive of cardiac impairment
p = 0.03
Involuntary muscle contractions
p = 0.01
Neuropathic pain
p = 0.1
Vision impairment
p = 0.04
Hearing impairment
p = 0.7
Chewing and Swallowing difficulties
p = 0.6
Breathing difficulties
p = 0.9
Speech problems
p = 0.2
Headache
p = 0.3
Cognitive impairment
p = 0.5
Mental health issues
p = 0.04
Behavioural impairment
p = 0.1
Tingling
p = 0.6
Dizziness
p = 0.005
Sleep disturbance
p = 0.4
Sexual dysfunction
p = 0.007
Gastrointestinal dysfunction
p = 0.007
Urinary dysfunctions
p = 0.2
Sensory loss, hyperesthesia or allodynia
p = 0.5
Autonomic symptoms
p = 0.8
Percentage
Sex
Female
Male
Severity of Symptoms
0 (None)
1 (Mild)
2 (Moderate)
3 (Severe)
Mitochondrial Diseases

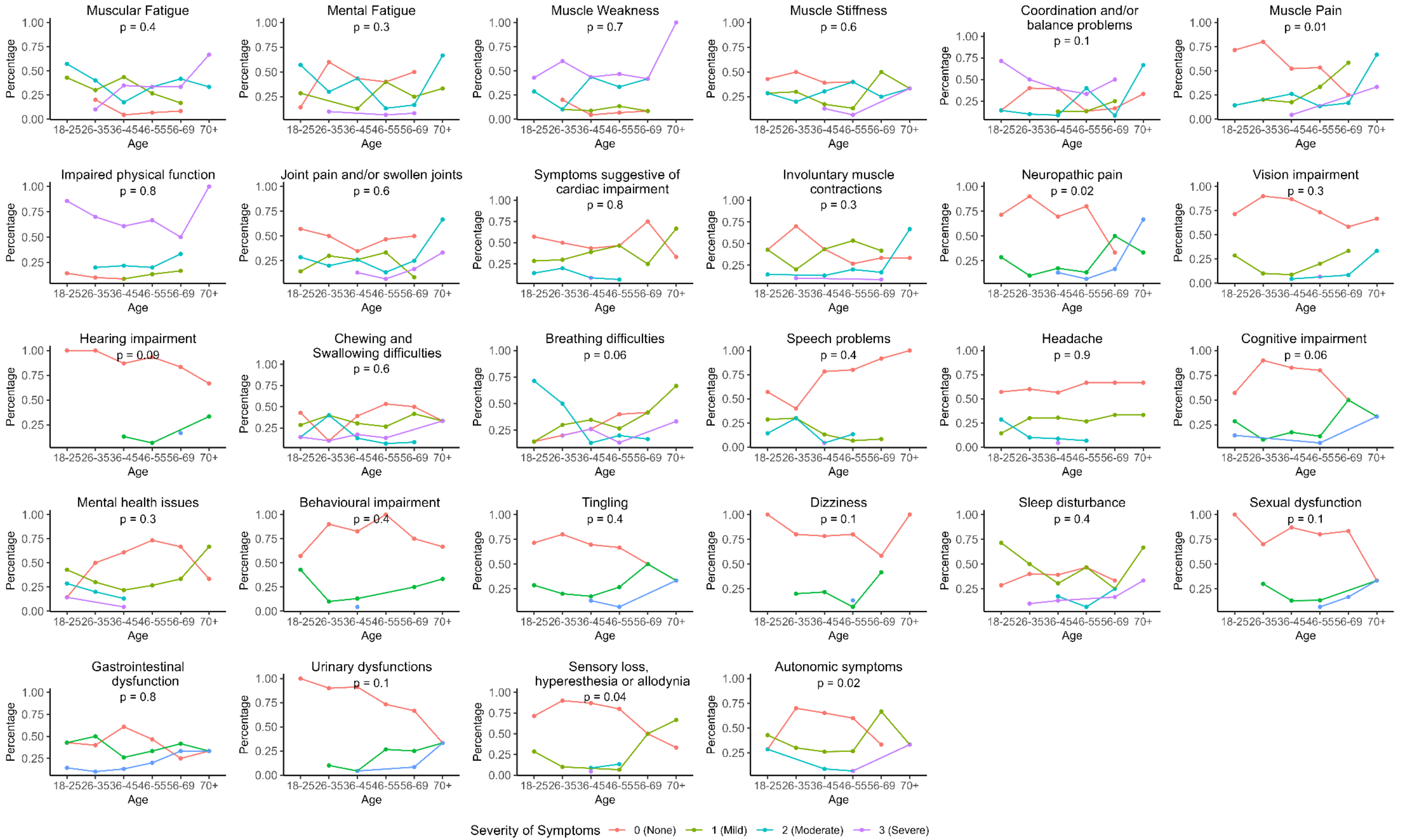

Spinal Muscular Atrophy SMA

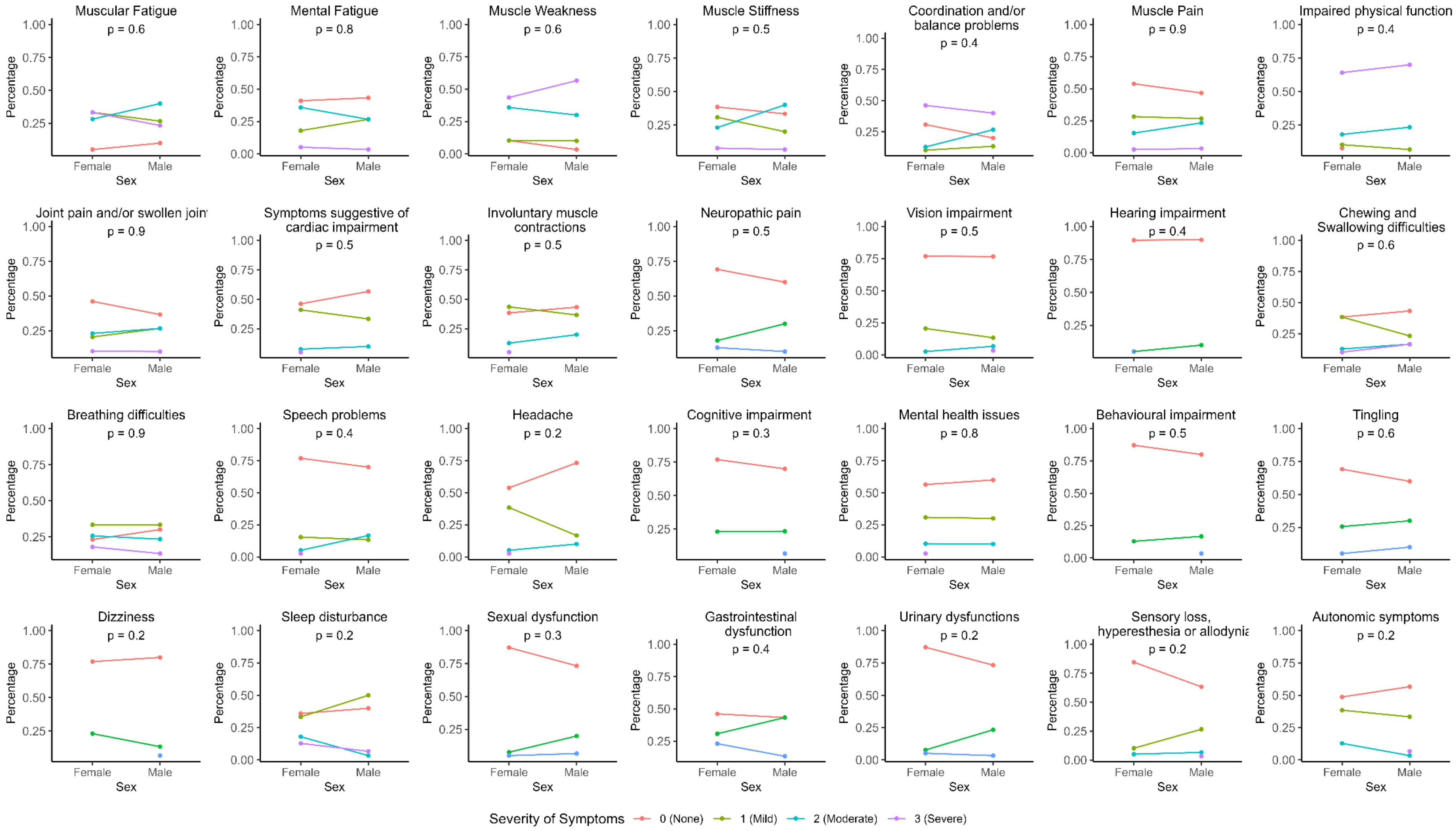

Spinal Muscular Atrophy SMA

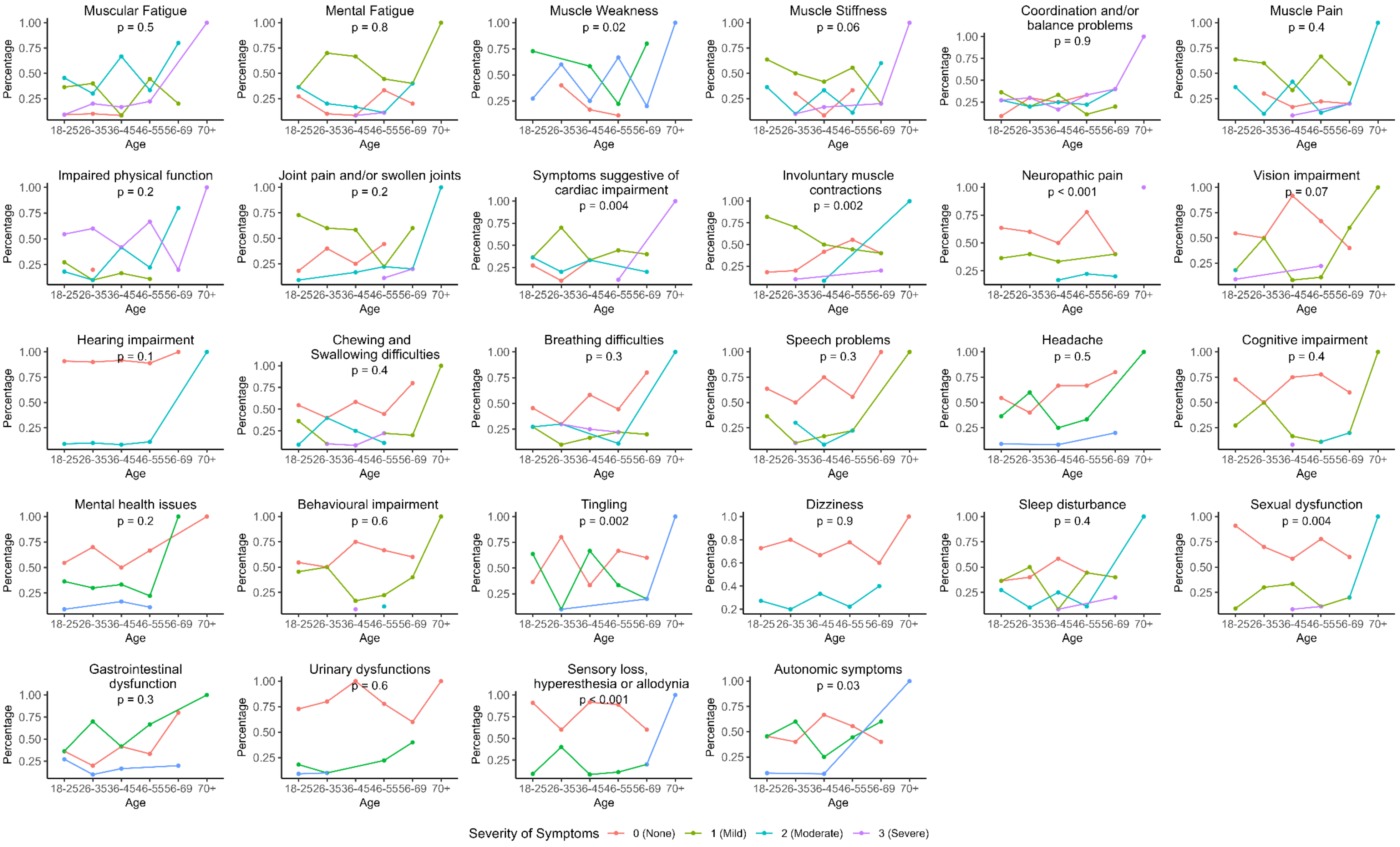

Duchenne or Becker Muscular Dystrophy

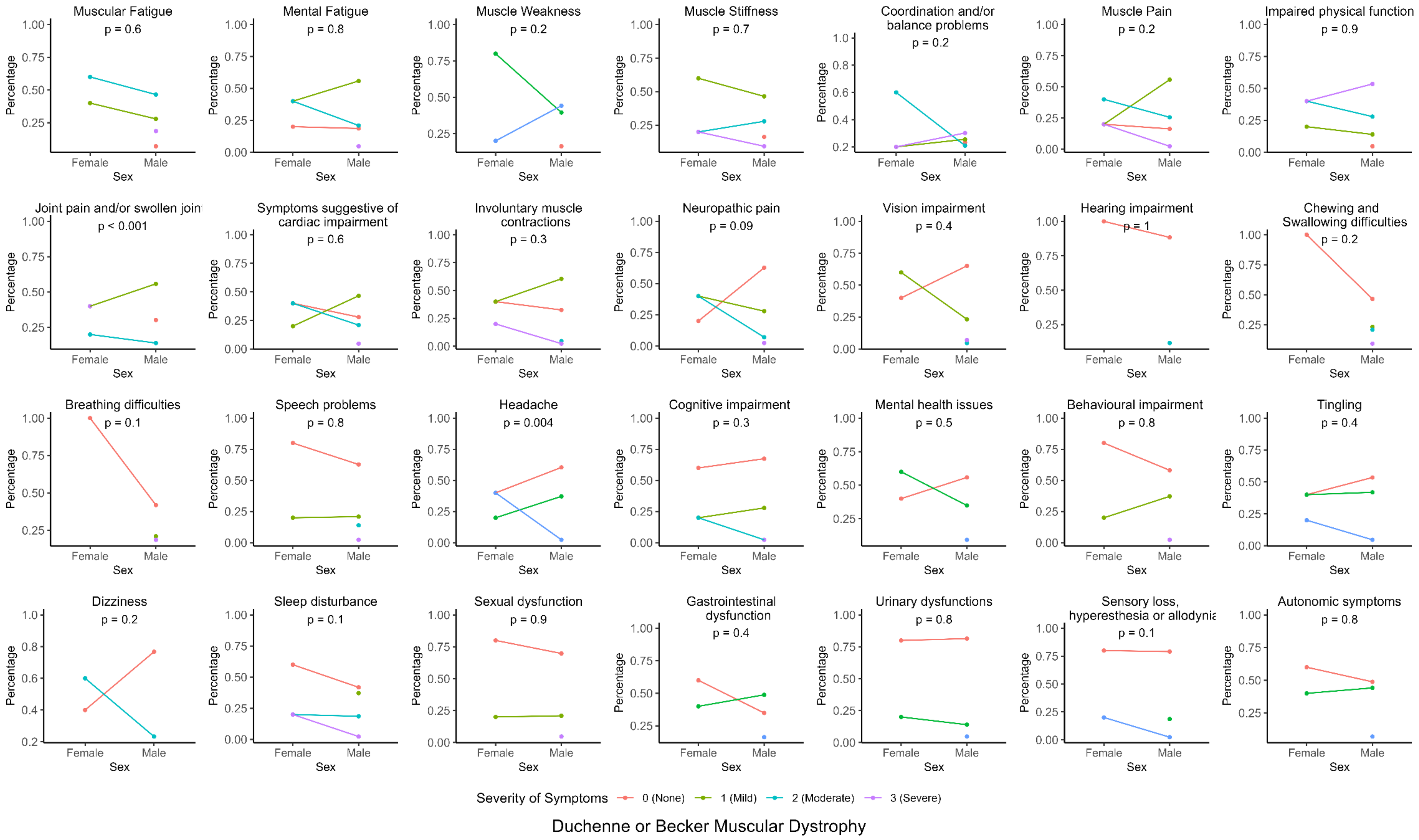
Muscular Fatigue
p = 0.6
Mental Fatigue
p = 0.8
Muscle Weakness
p = 0.2
Muscle Stiffness
p = 0.7
Coordination and/or balance problems
p = 0.2
Muscle Pain
p = 0.2
Impaired physical function
p = 0.9
Joint pain and/or swollen join
p < 0.001
Symptoms suggestive of cardiac impairment
p = 0.6
Involuntary muscle contractions
p = 0.3
Neuropathic pain
p = 0.09
Vision impairment
p = 0.4
Hearing impairment
p = 1
Chewing and Swallowing difficulties
p = 0.2
Breathing difficulties
p = 0.1
Speech problems
p = 0.8
Headache
p = 0.004
Cognitive impairment
p = 0.3
Mental health issues
p = 0.5
Behavioural impairment
p = 0.8
Tingling
p = 0.4
Dizziness
p = 0.2
Sleep disturbance
p = 0.1
Sexual dysfunction
p = 0.9
Gastrointestinal dysfunction
p = 0.4
Urinary dysfunctions
p = 0.8
Sensory loss, hyperesthesia or allodynia
p = 0.1
Autonomic symptoms
p = 0.8
Percentage
Sex
Female
Male
Severity of Symptoms
0 (None)
1 (Mild)
2 (Moderate)
3 (Severe)
Duchenne or Becker Muscular Dystrophy

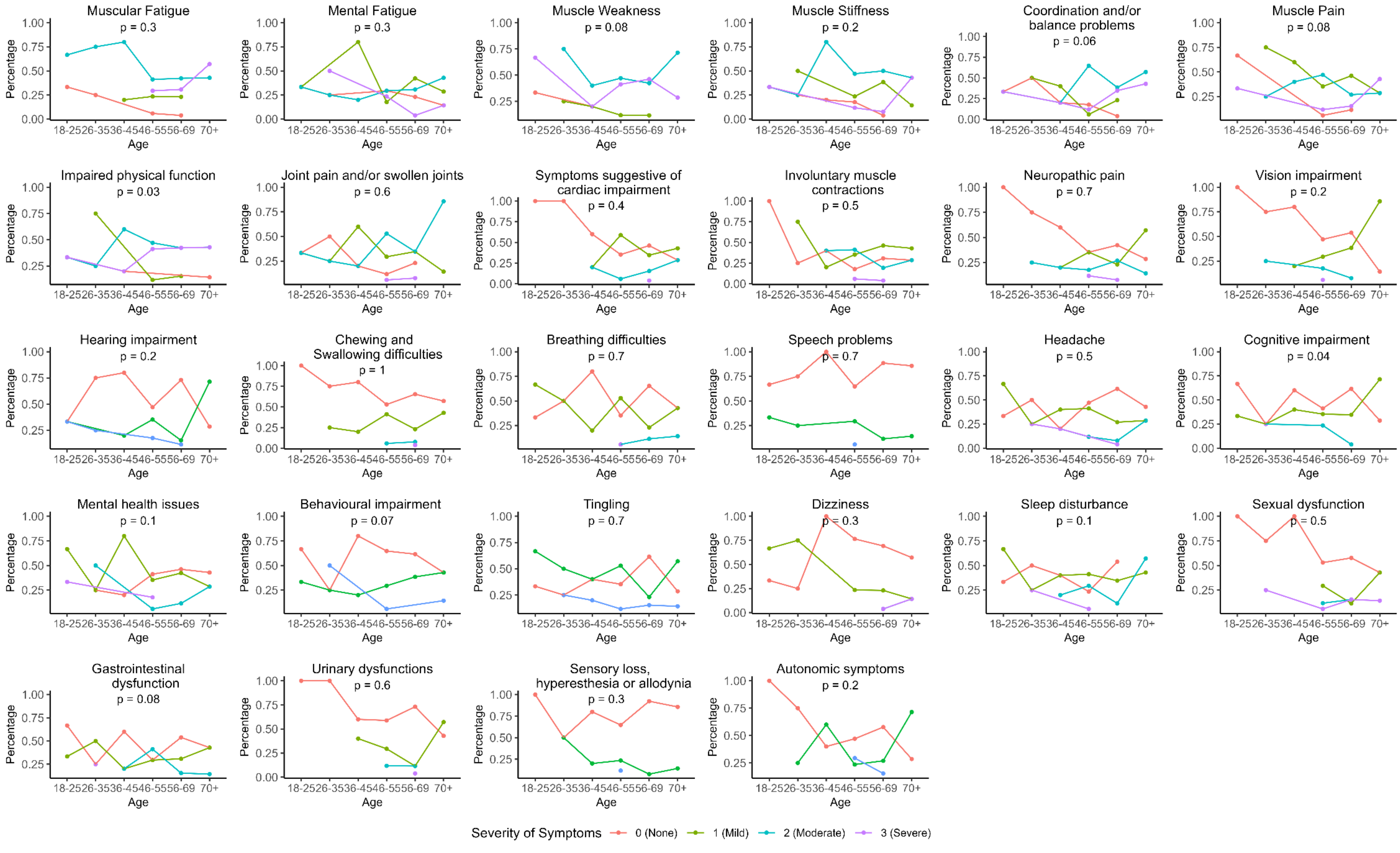

Facioscapulohumeral Muscular Dystrophy FSHD

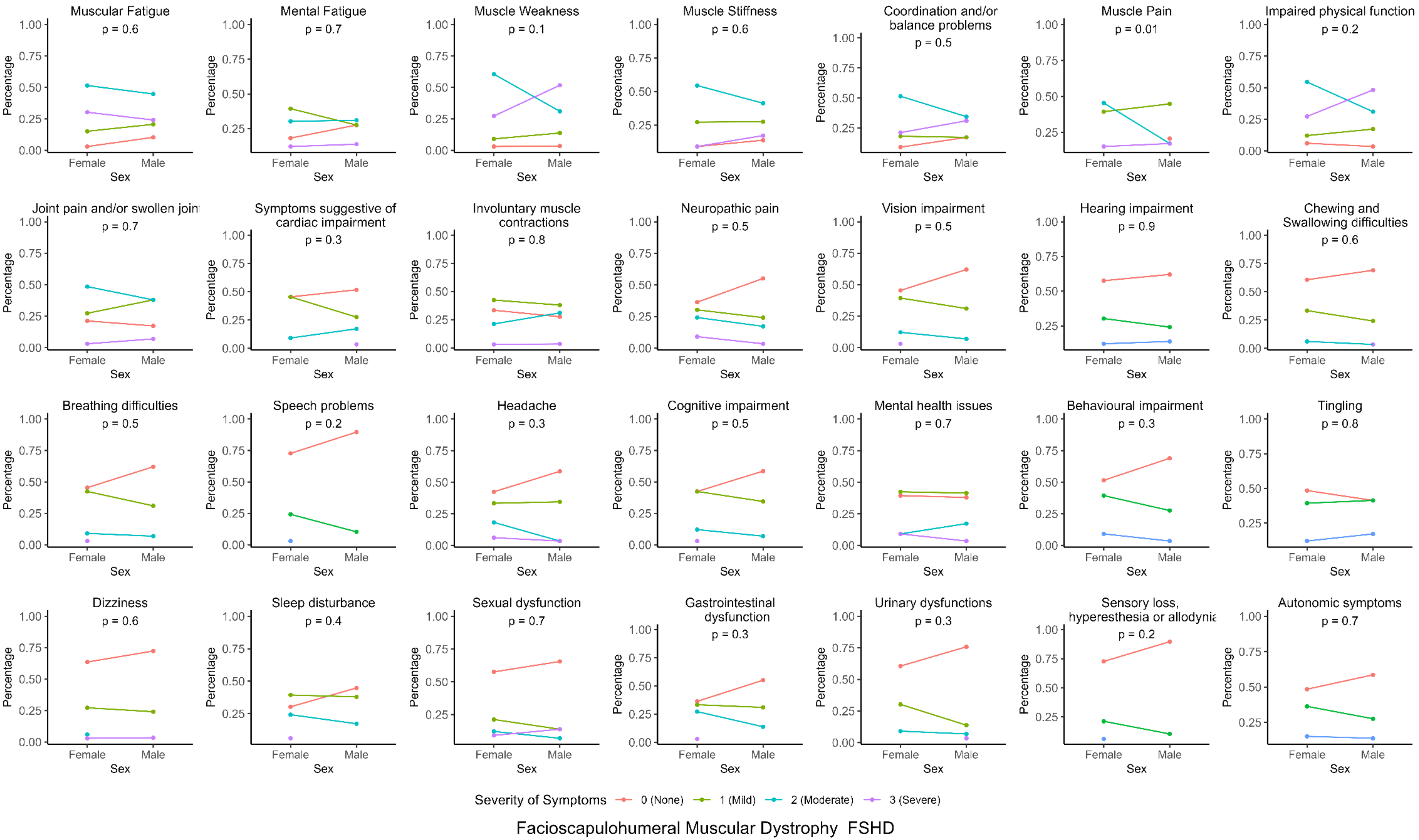
Muscular Fatigue p = 0.6
Mental Fatigue p = 0.7
Muscle Weakness p = 0.1
Muscle Stiffness p = 0.6
Coordination and/or balance problems p = 0.5
Muscle Pain p = 0.01
Impaired physical function p = 0.2
Joint pain and/or swollen join p = 0.7
Symptoms suggestive of cardiac impairment p = 0.3
Involuntary muscle contractions p = 0.8
Neuropathic pain p = 0.5
Vision impairment p = 0.5
Hearing impairment p = 0.9
Chewing and Swallowing difficulties p = 0.6
Breathing difficulties p = 0.5
Speech problems p = 0.2
Headache p = 0.3
Cognitive impairment p = 0.5
Mental health issues p = 0.7
Behavioural impairment p = 0.3
Tingling p = 0.8
Dizziness p = 0.6
Sleep disturbance p = 0.4
Sexual dysfunction p = 0.7
Gastrointestinal dysfunction p = 0.3
Urinary dysfunctions p = 0.3
Sensory loss, hyperesthesia or allodynia p = 0.2
Autonomic symptoms p = 0.7
Percentage
Sex
Female
Male
Severity of Symptoms
0 (None)
1 (Mild)
2 (Moderate)
3 (Severe)
Facioscapulohumeral Muscular Dystrophy FSHD

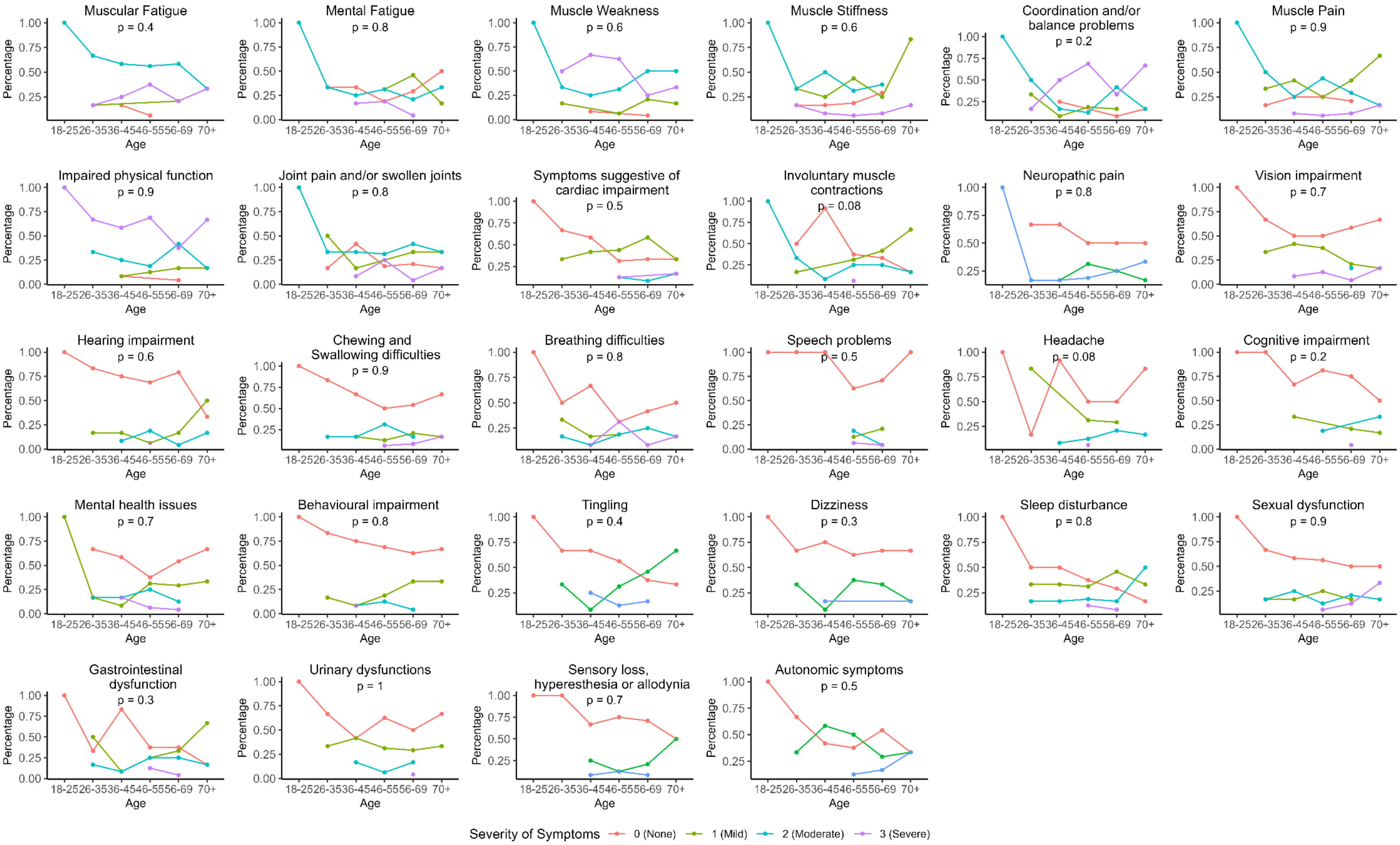

Other Muscular Dystrophies excluding Duchenne Becker FSHD myotonic dystrophies

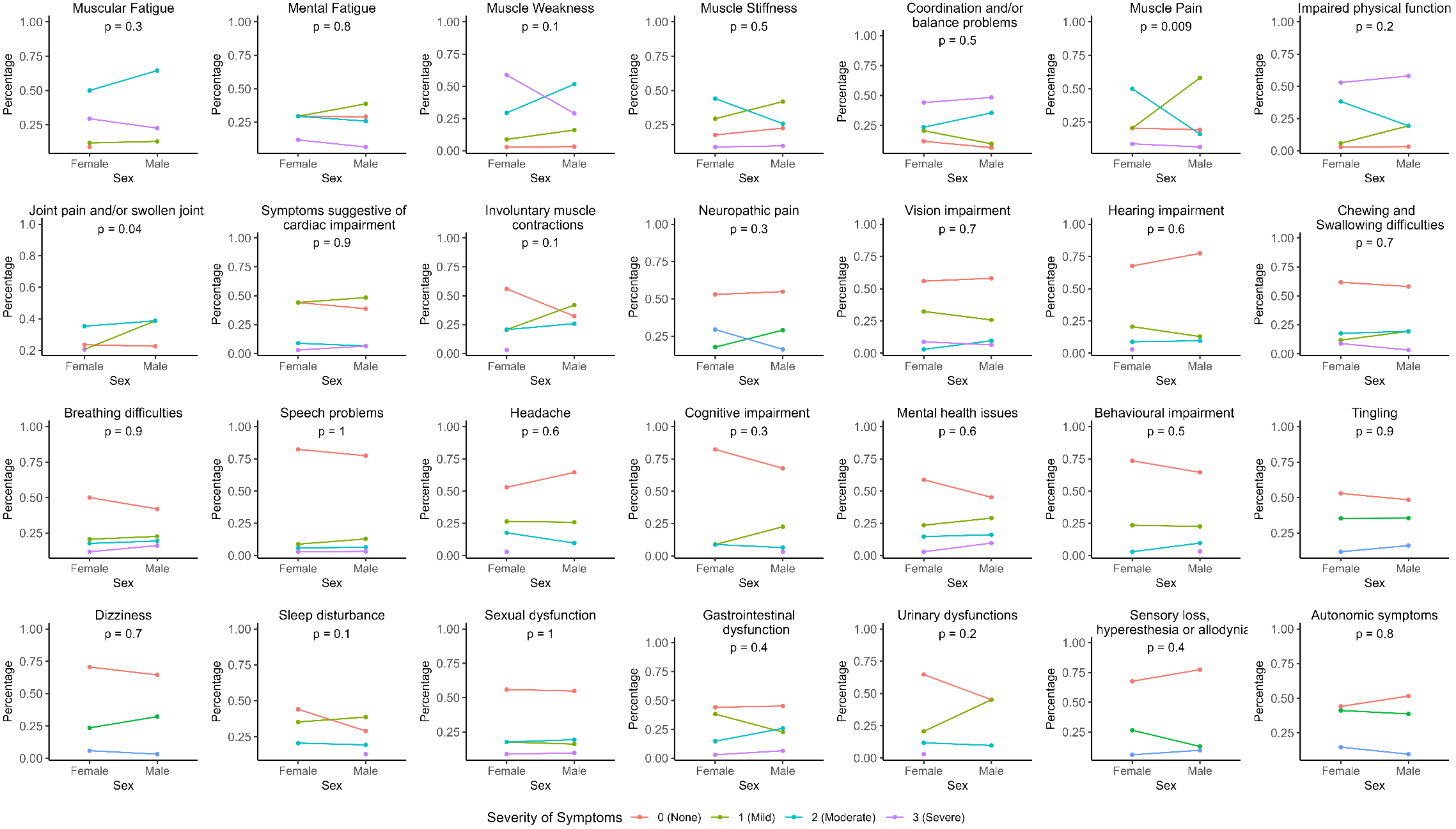

Other Muscular Dystrophies excluding Duchenne Becker FSHD myotonic dystrophies

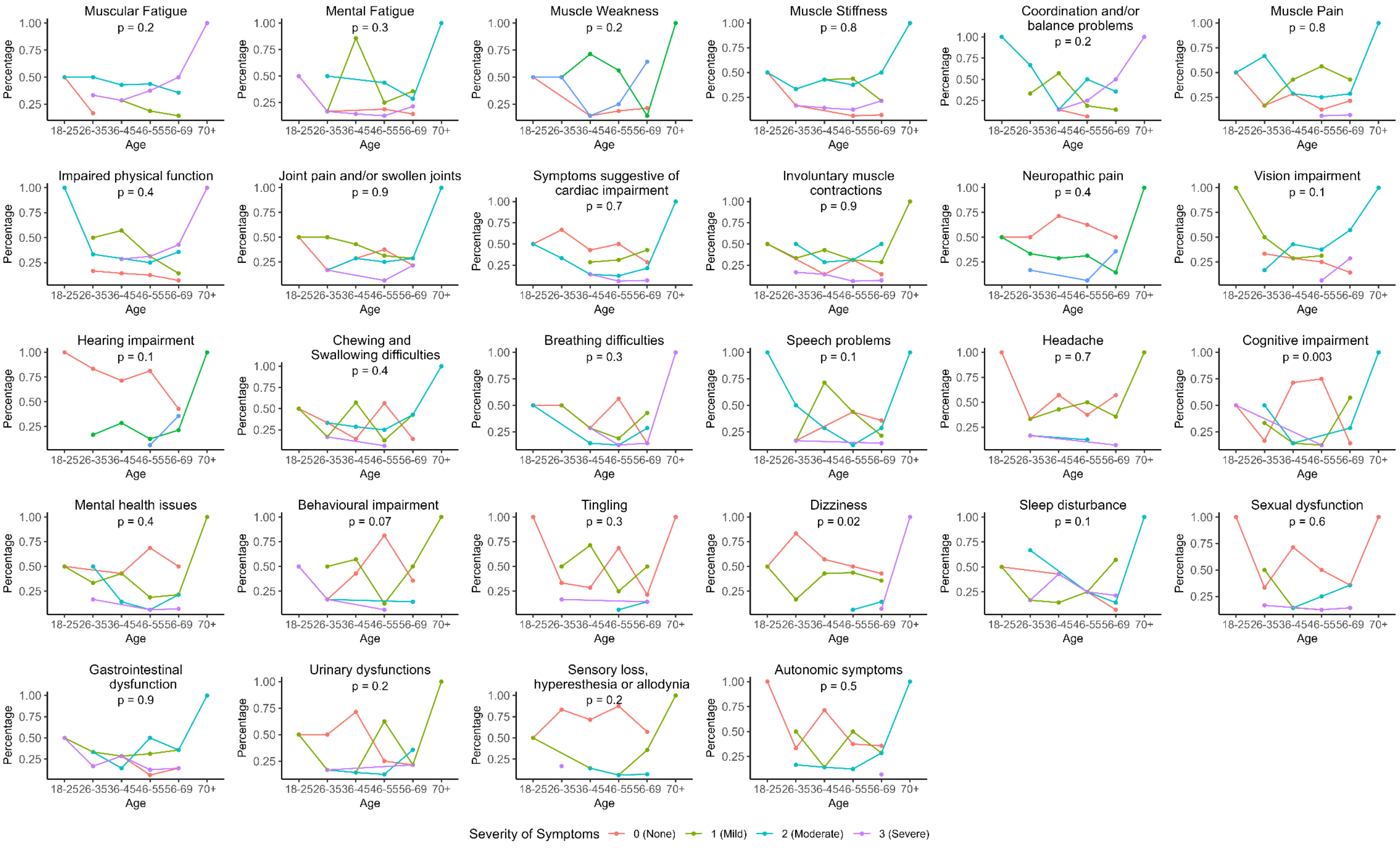

Myotonic Dystrophies

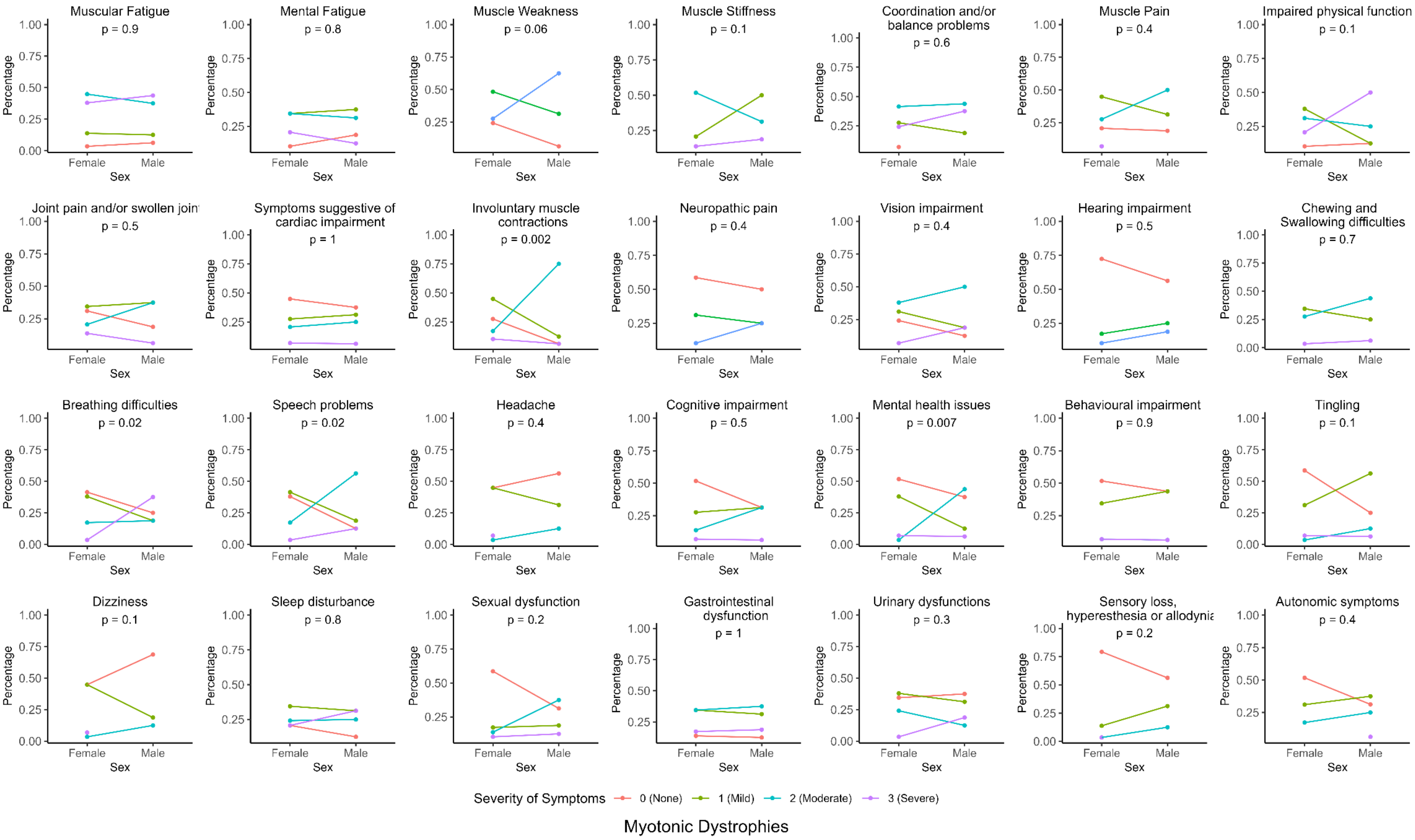
Muscular Fatigue
p = 0.9
Mental Fatigue
p = 0.8
Muscle Weakness
p = 0.06
Muscle Stiffness
p = 0.1
Coordination and/or balance problems
p = 0.6
Muscle Pain
p = 0.4
Impaired physical function
p = 0.1
Joint pain and/or swollen join
p = 0.5
Symptoms suggestive of cardiac impairment
p = 1
Involuntary muscle contractions
p = 0.002
Neuropathic pain
p = 0.4
Vision impairment
p = 0.4
Hearing impairment
p = 0.5
Chewing and Swallowing difficulties
p = 0.7
Breathing difficulties
p = 0.02
Speech problems
p = 0.02
Headache
p = 0.4
Cognitive impairment
p = 0.5
Mental health issues
p = 0.007
Behavioural impairment
p = 0.9
Tingling
p = 0.1
Dizziness
p = 0.1
Sleep disturbance
p = 0.8
Sexual dysfunction
p = 0.2
Gastrointestinal dysfunction
p = 1
Urinary dysfunctions
p = 0.3
Sensory loss, hyperesthesia or allodynia
p = 0.2
Autonomic symptoms
p = 0.4
Percentage
Sex
Female
Male
0.00
0.25
0.50
0.75
1.00
Severity of Symptoms
0 (None)
1 (Mild)
2 (Moderate)
3 (Severe)
Myotonic Dystrophies

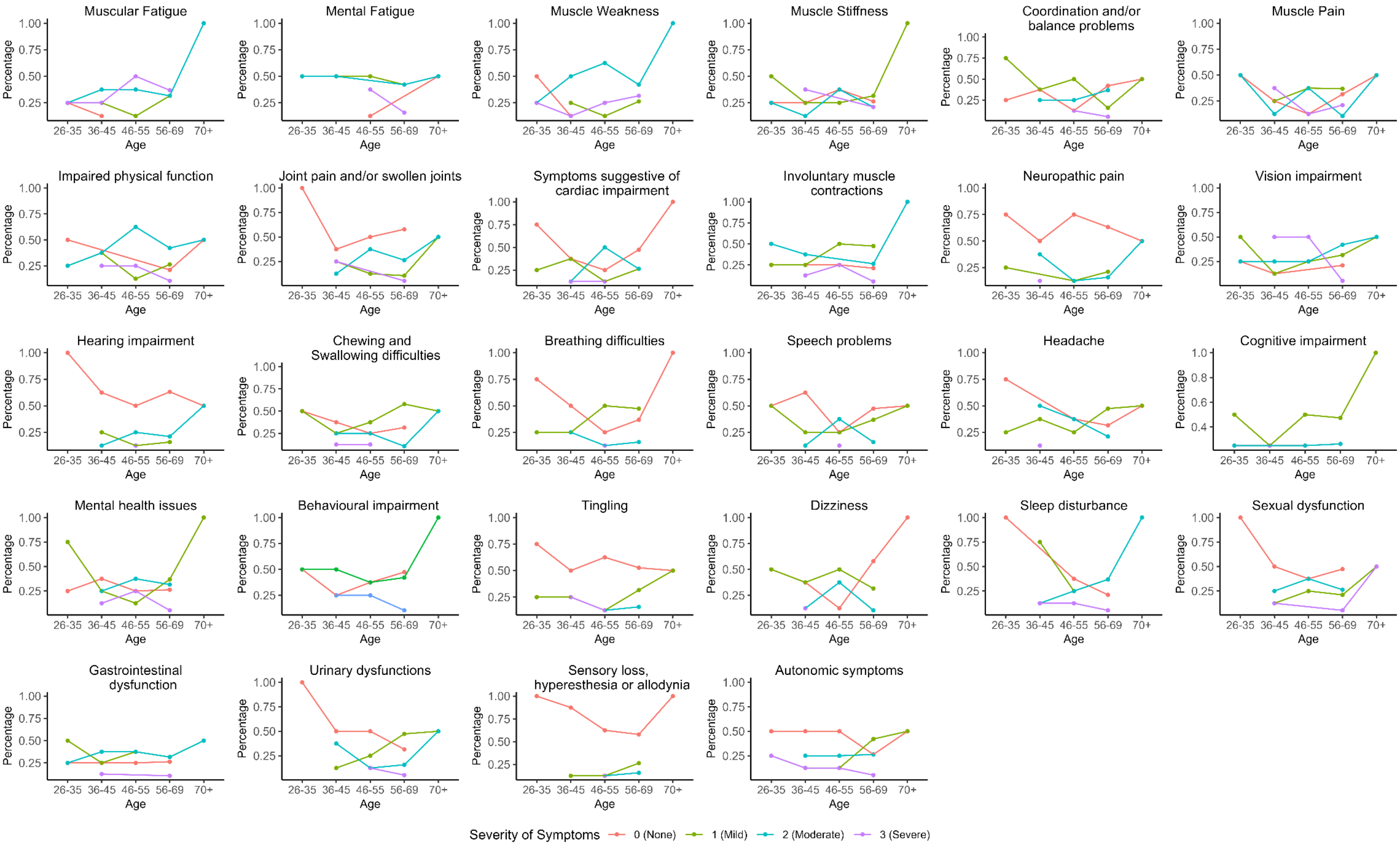

Myasthenia gravis

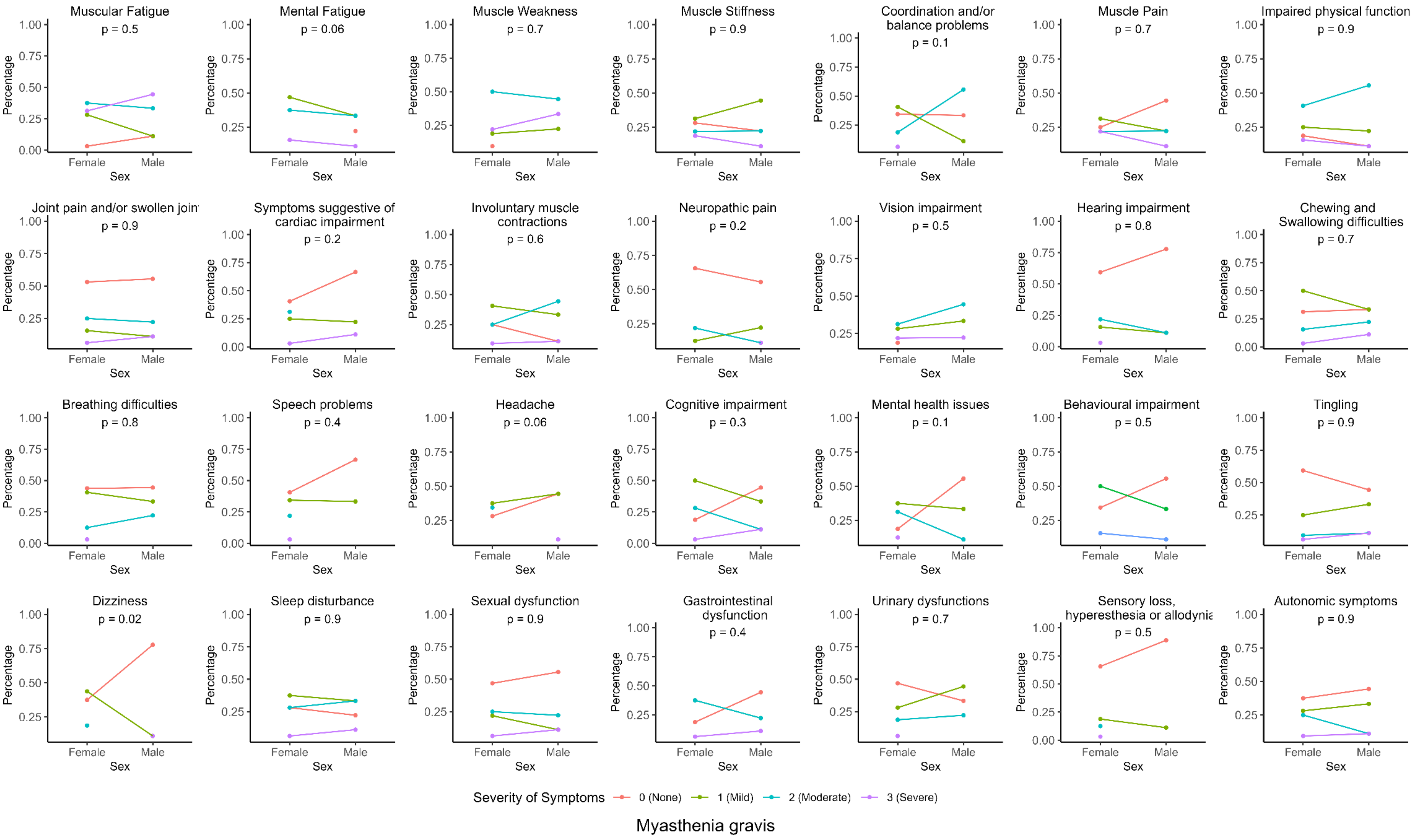
Muscular Fatigue
p = 0.5
Mental Fatigue
p = 0.06
Muscle Weakness
p = 0.7
Muscle Stiffness
p = 0.9
Coordination and/or balance problems
p = 0.1
Muscle Pain
p = 0.7
Impaired physical function
p = 0.9
Joint pain and/or swollen join
p = 0.9
Symptoms suggestive of cardiac impairment
p = 0.2
Involuntary muscle contractions
p = 0.6
Neuropathic pain
p = 0.2
Vision impairment
p = 0.5
Hearing impairment
p = 0.8
Chewing and Swallowing difficulties
p = 0.7
Breathing difficulties
p = 0.8
Speech problems
p = 0.4
Headache
p = 0.06
Cognitive impairment
p = 0.3
Mental health issues
p = 0.1
Behavioural impairment
p = 0.5
Tingling
p = 0.9
Dizziness
p = 0.02
Sleep disturbance
p = 0.9
Sexual dysfunction
p = 0.9
Gastrointestinal dysfunction
p = 0.4
Urinary dysfunctions
p = 0.7
Sensory loss, hyperesthesia or allodynia
p = 0.5
Autonomic symptoms
p = 0.9
Percentage
Sex
Female
Male
Severity of Symptoms
0 (None)
1 (Mild)
2 (Moderate)
3 (Severe)
Myasthenia gravis

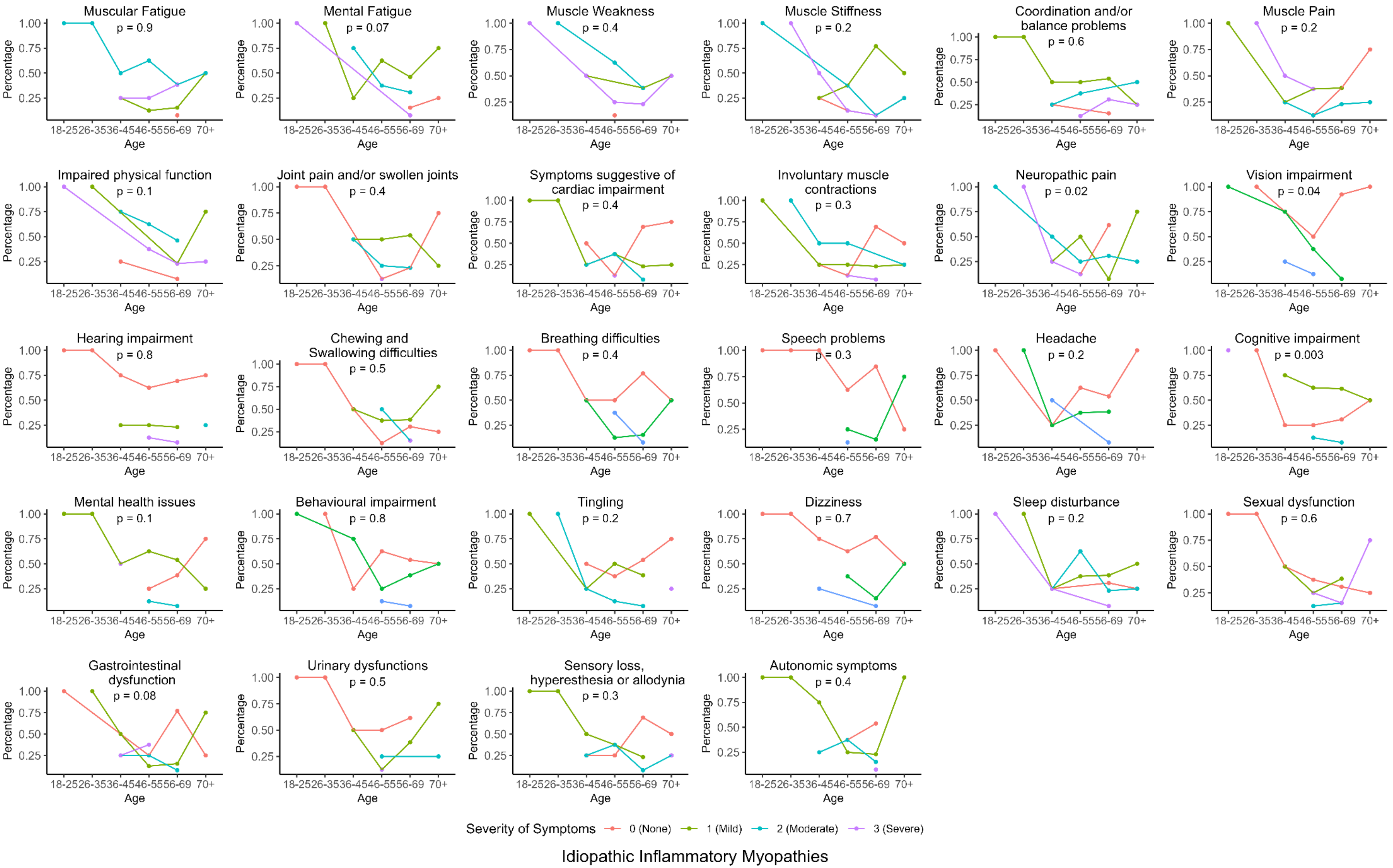
Muscular Fatigue
p = 0.9
Mental Fatigue
p = 0.07
Muscle Weakness
p = 0.4
Muscle Stiffness
p = 0.2
Coordination and/or balance problems
p = 0.6
Muscle Pain
p = 0.2
Impaired physical function
p = 0.1
Joint pain and/or swollen joints
p = 0.4
Symptoms suggestive of cardiac impairment
p = 0.4
Involuntary muscle contractions
p = 0.3
Neuropathic pain
p = 0.02
Vision impairment
p = 0.04
Hearing impairment
p = 0.8
Chewing and Swallowing difficulties
p = 0.5
Breathing difficulties
p = 0.4
Speech problems
p = 0.3
Headache
p = 0.2
Cognitive impairment
p = 0.003
Mental health issues
p = 0.1
Behavioural impairment
p = 0.8
Tingling
p = 0.2
Dizziness
p = 0.7
Sleep disturbance
p = 0.2
Sexual dysfunction
p = 0.6
Gastrointestinal dysfunction
p = 0.08
Urinary dysfunctions
p = 0.5
Sensory loss, hyperesthesia or allodynia
p = 0.3
Autonomic symptoms
p = 0.4
Percentage
Age
18-25 26-35 36-45 46-55 56-69 70+
Severity of Symptoms
0 (None)
1 (Mild)
2 (Moderate)
3 (Severe)
Idiopathic Inflammatory Myopathies

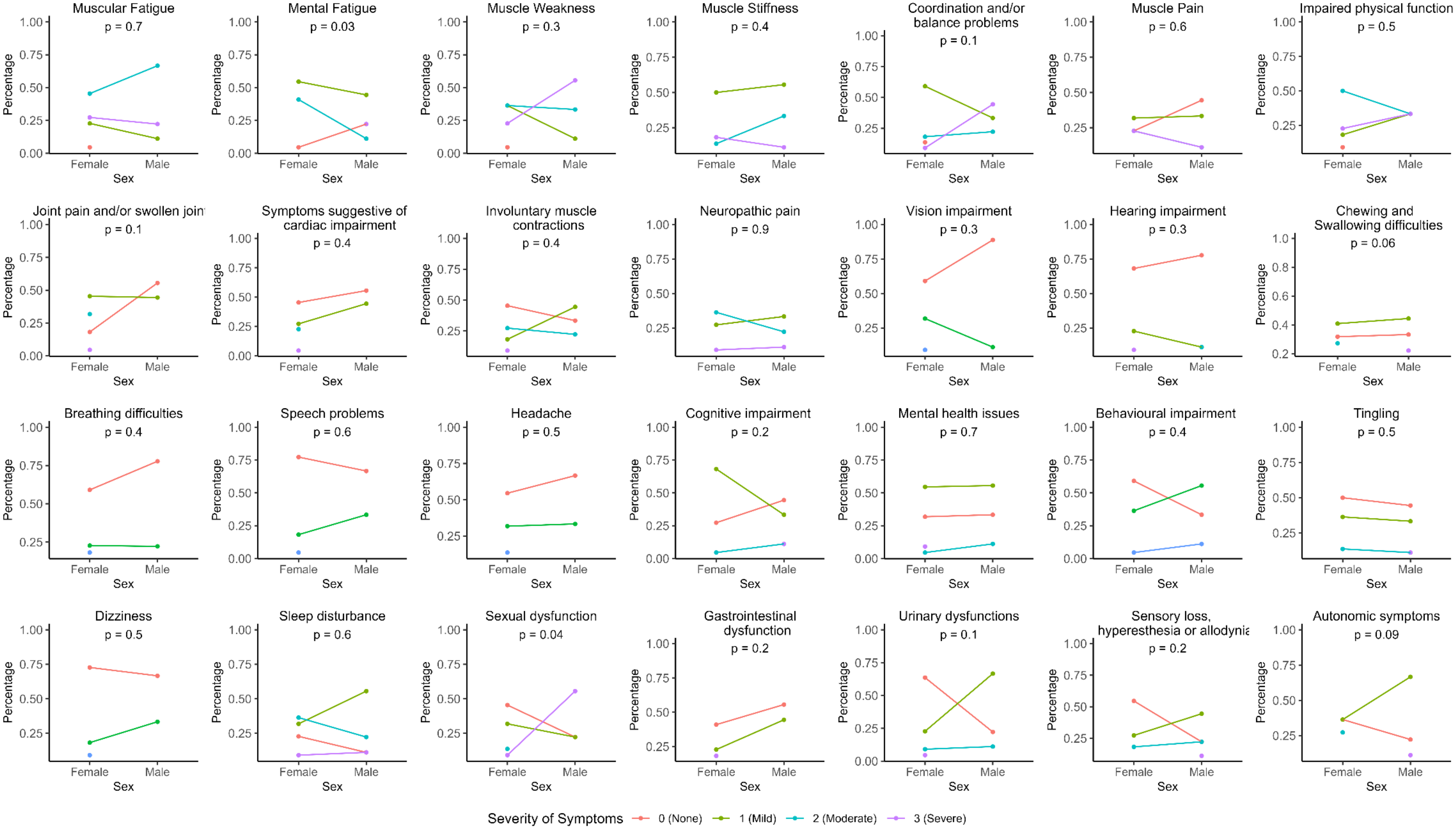

Idiopathic Inflammatory Myopathies
