# Supplementary material for: The most bothersome symptoms in neuromuscular diseases: the ERN EURO NMD Survey
Source: Orphanet J Rare Dis. 2025 May 8;20:221. doi: 10.1186/s13023-025-03742-z (PMC12063438; doi:10.1186/s13023-025-03742-z)

## ERN NMD PROM FOR ADULTS

**Welcome to the Assessment of the Neuromuscular Disease (NMD) patients' symptom burden**

**Thank you for participating in our survey. Your feedback is important.**

***This survey will allow us to better identify your symptom burden!***

**This survey is only for adult patients (18 years and older)!**

**The survey must be completed by the patient only! If you are not able to complete it, please ask help to your caregiver!**

**Data protection. This is an anonymous survey.**

**We are not collecting any personal data and we will not share or make public information that can link you to individual responses presented in our final report. Please continue the survey if you agree on that thank you**

## ERN NMD PROM FOR ADULTS

***Assessment of the symptom burden (the symptom that most negatively affect patient's daily life) in NMD patients***

* 1. **What is your gender?**

- ◯ Male
- ◯ Female
- ◯ Prefer not to say

* 2. **What is your age?**

- ◯ 18-25
- ◯ 26-35
- ◯ 36-45
- ◯ 46-55
- ◯ 56-69
- ◯ 70 and older

* 3. Where do you live?

- Austria
- Belgium
- Bulgaria
- Croatia
- Cyprus
- Czech Republic
- Denmark
- Estonia
- Finland
- France
- Germany
- Greece
- Hungary
- Ireland
- Italy
- Latvia
- Lithuania
- Luxembourg
- Malta
- Netherlands
- Norway
- Poland
- Portugal
- Romania
- Slovakia
- Slovenia
- Spain
- Sweden
- UK
- Other

* 4. **Have you been diagnosed with one of the following diseases?**

- ◯ Mitochondrial Diseases
- ◯ Duchenne or Becker Muscular Dystrophy
- ◯ Facioscapulohumeral Muscular Dystrophy (FSHD)
- ◯ Myotonic Dystrophies
- ◯ Other Muscular Dystrophies (excluding Duchenne, Becker, FSHD, myotonic dystrophies)
- ◯ Metabolic Myopathies
- ◯ Idiopathic Inflammatory Myopathies
- ◯ Myofibrillar Myopathies
- ◯ Congenital Myopathies and Congenital muscular dystrophies
- ◯ Skeletal Muscle Channelopathies
- ◯ Amyotrophic Lateral Sclerosis and other motor neuron diseases (excluding SMA)
- ◯ Spinal Muscular Atrophy (SMA)
- ◯ Myasthenia gravis
- ◯ Congenital Myasthenic Syndromes
- ◯ Charcot-Marie Tooth and related neuropathies (HNNP, HSAN, dHMN)
- ◯ Hereditary Amyloid Neuropathy
- ◯ Neuropathies associated with haematological disease and monoclonal gammopathy (MGUS, POEMS, ETC)
- ◯ Inflammatory and Dysimmune Neuropathies
- ◯ Small Fibre Neuropathies
- ◯ Idiopathic Neuropathies
- ◯ I do not know the name of my disease
- ◯ Other

European Reference Network for rare or low prevalence complex diseases — Network Neuromuscular Diseases (ERN EURO-NMD)

EURO-NMD | Building bridges and breaking barriers in rare neuromuscular diseases

## ERN NMD PROM FOR ADULTS

**In this page, you should provide an answer to all queries, quantifying how much a specific symptom (or a group of symptoms) is affecting your daily life**

* 5. **Muscular Fatigue** (i.e., overwhelming sense of tiredness, lack of energy and feeling exhausted)

- ◯ 0 (None)
- ◯ 1 (Mild)
- ◯ 2 (Moderate)
- ◯ 3 (Severe)

* 6. **Mental fatigue** (Mental fatigue is a psychophysiological state that occurs after or during prolonged periods of cognitive activity; it is a component of subjective fatigue and is characterized by self-reported feelings of tiredness, lack of motivation, and decreased cognitive performance, feeling a lack of interest in normal activities)

- 0 (None)
- 1 (Mild)
- 2 (Moderate)
- 3 (Severe)

* 7. **Muscle Weakness** (decrease in muscle strength, i.e., reduced ability to move your body; it may include facial or other specific muscle weakness)

- 0 (None)
- 1 (Mild)
- 2 (Moderate)
- 3 (Severe)

* 8. **Muscle stiffness** (defined as the sensation of tight, cramped or painful muscles)

- 0 (None)
- 1 (Mild)
- 2 (Moderate)
- 3 (Severe)

* 9. **Coordination and/or balance problems** (i.e., difficulty with walking and balance, hand coordination, falling)

- 0 (None)
- 1 (Mild)
- 2 (Moderate)
- 3 (Severe)

* 10. **Muscle Pain**

- 0 (None)
- 1 (Mild)
- 2 (Moderate)
- 3 (Severe)

* 11. **Impaired physical function/activity**

- 0 (None)
- 1 (Mild)
- 2 (Moderate)
- 3 (Severe)

* 12. **Joint pain and/or swollen joints**

- 0 (None)
- 1 (Mild)
- 2 (Moderate)
- 3 (Severe)

* 13. **Symptoms suggestive of cardiac impairment** (i.e., palpitation, dyspnea, shortness of breath, light-headedness and collapse).

- 0 (None)
- 1 (Mild)
- 2 (Moderate)
- 3 (Severe)

* 14. **Involuntary muscle contractions:** i.e., cramps (a sudden, painful and unexpected tightening of one or more muscles), contractures (includes pain and loss of movement in the joint), fasciculations (small painless movement under the skin), mounding ( a local contracture, "bump", of skeletal muscle that is induced by tapping the muscle directly), rippling (wave-like muscle contraction and unusual sensitivity of the muscle to the movement or pressure), myokymia (involuntary quivering, rippling, and undulating muscle contractions), myotonia (not being able to relax a muscle after it contracts).

- 0 (None)
- 1 (Mild)
- 2 (Moderate)
- 3 (Severe)

* 15. **Neuropathic pain** (characterized by stabbing and shooting pain in affected area. Examples: ongoing or intermittent spontaneous pain, burning, pricking, squeezing sensation)

- 0 (None)
- 1 (Mild)
- 2 (Moderate)
- 3 (Severe)

* 16. **Vision impairment:** including dropping eyelid, impaired eye movement, blurred vision, diplopia (double vision), cataracts

- 0 (None)
- 1 (Mild)
- 2 (Moderate)
- 3 (Severe)

* 17. **Hearing impairment** (including troubles to hear clear sounds, hearing loss ranging from mild to severe, and tinnitus, which is commonly described as a ringing sound)

- 0 (None)
- 1 (Mild)
- 2 (Moderate)
- 3 (Severe)

* 18. **Chewing and Swallowing difficulties** (i.e., food stuck in the throat; being able to eat only piecemeal portion because it is very difficult to digest a meal; food or fluid come through the nose; chocking)

- 0 (None)
- 1 (Mild)
- 2 (Moderate)
- 3 (Severe)

* 19. **Breathing difficulties** (i.e., weak cough, secretions, lack of clearance often leading to aspiration pneumonia)

- 0 (None)
- 1 (Mild)
- 2 (Moderate)
- 3 (Severe)

* 20. **Speech problems**

- 0 (None)
- 1 (Mild)
- 2 (Moderate)
- 3 (Severe)

* 21. **Headache**

- 0 (None)
- 1 (Mild)
- 2 (Moderate)
- 3 (Severe)

* 22. **Cognitive impairment** (problems concentrating, multitasking, thinking, learning, or difficulties with memory or judgment)

- 0 (None)
- 1 (Mild)
- 2 (Moderate)
- 3 (Severe)

* 23. **Mental health issues** (i.e., mood changes, inability to manage stress and problem, feelings of fear, worry, or guilt, changes in eating habits, suicidal ideation, lack of understanding or perception of their own illness)

- 0 (None)
- 1 (Mild)
- 2 (Moderate)
- 3 (Severe)

* 24. **Behavioural impairment** (i.e., learning disability, lower performance in relation to adaptive behaviours, communication and socialization, problems with verbal working memory, attention, and executive function)

- 0 (None)
- 1 (Mild)
- 2 (Moderate)
- 3 (Severe)

* 25. **Tingling (an odd prickling sensation) and/or numbness (a loss of feeling in a part of the body)**

- 0 (None)
- 1 (Mild)
- 2 (Moderate)
- 3 (Severe)

* 26. **Dizziness**

- 0 (None)
- 1 (Mild)
- 2 (Moderate)
- 3 (Severe)

* 27. **Sleep disturbance** (i.e., excessive daytime sleepiness, insomnia, sleep apnea)

- 0 (None)
- 1 (Mild)
- 2 (Moderate)
- 3 (Severe)

* 28. **Sexual dysfunction**

- 0 (None)
- 1 (Mild)
- 2 (Moderate)
- 3 (Severe)

* 29. **Gastrointestinal dysfunction** (i.e., constipation, diarrhea, pseudo-obstruction, acid reflux, vomiting)

- 0 (None)
- 1 (Mild)
- 2 (Moderate)
- 3 (Severe)

* 30. **Urinary dysfunctions** (i.e., bladder dysfunction, urinary incontinence)

- 0 (None)
- 1 (Mild)
- 2 (Moderate)
- 3 (Severe)

* 31. **Subjective sensory loss or extreme sensitivity to** touch, temperature and/or pain

- 0 (None)
- 1 (Mild)
- 2 (Moderate)
- 3 (Severe)

32. **Autonomic symptoms** (i.e., changes in blood pressure, heart rate, sweating, and bladder or bowel dysfunction)

- 0 (None)
- 1 (Mild)
- 2 (Moderate)
- 3 (Severe)

* 33. Did we miss any other critical symptom affecting your daily life?

- Yes
- No

34. If you answered **YES** to question n. 33, please add one of those symptoms per row, up to three symptoms.

1.

2.

3.

**Thank you for completing the survey! You did great!**

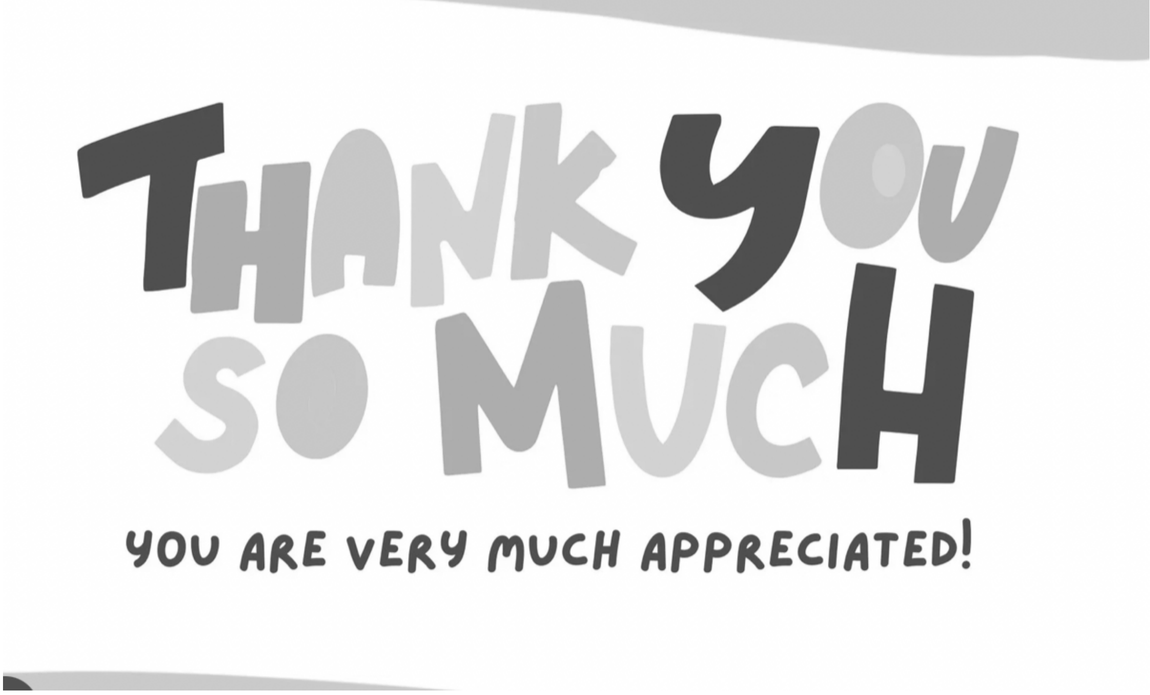

Supplement: Supplementary file 3 — Supplementary Material 3 [file 13023_2025_3742_MOESM3_ESM.pdf]
